# Computational Study of Ignored Pericyclic Reactions: Rearrangements of 1,2‐Bis(Diazo)Alkanes to 1,2,3,4‐Tetrazines and Subsequent Fragmentations

**DOI:** 10.1002/anie.202514598

**Published:** 2025-09-16

**Authors:** Hans‐Ulrich Reissig, Ernst‐Ulrich Würthwein

**Affiliations:** ^1^ Institut für Chemie und Biochemie Freie Universität Berlin Takustr. 3 14195 Berlin Germany; ^2^ Organisch‐Chemisches Institut and Center for Multiscale Theory and Computation (CMTC) Universität Münster Corrensstrasse 40 48149 Münster Germany

**Keywords:** 1,3‐Dipole, Carbene, Density functional theory, Diazoalkane, Electrocyclic reaction

## Abstract

An electrocyclic ring closure of bis‐1,3‐dipoles can afford six‐membered heterocycles. This 8π‐electron process was systematically analyzed by DFT calculations with 1,2‐bis(diazo)alkane derivatives as possible precursor compounds and 1,2,3,4‐tetrazines as products. The C_2_‐symmetry of the transition state of the parent system points to a conrotatory ring closing event. The subsequent (6–2–2) cycloreversions of these elusive nitrogen‐rich heterocycles to alkynes or nitriles and dinitrogen were also computationally investigated. The results show that the reactions are strongly dependent on the substitution pattern, but all are kinetically easily feasible delivering products of differing stability. The calculations can therefore provide important information for experimental endeavors to generate or even isolate so far unknown 1,2,3,4‐tetrazines. The feasibility of carbene or 1,2,3‐triazolyl‐substituted nitrene intermediates for the formation of alkynes is also discussed. The experimental evidence for the proposed processes is enclosed presenting literature known examples of the fragmentation reactions which can most convincingly be explained by the intermediacy of 1,2,3,4‐tetrazine derivatives. Furthermore, the electrocyclic ring closure reactions of five other types of bis‐1,3‐dipoles are calculated, demonstrating that this so far ignored 8π‐electrocyclization process can establish a new route to interestingly composed heterocyclic compounds.

## Graphical abstract

DFT calculations of the 8π‐electrocyclization of 1,2‐bis(diazo)alkanes and of other bis‐1,3‐dipoles reveal that this process can establish a so far ignored route to heterocycles such as 1,2,3,4‐tetrazines. Alternative reaction channels via carbenes or nitrenes leading to fragmentation products are discussed. The computations identified substitution patterns that could allow the characterization or isolation of the elusive 1,2,3,4‐tetrazines. The C_2_‐symmetric geometries of the transition states point to a conrotatory electrocyclic process.

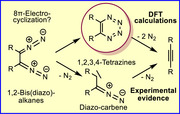

## Introduction

**Scheme 1:**
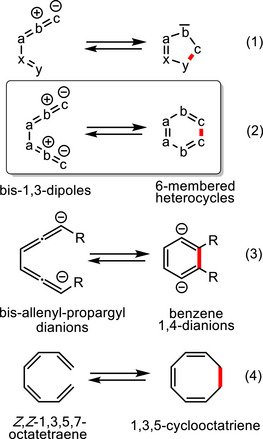
Electrocyclic reactions involving 6π‐electrons (Equation 1) or 8π‐electrons (Equations 2–4).

The Huisgen reaction of 1,3‐dipolar species of general structure “abc” with dipolarophiles “xy” provides five‐membered heterocycles and constitutes a well‐known and very flexible method in heterocyclic chemistry.^[^ ^1^, ^2^, ^3^, ^4^, ^5^ ^]^ If 1,3‐dipoles “abc” are directly connected to “xy” 1,5‐electrocyclizations to five‐membered heterocycles are possible (Scheme 1, Equation 1) and many examples of this 6π‐electron process or its reverse reaction are known.^[^ ^6^, ^7^ ^]^ In only a few cases, the 8π‐electron reaction of 1,3‐dipolar species bearing 1,3‐(hetero)dienyl substituents to seven‐membered heterocycles has been reported.^[^ ^8^, ^9^, ^10^, ^11^, ^12^, ^13^ ^]^ Triggered by the formation of 1,3,4‐thiadiazine derivatives found in an earlier study^[^ ^14^ ^]^ (see below) we raised the question whether an 8π‐electrocyclization of two directly connected 1,3‐dipoles can lead to six‐membered heterocycles (Equation 2).

In Equation 2 of Scheme 1 bis‐1,3‐dipoles with a symmetric arrangement of the centers “abc” are depicted. Of course, instead of allenyl‐propargyl type 1,3‐dipoles^[^ ^1^, ^2^, ^3^, ^4^, ^5^ ^]^ as presented, it is also possible to include allyl type 1,3‐dipoles into this scenario or to combine different 1,3‐dipoles in suitable arrangements. The cyclization of Equation 2 is electronically equivalent to the reaction of bis‐allenyl‐propargyl dianions to benzene 1,4‐dianions (Scheme 1, Equation 3), which is unfavorable due to the high concentration of negative charge in the product; to our best knowledge no examples of this reaction are known.^[^ ^15^ ^]^ However, if the 8π electrons are distributed to eight atoms such as in uncharged *Z*,*Z*‐1,3,5,7‐octatetraene systems the corresponding electrocyclic process to 1,3,5‐cyclooctatriene is smoothly possible (Scheme 1, Equation 4). By use of suitably substituted compounds classical experiments of Huisgen et al.^[^ ^16^, ^17^ ^]^ confirmed the validity of the Woodward‐Hoffmann rules which predicted the conrotatory ring closure.^[^ ^18^, ^19^ ^]^

**Scheme 2:**
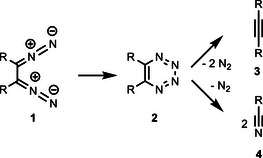
8π‐Electrocyclization of 1,2‐bis(diazo)alkanes **1** to 1,2,3,4‐tetrazines **2** and subsequent (6–2–2) cycloreversions to alkynes **3** or nitriles **4**.

Comparable stereochemical markers are not possible for the system described in Equation 2, nevertheless the principal question about the feasibility of this type of cyclization is of general interest since it may open new routes to interestingly composed heterocycles. To the best of our knowledge, this pathway to heterocyclic products was not generally discussed in the past. As a rare exception we found the review of Hendrickson on thermal pericyclic reactions published in 1974,^[^ ^20^ ^]^ which briefly mentions the reaction of 1,2‐bis(diazo)alkanes **1** to 1,2,3,4‐tetrazines **2** and the (6–2–2) cycloreversion of this product to provide alkyne **3** and dinitrogen; a footnote states that the alternative fragmentation into two molecules of nitriles **4** and dinitrogen is also feasible (Scheme 2).^[^ ^21^ ^]^ Whereas the conversion of 1,2‐bis(diazo)alkanes **1** to alkynes **3** may proceed also via alternative mechanistic pathways, the generation of nitriles **4** requires the intermediacy of 1,2,3,4‐tetrazines **2**.

Since a few experimental results support the processes depicted in Scheme 2 (see below) we concentrated our computational study on compounds **1** and **2** and their subsequent products. It is evident that nitrogen‐rich 1,2,3,4‐tetrazines **2** are highly unstable (energy rich) species and as a consequence the parent compound or simple compounds of this unique class of heterocycles are up to now unknown. Only specifically substituted derivatives or partially saturated compounds are mentioned in the literature.^[^ ^22^, ^23^, ^24^, ^25^, ^26^, ^27^, ^28^ ^]^ On the other hand, there are several theoretical studies focusing on the aromaticity of the parent compound in comparison with other compound with 6π electrons.^[^ ^29^, ^30^, ^31^, ^32^, ^33^ ^]^

## Results and Discussion

### Computational Details

Precursor compounds, transition states, possible intermediates and products will be discussed on the basis of the Gibbs free energy surface as investigated by a comprehensive quantum chemical study. DFT geometry optimizations were performed using the hybrid functional PBE1PBE/def2TZVP^[^ ^34^, ^35^, ^36^, ^37^, ^38^ ^]^ including Grimme dispersion GD3BJ^[^ ^39^, ^40^ ^]^ and the PCM solvent sphere of dichloromethane.^[^ ^41^ ^]^ For control and comparison hybrid functional wB97X‐D/def2TZVP‐optimizations^[^ ^42^ ^]^ were done for some species of series **a** (R = H). In the following section, we discuss differences in Gibbs free energies (Δ*G*_298_) (kcal mol^−1^) (see Supporting Information for details). Transition state searches were performed on the basis of stepwise bond elongations; for the N_2_‐splitting, van der Waals complexes were identified as intermediates or transition states. IRC‐calculations were used to identify starting materials and products of transition state searches.^[^ ^43^ ^]^ In this study only closed shell calculations were performed for the pericyclic reactions. For the dimerization of the diazomethyl radical CHN_2_ to 1,2‐bis(diazo)ethane CCSD(T)/cc‐pvtz‐geometry optimizations^[^ ^44^, ^45^, ^46^, ^47^, ^48^, ^49^ ^]^ were performed. All calculations were done using the Gaussian 16 package of programs.^[^ ^50^ ^]^

### Reactions of 1,2‐Bis(Diazo)Alkanes via 1,2,3,4‐Tetrazines

**Figure 1:**
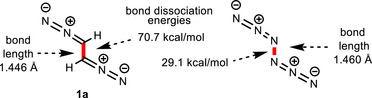
Comparison of lengths and dissociation energies and of the central bonds of **1a** and hexanitrogen^[^ ^51^, ^52^ ^]^ at the CCSD(T)/cc‐pVTZ level of theory.

**Scheme 3:**
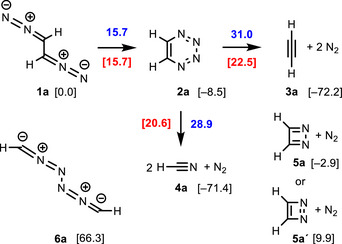
Electrocyclization of 1,2‐bis(diazo)ethane (**1a**) to 1,2,3,4‐tetrazine (**2a**) and fragmentations to ethyne (**3a**) or hydrogen cyanide (**4a**), energy of transition states **TS** and the resulting reaction barriers **RB** (Δ*G*_298_ in kcal mol^−1^).

For the parent compound 1,2‐bis(diazo)ethane (**1a**) a conformational analysis revealed a flat potential energy surface with a minimum for a dihedral angle of 172°. The orthogonal arrangement of **1a** is only 0.45 kcal mol^−1^ higher and the *cisoid* conformation 2.05 kcal mol^−1^ higher in energy [for details, including the conformational analysis of bis(nitrile imine) **6a**, see Supporting Information]. A computational comparison of **1a** with the closely related hexanitrogen, as recently characterized by Schreiner et al.,^[^ ^51^, ^52^ ^]^ is shown in Figure 1. The homolysis of **1a** to CHN_2_ radicals^[^ ^53^, ^54^, ^55^, ^56^, ^57^, ^58^, ^59^ ^]^ requires 70.7 kcal mol^−1^, indicating a stronger central bond compared with that of N_6_. The formation of diazomethyl radicals is much less favorable than the processes illustrated in Scheme 3 and hence their participation and that of other radical species is unlikely.

**Figure 2:**
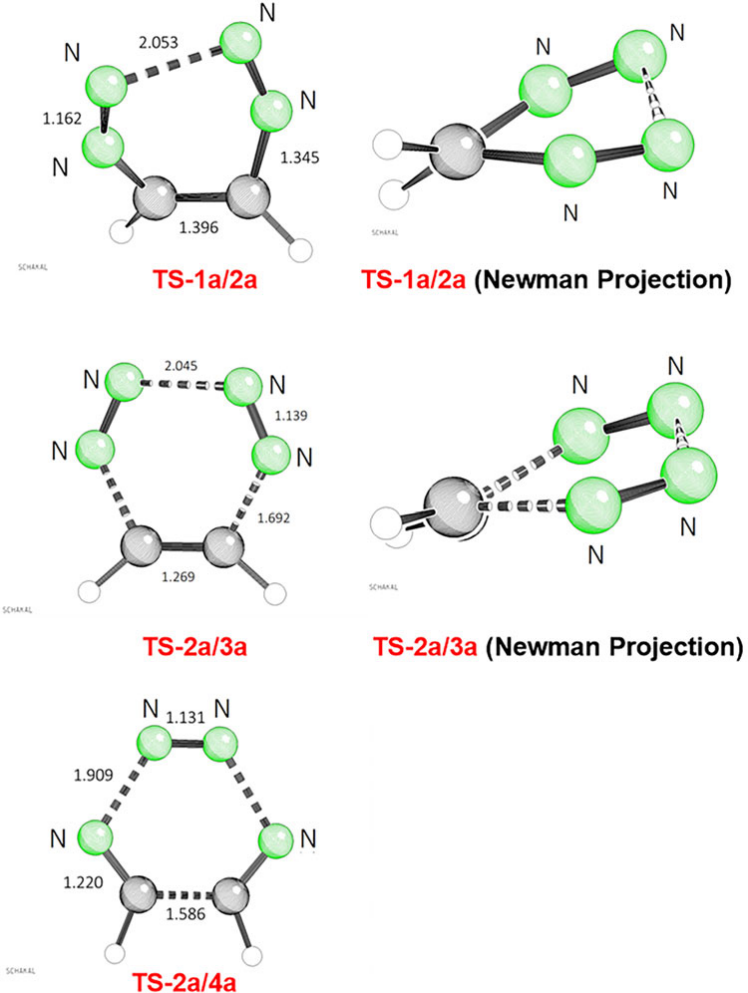
Transition states of the electrocyclic ring closure **TS‐1a/2a** and of fragmentation reactions **TS‐2a/3a** and **TS‐2a/4a** (bond lengths given in Å).

The electrocyclic reaction of 1,2‐bis(diazo)ethane (**1a**) to 1,2,3,4‐tetrazine (**2a**) is a moderately exergonic process (−8.5 kcal mol^−1^) and the reaction barrier **RB‐1a/2a** of 15.7 kcal mol^−1^ indicates that the reaction should be easily feasible at low temperatures (Scheme 3). The transition state **TS‐1a/2a** (Figure 2) shows twisted C_2_‐symmetric arrangement with dihedral angles of 39.4° (N─C─C─N) and −23.9° (N─N─N─N). The C_2_‐symmetry of this transition state points to a conrotatory ring closure event as postulated by the Woodward‐Hoffmann rules for a thermal 8π‐process.^[^ ^18^, ^19^ ^]^ The developing newly generated N‐N bond has a length of 2.053 Å, which corresponds to a bond length of 1.318 Å in product **2a**.

Higher reaction barriers of 22.5 and 20.6 kcal mol^−1^ were calculated for the two possible concerted (6–2–2) cycloreversions, which lead either to ethyne (**3a**) or to hydrogen cyanide (**4a**) (Scheme 3). As expected both processes are extremely exothermic (−72.2 and −71.4 kcal mol^−1^) due to the formation of dinitrogen. Surprisingly, the transition state of **TS‐2a/3a** is also moderately twisted with dihedral angles of 32.8° (N─C─C─N) and 14.9° (N─N─N─N).^[^ ^60^ ^]^ On the other hand, **TS‐2a/4a** show the expected C_2v_‐symmetric arrangement of centers as required for a process involving six π‐electrons (Figure 2).

With regard to alternative fragmentation reactions of **2a**, we also investigated the two 1,2‐diazacyclobutadiene isomers **5a** and **5a´**. Although energy is gained due to the release of dinitrogen, the formation of **5a** is only slightly exergonic, whereas that of **5a´** is even endergonic. This certainly reflects the antiaromatic character and the high ring strain of these species.^[^ ^61^ ^]^ However, an energetically suitable pathway from **2a** to **5a**/**5a´** could not be identified. To complete the H_2_C_2_N_4_ energy landscape we also included the bis(nitrile imine) **6a**, a constitutional isomer of **1a**, into the DFT calculations. With a Gibbs free energy of 66.3 kcal mol^−1^ this bis‐1,3‐dipole is tremendously unstable, probably due to the sequence of four contiguous nitrogen atoms and the weak N─N bonds. The occurrence of **6a** on the potential energy surface of **1a** and its formation involving a hitherto unknown type of [3,3]‐sigmatropic rearrangements can therefore be neglected.

In Table 1 the Gibbs free energies of a series of differently substituted 1,2‐bis(diazo)alkanes **1** and their conceivable products **2**, **3** and **4** (Scheme 2) as well as the involved transition states **TS** and the resulting reaction barriers **RB** are collected. For comparison, entry **a** represents the already discussed values of the parent system. Entries **b** to **d** summarize the calculations of 1,2‐bis(diazo)alkanes with cyclic backbones enforcing a fixed *cisoid* conformation of the two diazoalkane moieties. The found energy values for the formation of the corresponding 1,2,3,4‐tetrazines **2b** and **2c** bearing cyclopentane or cyclohexane substructures are rather similar to the parent compound **2a**. The higher transition state energy **TS‐1b/2b** (compared to **TS‐1a/2a** or **TS‐1c/2c**) is probably caused by the arrangement of the two diazoalkane moieties of **1b**, which deviates from optimal geometry due to the angle constraints. The alkyl substitution weakly stabilize the heterocycles by 8–10 kcal mol^−1^. As expected, the fragmentations of **2b** and **2c** to cycloalkynes **3b** and **3c**, respectively, are quite unfavorable due to the high ring strain of the products. For the fragmentation of compound **2b** to cyclopentyne (**3b**) no transition state could be localized (see discussion below), whereas for the transformation of **2c** into cyclohexyne (**3c**) a reaction barrier **RB** of 36.3 kcal mol^−1^ was calculated. The resulting cycloalkynes have free Gibbs energies of −15.6 and −41.9 kcal mol^−1^, respectively, clearly reflecting their high strain. On the other hand, the alternative fragmentations under cleavage of the C─C bond of the 1,2,3,4‐tetrazines providing the dinitriles **4b** and **4c** are highly exergonic since no strained products are formed. The reaction barriers **RB** leading to these compounds are moderate (23.2 and 35.3 kcal mol^−1^).

**Table 1:** Gibbs free energies of 1,2‐bis(diazo)alkanes **1**, 1,2,3,4‐tetrazine derivatives **2**, possible fragmentation products **3** or **4** and the respective transition states **TS** and the resulting reaction barriers **RB** (see Scheme 2 for numbering of compounds; Δ*G*_298_ in kcal mol^−1^ including the solvent sphere of dichloromethane).

Entry	R, R	1,2‐Bis(diazo)‐alkane **1**	**TS‐1/2**	= **RB‐1/2**	1,2,3,4‐Tetrazine **2**	**TS‐2/3**	**RB‐2/3**	Alkyne **3**	**TS‐2/4**	**RB‐2/4**	Nitrile **4**
**a**	H	0.0	15.7	15.7	−8.5	22.5	31.0	−72.2	20.6	29.1	−71.4
**b**	R─R (CH_2_)_3_	0.0	20.3	20.3	−18.1	^a)^	–	−15.6	5.1	23.2	−84.6
**c**	R─R (CH_2_)_4_	0.0	13.0	13.0	−20.1	16.2	36.3	−41.9	15.2	35.3	−80.0
**d**	R─R (CH)_4_	0.0	9.3	9.3	−26.6	2.0	28.6	−49.1	24.0	50.6	−68.9
**e**	Ph	0.0	11.8	11.8	−7.5	18.8	26.3	−78.2	28.9	36.4	−78.4
**f**	CN	0.0	20.7	20.7	8.8	25.8	17.0	−69.0	43.8	35.0	−47.6
**g**	NMe_2_	0.0	11.9	11.9	─36.6	^b)^	–	─82.7	^c)^	–	─94.2

^a)^ Search for **TS‐2b/3b** leads to carbene **7b** and N_2_ (see discussion below and Scheme 5).

^b)^ Search for **TS‐2g/3g** leads to carbene **7** **g** and N_2_ (see Scheme 5).

^c)^ Search for **TS‐2g/4g** leads to a transition state leading to a nucelophilic carben (see Supporting Information).

**Scheme 4:**
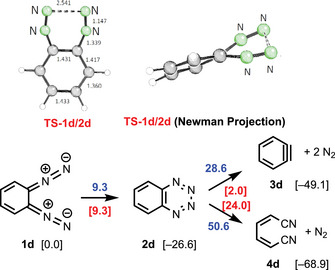
Reaction of 5,6‐bis(diazo)cyclohexa‐1,3‐diene (**1d**) to benzo[e][1,2,3,4]tetrazine (**2d**), its fragmentation into **3d** or **4d**, the respective transition states **TS**, the resulting reaction barriers **RB** (Δ*G*_298_ in kcal mol^−1^) and twisted **TS‐1d/2d** (dihedral angles C─N─N─C −39.6° and N─N─N─N −14.5°).

Entry **d** presents the data obtained for the conjugated system, which is separately depicted for clarity in Scheme 4. The aromatic benzo[e][1,2,3,4]tetrazine (**2d**) is formed via a barrier of only 9.3 kcal mol^−1^ and is 26.6 kcal mol^−1^ more stable than the proposed precursor 5,6‐bis(diazo)cyclohexa‐1,3‐diene (**1d**) which has a cross‐conjugated *ortho*‐quinoid structure. The C_2_‐symmetric transition state **TS‐1d/2d** is twisted although the benzo‐back bone should disfavor this geometry; this can again be taken as evidence for a conrotatory ring closure.

The (6–2–2) cycloreversion of **2d** into benzyne (**3d**) has to pass a reaction barrier **RB‐2d/3d** of 28.6 kcal mol^−1^ and is exergonic. As expected, the fragmentation of **2d** to unsaturated dinitrile **4d** is even more exergonic, however, the reaction barrier **RB‐2d/4d** for this process was calculated to be 50.6 kcal mol^−1^, which indicates that this fragmentation is kinetically unfavorable. The loss of aromaticity may be responsible for this effect. Comparison of the Gibbs free energies of cyclohexyne (**3c**) and of benzyne (**3d**) reflect the better stabilization of the “triple bond” in the unsaturated compound. The values of entry **d** and Scheme 4 indicate that it should be possible to generate and characterize the bicyclic benzo[e][1,2,3,4]tetrazine (**2d**) at temperatures slightly below room temperature. Furthermore, the data indicate that **1d** may be a suitable precursor for benzyne (**3d**).^[^ ^62^, ^63^, ^64^ ^]^

The substitution by two phenyl groups (entry **e** of Table 1) does not strongly change the energy values compared to those of the parent system (entry **a**). The most evident differences are the slight lowering of the reaction barrier **RB‐1e/2e** to 11.8 kcal mol^−1^ for the electrocyclic reaction of **1e** to 5,6‐diphenyl‐1,2,3,4‐tetrazine (**2e**) and of **RB‐2e/3e** to 26.3 kcal mol^−1^ for the subsequent fragmentation of **2e** to 1,2‐diphenylethyne (**3e**). On the other hand, the alternative (6–2–2) cycloreversion to benzonitrile (**4e**) is kinetically and thermodynamically less favorable. The data of entry **e** indicate that the electrocyclic reactions of diaryl‐substituted 1,2‐bis(diazo)alkanes should be particularly fast.

The introduction of strong electron‐withdrawing or electron‐donating substituents caused significant changes as seen by comparing entries **f** (R═CN) and **g** (R═NMe_2_) with entry **a** (Table 1). The electrocyclic ring closure of 1,2‐bis(diazo)alkane **1f** to 5,6‐dicyano‐1,2,3,4‐tetrazine (**2f**) is even endergonic by 8.8 kcal mol^−1^ and the corresponding transition state has an energy of 20.7 kcal mol^−1^. The electron‐withdrawing substituents apparently destabilize 1,2,3,4‐tetrazine **2f**. Therefore, it should be possible to characterize 1,2‐bis(diazo)alkane **1f** under suitable conditions. On the other hand, the formation of 5,6‐bis(dimethylamino)‐1,2,3,4‐tetrazine (**2g**) from precursor **1g** is strongly exergonic by −36.6 kcal mol^−1^ and the reaction barrier for this process is rather low (11.9 kcal). As expected, the subsequent fragmentations of the 1,2,3,4‐tetrazines **2f** and **2g** to alkynes or nitriles are exergonic. The reaction barriers calculated for the dicyano compound **2f** are low for the (6–2–2) cycloreversion to 1,2‐dicyanoethyne (**3f**) (17.0 kcal mol^−1^) and moderate for the alternative fragmentation leading to two molecules of oxalonitrile (**4f**) and nitrogen (35.0 kcal mol^−1^). The two possible (6–2–2) cycloreversions of donor‐substituted 1,2,3,4‐tetrazine **2g** should be very exergonic, which reflects the high energy level of the reference compound **1g**. However, the DTF calculations did not provide transition states leading directly to **3g** or to **4g**. The very strong electron‐donating ability of the dimethylamino group leads to stabilized carbene or nitrene intermediates which are discussed in detail in the next paragraph.

### Alternative Fragmentations via Sextet Intermediates

**Scheme 5:**
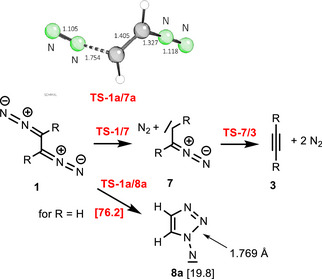
Reactions of 1,2‐bis(diazo)alkanes **1** to alkynes **3** via diazoalkyl‐substituted carbenes **7** or 1,2,3‐triazolyl‐substituted nitrenes **8** (Δ*G*_298_ in kcal mol^−1^) and transition state **TS‐1a/7a**.

For the conversions of 1,2‐bis(diazo)alkanes **1** into nitriles **4**, 1,2,3,4‐tetrazines **2** seem to be unavoidable intermediates, which is not the case for the fragmentation of **1** to alkynes **3**. Here, mechanisms via carbenes **7** or nitrene intermediates **8** are conceivable as depicted in Scheme 5. The Gibbs free energies of these species, of the transition states and of alkynes **3** are collected in Table 2. The elimination of dinitrogen leads to van der Waals interactions with the resulting species and therefore the energies were calculated without and with these interactions. Due to entropic effects, the Gibbs free energy values under inclusion of van der Waals interactions are less negative by 4–5 kcal mol^−1^ in most cases.

**Table 2:** Gibbs free energies of carbenes **7** and alkynes **3** [relative to 1,2‐bis(diazo)alkanes **1**] and transition states **TS‐1/7** and **TS‐7/3**; values considering van der Waal interactions with dinitrogen^a)^ (Δ*G*_298_ in kcal mol^−1^; see Scheme 5 for numbering of compounds).

Entry	R, R	**TS‐1/7**	**7**	**TS‐7/3**	**3**
**a**	H	19.9	−1.9	−2.6	−65.8
**b**	R─R (CH_2_)_3_	20.7	−5.9	7.4	−6.5
**c**	R─R (CH_2_)_4_	18.8	−4.7	1.0	−33.7
**d**	R─R (CH)_4_	b)	−17.9	−14.4	−42.0
**e**	Ph	22.7	0.3	0.4	−70.3
**f**	CN	18.3	−5.9	−4.8	−63.8
**g**	NMe_2_	5.8	−29.8	−22.5	−74.1

^a)^ Values without van der Waals interactions are listed in the Supporting Information.

^b)^ Search for **TS‐1d/7d** leads to minimum for alkyne **3d** and N_2_.

For parent system **a**, the corresponding diazomethyl‐substituted carbene **7a** is formed via a barrier of 19.9 kcal mol^−1^ in a moderately exergonic process (−1.9 kcal mol^−1^). These values should be compared with entry **a** of Table 1 which shows a slightly lower transition state energy (15.7 kcal mol^−1^) for the electrocyclic reaction. The 1,2,3,4‐tetrazine **2a** is more stable than carbene **7a**, nevertheless the two pathways may compete. The transitions state **TS‐1a/7a** shows the expected *transoid* arrangement and the dissociating C─N bond has a length of 1.754 Å. The subsequent loss of the second molecule dinitrogen generating ethyne (**3a**) is a process essentially without reaction barrier (transition state energy −2.6 kcal mol^−1^). The overall pathway **1**→**7**→**3** can be compared with the Eschenmoser fragmentation, which involves dinitrogen and a ketone as leaving molecules for the formation of an alkyne.^[^ ^65^ ^]^

The Gibbs free energies of **TS‐1/7** for carbene formation are similar in entries **a** to **f** (ca. 20 kcal mol^−1^) and the processes are exergonic; only carbene intermediate **7d**, derived from the *ortho*‐quinoid 1,2‐bis(diazo)alkane **1d**, deviates by higher stability. All carbenes **7b**‐**7g** rapidly loose the second dinitrogen molecule giving the corresponding alkynes **3b**‐**3g**. According to the values of Table 2, the fragmentations via **7a**–**7f** have to be considered as alternative to the 1,2,3,4‐tetrazine pathway; only for the phenyl‐substituted species **TS‐1e/7e** is significantly higher than **TS‐1e/2e** (difference of ca. 10 kcal mol^−1^). Entry **g** with species bearing dimethylamino substituents shall be discussed separately below.

An alternative 1,5‐electrocyclization of **1** hypothetically leads to 1,2,3‐triazolyl‐substituted nitrenes **8** (Scheme 5). For the parent system **a**, the transition state **TS‐1a/8a** of this process is extremely high (76.2 kcal mol^−1^) and the Gibbs free energy of **8a** amounts to 19.8 kcal mol^−1^; intermediate **8a** has a remarkably long newly formed N‐N bond (1.769 Å). The reaction barrier of the very exergonic hypothetical fragmentation of **8a** into ethyne (**3a**) and dinitrogen is only 21.0 kcal mol^−1^ high. However, since the Gibbs free energy of **TS‐1a/8a** is extremely high, it is unlikely that intermediate **8a** is involved in the formation of **3a**.^[^ ^66^ ^]^

**Scheme 6:**
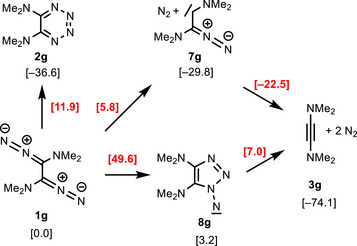
Reactions of bis(dimethylamino)‐substituted 1,2‐bis(diazo)ethane **1g** to alkyne **3g** via carbene **7g** (pathway 1) or nitrene **8g** (pathway 2) (Δ*G*_298_ in kcal mol^−1^).

The very strongly electron‐donating dimethylamino groups of species collected in entry **g** dramatically influence the energy values of all species (Scheme 6). The 1,2,3,4‐tetrazine **2** **g** is generated via a transition state energy of 11.9 kcal mol^−1^, however, a lower barrier of 5.8 kcal mol^−1^ leads to the fairly stable carbene/nitrogen complex **7** **g** (−29.8 kcal mol^−1^). These values reflect the energetically unfavorable situation of precursor **1** **g** where two strongly electron‐donating dimethylamino groups push electron density into the electron‐rich 1,2‐bis(diazo)ethane moiety.^[^ ^67^, ^68^ ^]^ In contrast, carbene **7g** is well stabilized by the attached dimethylamino group. It loses the second dinitrogen molecule without barrier to give bis(dimethylamino)ethyne (**3g**).^[^ ^69^, ^70^ ^]^ The computations also show that the hypothetical formation of 1,2,3‐triazolyl‐substituted nitrene **8g** has a much lower reaction barrier and nitrene **8g** is only 3.2 kcal mol^−1^ less stable than its precursor **1g**; apparently, the electron‐rich heterocyclic substituent strongly contributes to the stabilization of this sextet species. The still high transition state energy of 49.6 kcal mol^−1^ shows that formation of **3g** via **8g** is unlikely.^[^ ^71^ ^]^

### Summarizing Discussion

The collected data reveal that the proposed 8π‐electrocyclic reaction of 1,2‐bis(diazo)alkanes **1** to 1,2,3,4‐tetrazine derivatives **2** is energetically easily feasible. Not surprisingly, the experimentally observed fragmentation products, the alkynes **3** and/or nitriles **4**, are thermodynamically much more stable than **2**. However, relatively high reaction barriers should decrease the rate of these (6–2–2) cycloreversions and hence “protect” the 1,2,3,4‐tetrazines **2** from fragmentation. Whereas the involvement of nitrene intermediates **8** is very unlikely, the generation of diazomethyl‐substituted carbene intermediates **7** opens an attractive alternative reaction channel for 1,2‐bis(diazo)alkanes **1** to alkynes **3** (but not to nitriles **4**). In Table 3 the most important data of Tables 1 and 2 are combined to allow an easier comparison of the energies of **TS‐1/2** and **TS‐1/7** and of the resulting products **2** and **7**. This inspection should facilitate an estimation which substitution pattern may allow the characterization or even isolation of so far unknown simple 1,2,3,4‐tetrazine derivatives **2**. The data of entries **a** (R = H), **c** [R = (CH_2_)_4_] and **e** (R = Ph) reveal that **TS‐1/2** are lower than **TS‐1/7** and that 1,2,3,4‐tetrazines **2a** and **2e** are also more stable than the corresponding carbenes.

**Table 3:** Comparison of Δ*G*_298_ (kcal mol^−1^) of **TS‐1/2** and **TS‐1/7** and of the resulting products 1,2,3,4‐tetrazines **2** and carbenes **7**.

Entry	R, R	**TS‐1/2**	**TS‐1/7**	**2**	**7** ^a)^
**a**	H, H	15.7	19.9	−8.5	−1.9
**b**	(CH_2_)_3_	20.3	20.7	−18.1	−5.9
**c**	(CH_2_)_4_	13.0	18.8	−20.1	−4.7
**d**	(CH)_4_	9.3	^b)^	−26.6	−17.9
**e**	Ph, Ph	11.8	22.7	−7.5	0.3
**f**	CN, CN	20.7	18.3	8.8	−5.9
**g**	NMe_2_, NMe_2_	11.9	5.8	−36.6	−29.8

^a)^ Values with consideration of van der Waals interactions.

^b)^ Search for **TS‐1d/7d** leads to a minimum for alkyne **3d** and N_2_ (see Table 2).

**Scheme 7:**
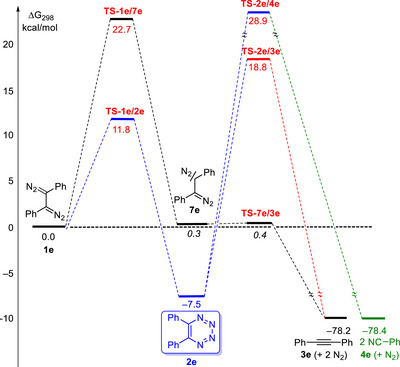
Energy profile of the reactions of 1,2‐bis(diazo)alkane **1e** to 1,2,3,4‐tetrazine **2e** or carbene intermediate **7e** and subsequent fragmentation products **3e** or **4e**; for **7e** and **TS‐7e/3e** the values including van der Waals interactions were considered (Δ*G*_298_ in kcal mol^−1^).

As example, the energy profile of diphenyl‐substituted compounds and intermediates of entry **e** is depicted in Scheme 7. The 5,6‐diphenyl‐1,2,3,4‐tetrazine (**2e**) is formed via a relatively low reaction barrier whereas the generation of the diazomethyl‐substituted carbene **7e** requires ca. 11 kcal mol more. Furthermore, the heterocycle **2e** is 7.8 kcal mol more stable than **7e**. Whereas the carbene **7e** fragments to alkyne **3e** without reaction barrier, the heterocycle **2e** is “protected” by high reaction barriers of more than 25 kcal mol from fragmentation into **3e** or **4e**. Therefore, diaryl‐substituted 1,2‐bis(diazo)alkanes should be suitable substrates for the generation of 1,2,3,4‐tetrazines. The aryl groups may also allow a further fine‐tuning of the kinetic and thermodynamic parameters; the examples of series **f** and **g** indicate that electron‐withdrawing and electron‐donating substituents directly bound to the 1,2‐bis(diazo)alkene core have a strong effect on the obtained data. As mentioned already above when discussing Scheme 4, compounds such as 5,6‐bis(diazo)cyclohexa‐1,3‐diene (**1d**) could also be a suitable precursor for the synthesis of identifiable 1,2,3,4‐tetrazines; again additional substituents in the cyclohexadiene backbone may contribute to the feasibility the process. Our data also reveal, that even the characterization of the parent compound 1,2,3,4‐tetrazine (**2a**) may be possible if the generation of 1,2‐bis(diazo)alkane (**1a**) succeeds under sufficiently mild conditions.

### Experimental Evidence

**Scheme 8:**
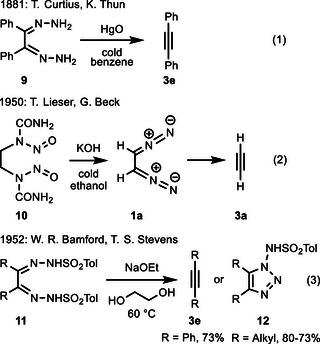
Literature reported transformations involving 1,2‐bis(diazo)alkanes as intermediates of the formation of alkynes **3**.

Before concluding our computational analysis, the experimental evidence for the discussed electrocyclic reactions and/or fragmentation reactions should be summarized. It has been reported as early as 1881 that the oxidation of bishydrazones delivers alkynes (Scheme 8, Equation 1). Curtius and Thun studied the reaction of benzil bishydrazone **9** with mercury oxide providing tolane **3e** apparently in fair amounts; a yield is not given for this experiment, however, no side products were mentioned.^[^ ^72^ ^]^ Employing various oxidation agents, this method was frequently used to prepare alkynes, including (strained) medium‐sized ring systems, but often considerable amounts of other products were formed.^[^ ^73^ ^]^ The mechanisms reported suggest that diazoalkanes are formed, but the intermediacy of 1,2,3,4‐tetrazines is essentially not discussed. A second standard method for the synthesis of diazoalkanes was employed by Lieser and Beck (Equation 2).^[^ ^74^ ^]^ The authors reported that 1,2‐bis(diazo)ethane (**1a**) and finally ethyne (**3a**) are formed by treatment of bis‐(*N*‐nitroso) urea derivative **10** with potassium hydroxide, however no detailed product isolation and characterization are described.^[^ ^75^ ^]^ An important extension of available methods for generation of diazoalkanes from sulfonylhydrazones is owed to Bamford and Stevens. In one of their seminal reports, they also present examples of bis(sulfonylhydrazones) **11**.^[^ ^76^ ^]^ The benzil bis(sulfonylhydrazone) furnished a high yield of tolane **3e**, whereas precursor compounds with alkyl substituents did not afford the corresponding alkynes but the sulfonamido‐substituted 1,2,3‐triazole derivatives **12** (Equation 3).^[^ ^77^, ^78^, ^79^, ^80^ ^]^ These products are probably formed by a 1,5‐electrocyclization of the intermediate mono‐diazoalkane moiety with the remaining hydrazone C═N double bond.^[^ ^81^ ^]^

**Scheme 9:**
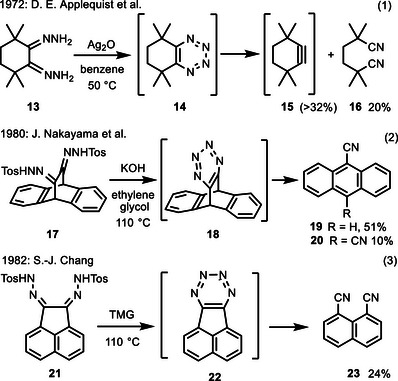
Literature reported transformations involving 1,2‐bis(diazo)alkanes as likely intermediates leading to (di)nitriles (TMG = tetramethylguanidine).

A few examples of the alternative fragmentation of 1,2,3,4‐tetrazine intermediates into dinitriles are also described in the literature (Scheme 9). All systems studied use precursor compounds which disfavor the formation of strained cycloalkynes. The oxidation of bishydrazone **13** provided dinitrile **16** in low yield; in addition, a Diels‐Alder adduct of cyclohexyne derivative **15** was isolated (Equation 1).^[^ ^82^ ^]^ Later, two publications reported the base‐promoted transformation of bis(tosylhydrazones) into 1,2‐bis(diazo)alkanes, their subsequent cyclization to 1,2,3,4‐tetrazine intermediates and (6–2–2) cycloreversions to the corresponding nitriles. Starting from compound **17** (Equation 2), the major product was anthracene‐9‐carbonitrile (**19**) formed by hydrogen cyanide elimination from the primary dinitrile; as minor component dinitrile **20** was formed by dehydrogenation.^[^ ^83^ ^]^ Finally, the acenaphthequinone derived bis(tosylhydrazone) **21** was converted into naphthalene‐1,8‐dicarbonitrile (**23**).^[^ ^84^ ^]^

The examples presented in Schemes 8 and 9 reveal that the proposed electrocyclic reaction of 1,2‐bis(diazo)alkanes has occasionally been observed, but the mechanistic analyses of the involved processes are rather lean in these reports. Whereas the generation of alkynes may also proceed via the discussed carbene pathway, the intermediacy of 1,2,3,4‐tetrazines is a compulsory consequence of the dinitrile formation.

### Electrocyclic Reactions of Other Bis‐1,3‐Dipoles

**Scheme 10:**
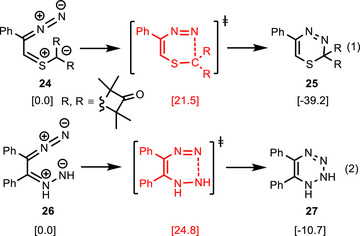
Electrocyclizations of hybrid bis‐1,3‐dipoles **24** and **26**, respectively, leading to 1,3,4‐thiadiazine derivative **25** or to 1,2‐dihydro‐1,2,3,4‐tetrazine derivative **27** (Δ*G*_298_ in kcal mol^−1^).

As briefly mentioned in the introduction, the impulse to investigate the electrocyclic ring closure of bis‐1,3‐dipoles arose from the isolation a 1,3,4‐thiadiazine derivative. Compound **25** was formed by reaction of an azoalkene with a thioketone^[^ ^14^ ^]^ and DFT calculations revealed that an electrocyclization of an intermediate hybride bis‐1,3‐dipole **24**, incorporating diazoalkane and thiocarbonyl ylide moieties (Scheme 10, Equation 1), to heterocycle **25** is a kinetically and thermodynamically feasible process. However, the calculations also indicated that the initially assumed [4 + 2]‐cycloaddition and a subsequent elimination step are energetically even more favorable and that **24** is most probable not involved as intermediate.

The hypothesized intermediate **24** contains a thiocarbonyl ylide moiety, which does not belong to the standard 1,3‐dipoles “abc” consisting of core atoms from the second period of the periodic table.^[^ ^1^, ^2^, ^3^, ^4^, ^5^ ^]^ Therefore, a hybrid bis‐1,3‐dipole combining an allenyl‐propargyl type 1,3‐dipole (diazoalkane) with an allyl type 1,3‐dipole (azomethine imine) was included into our computational analysis. The electrocyclic ring closure of **26** to **27** has a rather low barrier of 24.8 kcal mol^−1^ and it is moderately exergonic (Equation 2). The two examples of Scheme 10 demonstrate that the investigated electrocyclic ring closure reactions are energetically feasible also with allyl type 1,3‐dipoles and that they can lead to interestingly composed heterocycles. Of course, it is a synthetic challenge to generate these bis‐1,3‐dipoles by suitable methods.

**Scheme 11:**
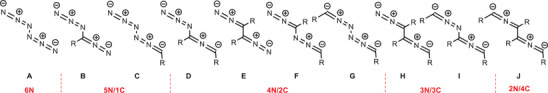
Ten allenyl‐propagyl type bis‐1,3 dipoles **A**–**J** obtained by combination of azide, diazoalkane, nitrile imine and nitrile ylide moieties.

Since the preceding computations dealt with diazoalkanes only, we wanted to compare these electrocyclizations with those of other allenyl‐propargyl type bis‐1,3‐dipoles. The series of possible systems (arranged by decreasing numbers of nitrogen atoms) starts with hexanitrogen **A**, which has recently been generated and characterized by Schreiner et al.,^[^ ^51^, ^52^ ^]^ and ends with bis(nitrile ylides) **J** (Scheme 11).

**Scheme 12:**
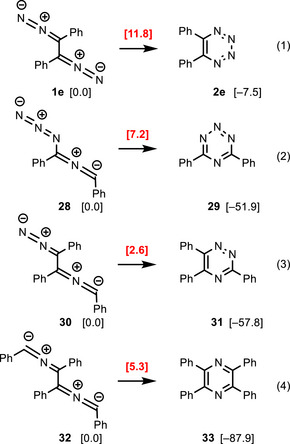
Electrocyclizations of hybrid bis‐1,3‐dipole **28** and **30** to heterocycles **29** and **31** and of bis(nitrile ylide) **32** to 2,3,5,6‐tetraphenylpyrazine (**33**) (Δ*G*_298_ in kcal mol^−1^).

We restricted our calculations to three synthetically practicable phenyl‐substituted bis‐1,3‐dipoles, namely to azido‐substituted nitrile imine **28** (the constitutional isomer of 1,2‐bis(diazo)alkane **1e**), the hybrid system **30**, and the bis(nitrile ylide) **32** (Scheme 12). Other possible bis‐1,3‐dipoles were not included because their high content of nitrogen atoms should lead to highly unstable heterocycles.^[^ ^85^ ^]^ Comparison of the already discussed transformation of **1e** to **2e** (Equation 1) with that of its isomers **28** to **29** (Equation 2) is quite striking. The barrier for this electrocyclic reaction is very low (7.2 kcal mol^−1^), but the high stability of **29** is particularly remarkable. The “isolation” of one of the four nitrogen atoms in the heterocyclic product **29** is probably responsible for this drastic effect. It should be noted, that the first monocyclic 1,2,3,5‐tetrazine derivatives were only very recently prepared and synthetically explored.^[^ ^86^, ^87^ ^]^ The electrocyclic reaction of Equation 2 may show an alternative route to prepare these elusive heterocycles.

The triphenyl‐substituted hybrid bis‐1,3‐dipole **30** (consisting of diazoalkane and nitrile ylide moieties) undergoes the cyclization to 1,2,4‐triazine derivative **31** with a very low barrier of 2.6 kcal mol^−1^ in a strongly exergonic fashion (−57.8 kcal mol^−1^) (Scheme 12, Equation 3). Thus, this reaction should be synthetically feasible. However, the fragmentation of **30** or **31** into tolane, benzonitrile and dinitrogen is even more exergonic (−60.7 kcal mol^−1^). We did not calculate the transition state of this process and hence it is not evident which reaction is kinetically favored. Similarly, the tetraphenyl‐substituted bis(nitrile ylide) **32** cyclizes to pyrazine derivative **33** with a low barrier of 5.3 kcal mol^−1^ and in a highly exergonic fashion of −87.9 kcal mol^−1^ (Equation 4). In this case, the alternative fragmentation of **32** into tolane **3e** and two benzonitrile molecules is less exergonic (−43.4 kcal mol^−1^). The energy balances of Equations 1 to 4 confirm the intuitively expected sequence: heterocyclic products with decreasing number of incorporated nitrogen atoms are increasingly more stable than their precursor compounds. Furthermore, the fragmentation into alkynes becomes thermodynamically less favorable if only one or no dinitrogen molecule is released. The electrocyclic reactions of hybrid bis‐1,3‐dipoles such as **30** or of bis(nitrile ylides) like **32** may therefore establish new routes to 1,2,4‐triazine or pyrazine derivatives.

**Scheme 13:**
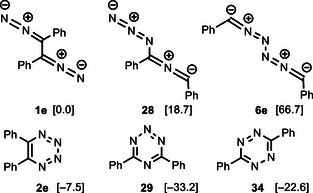
Comparison of three diphenyl‐substituted bis‐1,3‐dipoles **1e**, **28**, and **6e**, and three isomeric tetrazine derivatives **2e**, **29**, and **34** (Δ*G*_298_ in kcal mol^−1^).

Since all data are available, it is interesting to compare the Gibbs free energies of the three isomeric diphenyl‐substituted bis‐1,3‐dipoles **1e**, **28** and **6e** as well as their possible electrocyclization products **2e** and **29** (Scheme 13). The arrangement of nitrogen atoms is energetically most favorable in 1,2‐bis(diazo)alkane **1e** whereas the azido(nitrile imine) hybrid **28** is 18.7 kcal mol^−1^ less stable and bis(nitrile imine) **6e** is by far the least favorable isomer (66.7 kcal mol^−1^). Comparison of the two isomeric heterocycles **2e** and **29** confirms the above mentioned thermodynamic preference of the 1,2,3,5‐tetrazine over the 1,2,3,4‐tetrazine arrangement. Although the formation of 1,2,4,5‐tetrazines^[^ ^88^ ^]^ is not possible starting from bis‐1,3‐dipoles, compound **34** was included into this comparison. Interestingly, constitutional isomer **34** is less stable than **29**, but more stable than 1,2,3,4‐tetrazine **2e**. For the three unsubstituted tetrazine isomers the same sequence of thermodynamic stabilities was calculated.^[^ ^29^, ^30^, ^31^, ^32^, ^33^ ^]^

## Conclusions

Our comprehensive computational study clearly shows that so far ignored 8π‐electrocyclizations^[^ ^89^, ^90^ ^]^ of bis‐1,3‐dipoles can establish new routes to heterocyclic compounds. Most of the DFT calculations were done for 1,2‐bis(diazo)alkanes **1** and substitution patterns were identified, which may allow the characterization or even isolation of the resulting elusive 1,2,3,4‐tetrazine derivatives **2**. The alternative reaction channels of **1** via diazoalkyl‐substituted carbenes **7** or nitrenes **8** are calculated and the probability of their participation is discussed. Literature reports provide a few experimental examples and confirm that 1,2‐bis(diazo)alkanes **1** undergo facile fragmentations in dinitrogen and alkynes **3** or nitriles **4**, in general, without mentioning the corresponding 1,2,3,4‐tetrazines **2** as intermediates. For the proof of **2** as intermediates, the critical point in future studies will therefore be the generation of the corresponding diazoalkane moieties under reasonably mild conditions to slow down these subsequent reactions. Approved methods such as hydrazone oxidations^[^ ^91^, ^92^ ^]^ and Bamford–Stevens reactions^[^ ^93^, ^94^, ^95^ ^]^ or newly established methods, such as photolysis of 1,3,4‐oxadiazoline derivatives^[^ ^96^ ^]^ may work at sufficiently low temperatures to fulfill this prerequisite.

Our computations indicate that 1,2‐bis(diazo)alkanes **1d** and **1e** and hence the resulting 1,2,3,4‐tetrazines **2d** and **2e** are good candidates to be generated under mild conditions avoiding fast fragmentations of these energy‐rich heterocycles. These aryl‐substituted or benzannulated systems also allow further electronic fine‐tuning by substituents. On the other hand, strongly electron‐withdrawing groups may allow the characterization of 1,2‐bis(diazo)alkanes such as **1f**.

The explorative computations with other bis‐1,3‐dipoles, either of symmetrical systems or of hybrid systems, reveal that the proposed 8π‐cyclizations can also lead to other nitrogen‐containing heterocyclic compounds. Not surprisingly, the feasibility of these reactions and the stability of the resulting heterocycles increase by decreasing number of participating nitrogen atoms. For instance, the preparation of 1,2,4‐triazines such as **31** should be possible starting from a hybrid bis‐1,3‐dipole **30** consisting of diazoalkane and nitrile ylide moieties. From bis(nitrile ylides),^[^ ^97^ ^]^ pyrazine derivatives may be accessible; the computed example of tetraphenyl‐substituted compounds **32** and **33** confirms this claim. Even so far rare 1,2,3,5‐tetrazines such as compound **29** should be accessible by the 8π‐cyclization of hybrid bis‐1,3‐dipole **28**. In all these processes, the fragmentation into dinitrogen, alkynes and nitriles will impair the stability of the heterocycles, however, our computational results show that this process is less favorable if less dinitrogen molecules can potentially be eliminated. According to the Woodward–Hoffmann rules 8π‐electrocyclic reactions should proceed in conrotatory fashion and the geometries of the calculated transition states **TS‐1/2** apparently confirm this prediction. This may be experimentally proved by studying suitably substituted allyl type bis‐1,3‐dipoles. For preparative applications of the proposed 8π‐electrocyclization the synthesis of the required bis‐1,3‐dipoles remains the major challenge, however, our study should motivate synthetic chemists to develop suitable methods and to study exciting and energy‐rich compounds such as 1,2,3,4‐tetrazines **2**.

## Conflict of Interests

The authors declare no conflict of interest.

## Supporting information



Supporting Information

## Data Availability

The data that support the findings of this study are available in the supplementary material of this article.
