# Supplementary material for: Computational Study of Ignored Pericyclic Reactions: Rearrangements of 1,2‐Bis(Diazo)Alkanes to 1,2,3,4‐Tetrazines and Subsequent Fragmentations
Source: Angew Chem Int Ed Engl. 2025 Sep 16;64(45):e202514598. doi: 10.1002/anie.202514598 (PMC12582011; doi:10.1002/anie.202514598)
Supplement: Supplementary file 1 — Supporting Information [file ANIE-64-e202514598-s001.docx]

**Supplementary Information**

Computational Study of Ignored Pericyclic Reactions: Rearrangements of 1,2-Bis(diazo)alkanes to 1,2,3,4-Tetrazines and Subsequent Fragmentations

Hans-Ulrich Reissig*,^[a]^ Ernst-Ulrich Würthwein*^[b]^

Dedicated to the memory of Prof. Dr. Jürgen Fabian (1936–2023) and Prof. Dr. Klaus Banert (1955–2020)

[a] *Prof. Dr. H.-U. Reissig
Institut für Chemie und Biochemie
Freie Universität Berlin
Takustr. 3, 14195 Berlin, Germany
E-mail:* [*hreissig@zedat.fu-berlin.de*](mailto:hreissig@zedat.fu-berlin.de)
[*http://www.bcp.fu-berlin.de/en/chemie/chemie/forschung/OrgChem/reissig/index.html*](http://www.bcp.fu-berlin.de/en/chemie/chemie/forschung/OrgChem/reissig/index.html)

[b] *Prof. Dr. E.-U. Würthwein
Organisch-Chemisches Institut and Center for Multiscale Theory and Computation (CMTC)
Universität Münster
Corrensstrasse 40, 48149 Münster, Germany
E-mail:* [*wurthwe@uni-muenster.de*](mailto:wurthwe@uni-muenster.de) *Homepage:* [*https://www.uni-muenster.de/Chemie.oc/wuerthwein*](https://www.uni-muenster.de/Chemie.oc/wuerthwein)

**Table of Content**

1. Gibbs free energies without van der Waals interactions of carbenes **7**, alkynes **3** and
1,2,3-triazolyl-substituted nitrenes **8** Page S2

2. Formation of a Nucleophilic Carbene from 1,2-bis(diazo)alkane **1g** Page S2

3. Quantum Chemical Calculations Page S3

4. References Page S64

1. Gibbs free energies without van der Waals interactions of carbenes **7**, alkynes **3** and
1,2,3-triazolyl-substituted nitrenes **8**

Table: Gibbs free energies of carbenes **7**, alkynes **3** and 1,2,3-triazolyl-substituted nitrenes **8** [relative to 1,2-bis(diazo)alkanes **1**] and transition states **TS-7/3** and **TS-1/8** (values without van der Waal interaction, ΔG_298_ in kcal/mol).

| Entry | R, R | **TS-1**/**7** | **7** | **TS-7/3** | **3** | **TS-1/8** | **8** |
| --- | --- | --- | --- | --- | --- | --- | --- |
| **a** | H | 19.9 | –6.2 | –6.8 | –72.2 | 76.2 | 19.8 |
| **b** | R--R  (CH_2_)_3_ | 20.7 | –10.0 | 3.2 | –15.6 | 73.4 | 19.8 |
| **c** | R--R  (CH_2_)_4_ | 18.8 | –9.4 | –4.8 | –41.9 | 67.6 | 10.0 |
| **d** | R--R  (CH)_4_ | ^[a]^ | –21.8 | –18.7 | –49.1 | ^[b]^ | –6.3 |
| **e** | Ph | 22.7 | –3.4 | –3.6 | –78.2 | 76.5 | 18.8 |
| **f** | CN | 18.3 | –9.9 | –8.6 | –69.0 | 89.1 | ^[c]^ |
| **g** | NMe_2_ | 5.8 | –34.1 | –27.3 | –82.7 | 49.6 | 3.2 |

[a] Search for **TS-1d/7d** leads to minimum for alkyne **3d** and N_2_. [b] Search for **TS-1d/8d** leads via a barrier of 12.0 kcal/mol to benzyne **3d** and N_2_. [c] Search for minimum of **8f** leads to alkyne **3f** and N_2_.

2. Formation of a Nucleophilic Carbene from 1,2-bis(diazo)alkane **1g**

Another interesting process starting from 1,2,3,4-tetrazine **2g** leads to nucleophilic carbene **35**. If the C-C bond of **2g** is gradually elongated a transition state **TS-2g/35** could be localized. IRC-calculations lead either back to precursor **2g** or to the nucleophilic carbene **35** completing the 1,2 C→N shift of a carbon atom. The resulting carbene **35** has a Gibbs free energy of –19.9 kcal/mol and is considerable more stable than 1,2-bis(diazo)alkane **1g**. It can fragment into two molecules of dimethyl cyanamide and dinitrogen with an enormous energy relief of –94.2 kcal/mol (**1g** as reference compound). However, since the reaction barrier between **2g** and carbene **35** amounts to 72.7 kcal/mol it is very unlikely that this process is kinetically feasible.

3. Quantum Chemical Calculations

All calculations were performed using the GAUSSIAN 16, B.01 package of programs ^[1]^. The structures were fully optimized using the PBE1PBE/def2tzvp ^[2-6]^ + GD3BJ ^[7-8]^ functional including the PCM-solvent sphere for dichloromethane ^[9]^. Zero point vibrational energies and free enthalpy contributions were determined analytically.

| **Species a**  **R=H** | **E_tot_ [a.u.]** | **E_rel_ [kcal/mol]** | **G_298_ [a.u.]** | **E_rel_ [kcal/mol]** |
| --- | --- | --- | --- | --- |
| **N_2_** | -109.44698 |  | -109.45973 |  |
| **1a (172.1°)** | -296.07233 | 0.00 | -296.05609 | 0.00 |
| **1a (95.1°)** | -296.07227 | 0.04 | -296.05584 | 0.16 |
| **TS-1a, 0°** | -296.07172 | 0.38 | -296.05329 | 1.76 |
| **TS-1a, 120°** | -296.07225 | 0.05 | -296.05333 | 1.73 |
| **TS-1a/2a** | -296.05144 | 13.11 | -296.03110 | 15.68 |
| **2a** | -296.09362 | -13.36 | -296.06971 | -8.54 |
| **TS-2a/3a** | -296.03542 | 23.16 | -296.02020 | 22.52 |
| **3a** | -77.26038 |  | -77.25160 |  |
| **3a + 2N_2_** | -296.15433 | -51.46 | -296.17107 | -72.15 |
| **3a-vdw** | *-296.15669* | *-52.93* | *-296.16032* | *-65.40* |
| **TS-2a/4a** | -296.03919 | 20.80 | -296.02332 | 20.57 |
| **4a** | -93.35222 |  | -93.35504 |  |
| **2*4a+N_2_** | -296.15141 | -49.62 | -296.16981 | -71.36 |
| **4a-vdw** | *-296.15806* | *-53.80* | *-296.15689* | *-63.25* |
| **5a** | -186.61548 |  | -186.60102 |  |
| **5a+N_2_** | -296.06246 | 6.20 | -296.06075 | -2.92 |
| **5a´** | -186.59439 |  | -186.58058 |  |
| **5a´+N_2_** | -296.04136 | 19.43 | -296.04032 | 9.90 |
| **6a** | -295.96626 | 66.56 | -295.95043 | 66.30 |
| **TS 1a/7a** | -296.03936 | 20.69 | -296.02435 | 19.92 |
| **TS 3a/7a** | -186.61650 |  | -186.60722 |  |
| **TS-3a/7a+N_2_** | -296.06347 | 5.56 | -296.06696 | -6.82 |
| **TS 7a/3a-vdw** | *-296.06516* | *4.50* | *-296.06018* | *-2.56* |
| **7a** | -186.61678 |  | -186.60622 |  |
| **7a + N_2_** | -296.06376 | 5.38 | -296.06596 | -6.19 |
| **7a-vdw** | *-296.06547* | *4.31* | *-296.05911* | *-1.89* |
| **TS-1a/8a** | -295.95423 | 74.11 | -295.93467 | 76.20 |
| **TS-3a/8a** | -296.03870 | 21.11 | -296.02256 | 21.04 |
| **8a** | -296.04503 | 17.13 | -296.02453 | 19.81 |

Table S1 Species a R=H Total energies (E_tot_) and Gibbs free energies (G_298_) [a.u.], of compounds **a** and of the related transition states as given in the Gaussian archive entries. The relative energies [kcal/mol] include all involved species according to the respective reactions as given in the schemes in the manuscript. Van der Waals energies are given in italics.

**Gaussian Archive Entries**

(Total energies (a.u.), number of imaginary frequencies (for transition states: imaginary frequencies), coordinates)

**N_2_**

HF=-109.4469754 a.u., NIMAG=0

1\1\GINC-R02N47\FOpt\RPBE1PBE\def2TZVP\N2\WURTHWE\21-Jun-2021\0\\# pbe 1pbe/def2tzvp opt=(maxstep=5) freq pop=nbo emp=gd3bj scrf=(solvent=dichloro-methane)\\N2\\0,1\

N,0.,0., 0.105411813\N,0.,0.,1.194588187\\

Version=ES64L-G16RevB.01\State=1-SGG\HF=-109.4469754\ RMSD=1.373e-09\RMSF=1.242e-04\Dipole=0.,0.,0.\Quadrupole=0.399204,0.399204,-0.7984079,0.,0.,0.\PG=D*H [C*(N1.N1)]\\@

**1a 172.09°**

HF=-296.0723324 a.u., NIMAG=0

1\1\GINC-R08N41\FOpt\RPBE1PBE\def2TZVP\C2H2N4\WURTHWE\24-Apr-2025\0\\#

pbe1pbe/def2tzvp opt geom=check guess=read emp=gd3bj freq pop=nbo scrf=(solvent=dichloro methane)\\Bis-diazoethane 172 Grad\\0,1\

C,0.5062623284,-0.1625568513,0.432012361\N,-0.615173605,0.1909613196,0.9563663045\

N,-1.6185775333,0.4923265506,1.3957484278\C,1.0421955024,0.5536256052,-0.7038858551\

N,2.2604492282,0.3680612556,-1.0766651065\N,3.3280197073,0.1780082702,-1.4155902566\

H,0.4692137522,1.2675713647,-1.277873371\H,1.0070846197,-1.0016805147,0.893029496\\

Version=ES64L-G16RevB.01\State=1-A\HF=-296.0723324\RMSD=1.902e-09\RMSF=4.454e-06\Dipole=-0.0550262,-0.0954503,-0.0861413\Quadrupole=-6.1472351,4.2686128,1.8786223, 0.1267239,4.8246007,-3.0093033\PG=C01 [X(C2H2N4)]\\@

**1a 95.07°**

HF=-296.0722718 a.u., NIMAG=0

1\1\GINC-R01N18\FOpt\RPBE1PBE\def2TZVP\C2H2N4\WURTHWE\18-Jun-2025\0\\#

pbe1pbe/def2tzvp opt freq pop=nbo emp=gd3bj scrf=(solvent=dichloromethane)\\2 Bis-diazoethane 90 Grad\\0,1\

C,-0.597453,0.702482,-0.416595\N,-1.593441,-0.063537,-0.090638\N,-2.45527,-0.727393,0.202911\ C,0.5974533391,0.7024819951,0.416595\N,1.5934413328,-0.0635370131,0.090638\

N,2.4552703274,-0.7273930201,-0.202911\H,0.762294,1.321617,1.289593\

H,-0.7622936558,1.3216170062,-1.289593\\

Version=ES64L-G16RevB.01\State=1-A\HF=-296.0722718\RMSD=3.260e-09\RMSF=5.147e-06\Dipole=0.,0.7986742,0.\Quadrupole=-4.1742391,0.9954946,3.1787446,0.,2.5661972,0.\PG=C02 [X(C2H2N4)]\\@

**TS 1a, 0°**

HF=-296.0717205 a.u., NIMAG=1, -68.6690 cm-1

1\1\GINC-R02N38\FTS\RPBE1PBE\def2TZVP\C2H2N4\WURTHWE\15-Jan-2025\0\\# pbe1pbe/def2tzvp opt=(ts,noeigentest,calcfc) freq pop=nbo emp=gd3bj sc rf=(solvent=dichloromethane)\\ Bis-diazoethane-TS\\0,1\

C,0.0000931415,-0.0013632992,0.0002406958\C,0.0000428385,0.0001416756,1.4475153576\ N,1.0783761973,0.005573779,2.1498076679\N,2.0438234453,0.0103998899,2.7501512531\

N,1.0784754609,0.0025209171,-0.701987563\N,2.0439641169,0.0060376837,-1.3022738586\

H,-0.9307672485,-0.0033355854,1.9936146441\H,-0.930679548,-0.0059344277,-0.5459144183\\ Version=ES64L-G16RevB.01\State=1-A\HF=-296.0717205\RMSD=6.729e-09\RMSF=3.467e-07\Dipole=-1.5547609,-0.0067201,-0.000045\Quadrupole=1.8438215,-0.3264857,-1.5173358,0.0093886,0.0001099,-0.0014697\PG=C01 [X(C2H2N4)]\\@

**TS 1a, 120°**

TS for Rotation 119.98°

HF=-296.0722476 a.u., NIMAG=1 -16.5153cm-1

1\1\GINC-R01N34\FTS\RPBE1PBE\def2TZVP\C2H2N4\WURTHWE\17-Jan-2025\0\\# pbe1pbe/def2tzvp opt=(ts,noeigentest,calcfc) freq pop=nbo emp=gd3bj sc rf=(solvent=dichloro methane)\\ Bis-diazoethane-TS\\0,1\

C,0.5580546 06,0.50882103,0.4660693037\N,1.6722030567,-0.0561094526,0.121323579\ N,2.6504021942,-0.5148694544,-0.2059383364\C,-0.5580998209,0.5093143797,-0.4655351791\

N,-1.672238407,-0.0560065119,-0.1213983829\N,-2.6504289978,-0.5151372479,0.2053685679\

H,-0.5555849807,0.968651109,-1.4455657029\H,0.5555303495,0.9670951481,1.4465971507\\ Version=ES64L-G16RevB.01\State=1-A\HF=-296.0722476\RMSD=4.616e-09\RMSF=2.457e-07\Dipole=-0.0000043,0.6089902,0.0003285\Quadrupole=-5.741417,1.1882546,4.5531624,-0.001

3983,2.4610748,-0.0018005\PG=C01 [X(C2H2N4)]\\@

**TS-1 1a/2a**

HF=-296.0514423 a.u., NIMAG=1, -328.3962 cm^-1^

1\1\GINC-R03N20\FTS\RPBE1PBE\ def2TZVP\C2H2N4\WURTHWE\15-Jul-2021\0\\#pbe1pbe/ def2tzvp opt=(ts,noeigentest,calcfC,maxstep=5) freq pop=nbo emp=gd3bj scrf=(solvent= dichloromethane)\\ TS for ring closure of bis-diazoethane\\0,1\

C,-0.0004118998,-0.0013372271,-0.0007173191\C,-0.0003248712,0.0005706853,1.3949124951\ N,1.2620300155,-0.0053652301,1.8577309388\N,2.3553071953,0.2662028929,1.5746404971\ N,0.9795007648,0.7937937802,-0.4646905044\N,2.0019987704,1.2671818312,-0.1826859595\

H,-0.8090472258,0.0077539175,2. 1072782375\H,-0.6268537482,-0.5138745225,-0.7123685483\\

Version=ES64L-G16RevB.01\State=1-A\HF=-296.0514423\RMSD=9.751e-09\RMSF= 1.186e-05\Dipole=-1.394324,-0.490866,0.0005901\ Quadrupole=-0.4777475,-1.1711237,1.6488712, 0.279987,-0.5810021,1.65298\PG=C01 [X(C2H2N4)]\\@

**2a**

HF=-296.0936194 a.u., NIMAG=0

1\1\GINC-R03N35\FOpt\RPBE1PBE\def2TZVP\C2H2N4\WURTHWE\25-Jan-2025\0\\# pbe1pbe/def2tzvp opt=(maxstep=7) freq pop=nbo emp=gd3bj scrf=(solvent= dichloromethane)\\ 1,2,3,4-Tetrazine\\0,1\

N,-0.047785436,-0.0823595257,-0.101554141\N,0.0262915857,0.1022314197,1.1818625277\ N,1.1725831443,0.0289058287,1.8318611524\C,2.2643439988,-0.2387077937,1.1455506322\ C,2.1854780545,-0.433466041,-0.2245360006\N,1.0187116466,-0.3519230184,-0.8302882942\ H,3.1964163951,-0.2958847853,1.6957459651\H,3.0505122623,-0.6543930631,-0.8389964805\\

Version=ES64L-G16RevB.01\State=1-A\HF=-296.0936194\RMSD=3.373e-09\RMSF=1.635e-04\Dipole=2.2310623,-0.3451651,-0.0797144\Quadrupole=2.1255596,1.3545604,-3.4801199,-0.1492877,-0.3112048,-0.7050926\PG=C01 [X(C2H2N4)]\\@

**TS 2a/3a**

HF=-296.0354225 a.u., NIMAG=1, -621.1914 cm^-1^

1\1\GINC-R03N37\FTS\RPBE1PBE\def2TZVP\C2H2N4\WURTHWE\12-Feb-2025\0\\#pbe1pbe/ def2tzvp opt=(ts,noeigentest,calcfc) freq pop=nbo emp=gd3bj scrf=(solvent=dichloromethane)\\

TS for ring fragmentation of tetrazine to acetylene\\0,1\

N,0.1622539813,0.2130716189,-0.4947826278\N,0.2468751976,-0.2281852182,1.5006512098\ N,1.3111941006,-0.1827276536,1.9045914254\C,2.7254285046,0.1131431603,1.0240751046\ C,2.6746232043,-0.106003555,-0.2250631609\N,1.1898364256,0.1769000538,-0.9854665497\ H,3.4707774844,0.2557942394,1.7813893738\H,3.3560751015,-0.2419916456,-1.0414917753\\

Version=ES64L-G16RevB.01\State=1-A\HF=-296.0354225\ RMSD=4.938e-09\RMSF=1.547e-05\Dipole=1.3595009,0.0061854,-0.0562504\Quadrupole= 0.4718679,-0.2489228,-0.2229452, 0.055043,-0.0343611,1.2359964\PG=C01 [X(C2H2N4)]\\@

HF=-77.2603804 a.u., NIMAG=0

1\1\GINC-R08N26\FOpt\RPBE1PBE\def2TZVP\C2H2\WURTHWE\02-Jul-2021\0\\# pbe1pbe/def2tzvp opt=(maxstep=5) freq pop=nbo emp=gd3bj scrf=(solvent=dichloromethane)\\acetylene\\0,1\

H,-0.0004158243,0.,-0.0332045097\C,0.0035955197,0.,1.0331519452\

C,0.0126763246, 0.,2.2317221941\ H,0.0260297555,0.,3.2979648387\\

Version=ES64L-G16RevB.01\State=1-A'\HF=-77.2603804\RMSD=1.823e-09\RMSF=1.548e-04\Dipole=0.0045643,0.,-0.0000587\Quadrupole=-1.816674,-1.8170675,3.6337415,0.,0.0433481, 0.\PG=CS [SG(C2H2)]\\@

**3a-vdw**

HF=-296.1566895 a.u., NIMAG=0

1\1\GINC-R02N43\FOpt\RPBE1PBE\def2TZVP\C2H2N4\WURTHWE\08-Jun-2025\0\\#

pbe1pbe/def2tzvp opt=(maxstep=7) geom=check guess=read freq pop=nbo emp=gd3bj scrf=(solvent=dichloromethane)\\Acetylene + 2N2\\0,1\

C,0.052625298,-0.0779948935,-0.0037172202\C,0.1030870783,0.0018767525,1.1909715147\ N,3.4781602117,-0.2065102626,2.2905890282\N,4.1338217824,-0.175033792,1.4214308677\ N,2.8339476754,-3.0097396449,-0.2186181063\N,2.426243626,-2.501752993,-1.0915607208\ H,0.0099550502,-0.150875808,-1.0666767071\H,0.1508367612,0.0723275786,2.2538762956\\

Version=ES64L-G16RevB.01\State=1-A\HF=-296.1566895\RMSD=5.786e-09\RMSF=1.077e-05\Dipole=0.0025711,-0.0010663,-0.0003004\Quadrupole=-1.6412894,-1.2355925,2.8768818, 0.2364815,0.4406029,0.8252258\PG=C01 [X(C2H2N4)]\\@

**TS-2a/4a**

HF=- 296.0391867 a.u. NIMAG=1, -426.9706 cm^-1^

1\1\GINC-R03N30\FTS\RPBE1PBE\def2TZVP\C2H2N4\WURTHWE\24-Oct-2024\0\\# pbe1pbe/def2tzvp opt=(ts,noeigentest,calcfC,maxstep=5) freq pop=nbo emp=gd3bj scrf=(solvent=dichloromethane)\\TS for decomposition of tetrazine to 2 HCN + N2\\0,1\

N,0.1062474892,0.,0.0151855616\N,-0.1066149888,0.,1.9130815054\ N,0.7904661932,0.,2.6025749527\N,2.5686223844,0.,1.908941115\

C,2.5474077429,0.,0.6889629839\C,1.29062122,0.,-0.2781241746\

H,3.4755457419,0.,0.1200612184\H,1.6033580865,0.,-1.3211049558\\

Version=ES64L-G16RevB.01\State=1-A'\HF=-296.0391867\RMSD=8.463e-09\RMSF=1.043e-04\Dipole=1.1849014,0.,-1.5385274\Quadrupole=-1.5378332,0.3021967,1.2356365,0.,-5.2215737,0.\PG=CS [SG(C2H2N4)]\\@

**4a**

HF=-93.3522196 a.u., NIMAG=0

1\1\GINC-R04N05\FOpt\RPBE1PBE\def2TZVP\C1H1N1\WURTHWE\08-Jul-2021\0\\# pbe1pbe/ def2tzvp opt=(maxstep=6) freq pop=nbo emp=gd3bj scrf=(solvent=dichloromethane) \\

HCN\\0,1\

N,0.,0.,0.108991084\C,0.,0.,1.2545377158\H,0.,0.,2.3257372003\\

Version=ES64L-G16RevB.01\State=1-SG\HF=-93.3522196\RMSD=5.315e-09\RMSF=2.356e-05\Dipole=0.,0., 1.4109093\Quadrupole=-0.545163,-0.545163,1.0903259,0.,0.,0.\PG=C*V [C*(H1C1N1)]\\@

2 HCN+ N2

**4a-vdw**

HF=-296.158061 a.u., NIMAG=0

1\1\GINC-R03N33\FOpt\RPBE1PBE\def2TZVP\C2H2N4\WURTHWE\04-Jun-2025\0\\# pbe1pbe/def2tzvp opt=(maxstep=1,maxcycle=120) geom=check guess=read freq pop=nbo emp=gd3bj scrf=(solvent=dichloromethane)\\Products 2HCN + N2 from tetrazine\\0,1\

N,0.0887836711,1.8920365905,0.\N,0.6324647939,2.8358442695,0.\

N,2.5033573259,-1.2072519157,0.\C,1.358572438,-1.2686692811,0.\

C,-2.9510912227,-1.4084230321,0.\N,-1.8071874366,-1.407657441,0.\

H,0.2784551717,-1.3240435479,0.\H,-4.0225862144,-1.4091190625,0.\\

Version=ES64L-G16RevB.01\State=1-A'\HF=-296.158061\RMSD=4.934e-09\RMSF=1.278e-05\Dipole=-3.1070748,-0.0761438,0.\Quadrupole=3.970673,-2.2387008,-1.7319722,6.5552564,0.,0.\PG=CS [SG(C2H2N4)]\\@

**5a**

HF=-186.6154803 a.u., NIMAG=0

1\1\GINC-R03N04\FOpt\RPBE1PBE\def2TZVP\C2H2N2\WURTHWE\15-Jul-2021\0\\# pbe1pbe/def2tzvp opt=(maxstep=5) freq pop=nbo emp=gd3bj scrf=(solvent=dichloromethane)\\

N,N-four membered ring 5a\\0,1\

C,0.0321650863,0.,0.0254029014\N,-0.0486281826,0.,1.2979107198\ N,1.5497185342,0.,1.3390949359\C,1.5345657732,0.,0.0641148992\

H,2.3809285327,0.,-0.6182815427\H,-0.7779316163,0.,-0.6996751337\\

Version=ES64L-G16RevB.01\State=1-A1\HF=-186.6154803\RMSD=6.100e-09\RMSF=3.423e-05\Dipole=0.0480336,0.,-1.8641694\Quadrupole= 0.3773072,-0.2294948,-0.1478124,0.,0.0135396,0.\PG=C02V [SGV(C2H2N2)]\\@

**5a´**

HF=-186.5943869 a.u., NIMAG=0

1\1\GINC-R08N01\FOpt\RPBE1PBE\def2TZVP\C2H2N2\WURTHWE\02-Jul-2021\0\\# pbe1pbe/def2tzvp opt=(maxstep=5) freq pop=nbo emp=gd3bj scrf=(solvent=dichloromethane)\\

N,N-four membered ring 5a´\\0,1\

C,-0.0492513487,0.,0.0091568381\C,-0.0492513487,0.,1.3308431619\ N,1.4875645854,0.,0.0487524765\N,1.4875645854,0.,1.2912475235\

H,-0.7349174925,0.,2.1669516388\H,-0.7349174925,0.,-0.8269516388\\

Version=ES64L-G16RevB.01\State=1-A1\HF=-186.5943869\RMSD=4.146e-09\RMSF=1.651e-05\Dipole=-1.3844478,0.,0.\Quadrupole=-0.8672174,-0.4486831,1.3159005,0.,0.,0.\PG=C02V [SGV(C2H2N2)]\\@

**6a**

HF=-295.966255 a.u., NIMAG=0

1\1\GINC-R02N07\FOpt\RPBE1PBE\def2TZVP\C2H2N4\WURTHWE\24-Oct-2024\0\\# pbe1pbe/def2tzvp opt=(maxstep=5) freq pop=nbo emp=gd3bj scrf=(solvent=dichloromethane)\\

HC=N=N-N=N=CH from Tetrazine\\0,1\

H,3.5154337866,-0.5034904666,0.289103389\C,2.7203140902,-0.1132868538,-0.3347660292\ N,1.5864458324,0.0896526384,-0.0318421187\N,0.4240619985,0.4598566468,0.1368849472\

N,-0.4653025892,-0.5952524098,-0.1363353415\N,-1.6275927092,-0.2253891458,0.0335670048\ C,-2.7613820743,-0.0230510776,0.3370643042\H,-3.556890335,0.3679666684,-0.2858151558\\

Version=ES64L-G16RevB.01\State=1-A\HF=-295.966255\RMSD=8.347e-09\RMSF=6.277e-05\Dipole=-0.0006847,0.0003387,0.0009698\Quadrupole=7.7381408,-3.6940944,-4.0440464,-2.7635047,5.5445742,-0.6055918\PG=C01 [X(C2H2N4)]\\@

**TS-1a/7a**

HF=-296.0393614 a.u., NIMAG=1, -514.5907 cm^-1^

1\1\GINC-R02N08\FTS\RPBE1PBE\def2TZVP\C2H2N4\WURTHWE\22-Apr-2025\0\\# pbe1pbe/ def2tzvp opt=(ts,noeigentest,calcfc) freq pop=nbo emp=gd3bj scrf=(solvent=dichloromethane)\\

TS for N2-elimination from bis-diazoethane\\0,1\

C,0.4781209762,-0.5386196397,-0.4065976519\N,1.5870187687,-0.1577093835,0.215390377\

N,2.5229216594,0.2382384258,0.6807064915\C,-0.5047747957,0.4133185398,-0.7258847375\

N,-1.8298885823,0.3313021213,0.4203278749\N,-2.8381366229,-0.0732158763,0.622518537\

H,-0.2059542342,1.4437004382,-0.5249181127\H,0.4330798308,-1.6021906257,-0.6052387783\\

Version=ES64L-G16RevB.01\ State=1-A\HF=-296.0393614\RMSD=2.815e-09\RMSF=5.091e-05\Dipole=0.6168515,-0.2482377, 0.3826285\Quadrupole=-2.6714527,4.0202638,-1.3488111,-1.5471776,-1.5555778,0.3996835\ PG=C01 [X(C2H2N4)]\\@

**TS-3a/7a**

HF=-186.6164966 a.u., NIMAG=1, -406.5980cm^-1^

1\1\GINC-R01N18\FTS\RPBE1PBE\def2TZVP\C2H2N2\WURTHWE\31-Jul-2024\0\\# pbe1pbe/ def2tzvp opt=(ts,noeigentest,maxcycles=100,calcfc) freq pop=nbo emp=gd3bj scrf=(solvent=dichloro methane)\\TS for N2-elimination from carbene to ethyne\\0,1\

C,-0.762083264,0.467601775,0.0001502979\N,0.6775303416,0.1364383796,0.0001580578\ N,1.6351947897,-0.411196227,0.0001342697\C,-1.6133442581,-0.5425434482,0.0000189846\

H,-1.1766204546,-1.5433434955,-0.0000769475\H,-0.8706511546,1.5419000161,0.0002603375\\

Version=ES64L-G16RevB.01\State=1-A\HF=-186.6164966\RMSD=5.128e-09\RMSF=1.217e-05\Dipole=1.6199035,0.4523847,0.0000929\Quadrupole=-5.8330128,4.3028801,1.5301326,-0.5642884,-0.0002759,0.0002804\PG=C01 [X(C2H2N2)]\\@

**TS-3a/7a-vdw**

HF=-296.065156 a.u., NIMAG=1, -412.6483cm^-1^

1\1\GINC-R08N18\FTS\RPBE1PBE\def2TZVP\C2H2N4\WURTHWE\07-May-2025\0\\# pbe1pbe/ def2tzvp opt=(ts,noeigentest,calcfc) emp=gd3bj freq pop=nbo scrf=(solvent=dichloromethane)\\

TS for elimination of N2 to carbene - vdw\\0,1\

C,-1.4926906197,0.8056400772,0.4113732011\N,-1.3749250871,-0.6466117259,0.1596765867\

N,-1.1102418659,-1.6229774376,-0.2801049287\C,-0.9165927054,1.6175291664,-0.4562153281\ N,1.9792728774,-0.3221882325,0.030220493\N,2.9113951187,0.2338006556,0.1210669094\

H,-0.4028836634,1.1472790448,-1.2969242405\H,-2.0623670545,0.9540264519,1.3165373071\\

Version=ES64L-G16RevB.01\State=1-A\HF=-296.065156\RMSD=8.216e-09\RMSF=2.069e-06\Dipole=-0.2935916,-1.5917585,0.4113678\Quadrupole=2.8787921,-6.2630853,3.3842932,

2.5451775,-2.2189323,0.7772939\PG=C01 [X(C2H2N4)]\\@

HF=-186.6167804 a.u., NIMAG=0

1\1\GINC-R01N44\FOpt\RPBE1PBE\def2TZVP\C2H2N2\WURTHWE\31-Jul-2024\0\\# pbe1pbe/def2tzvp opt freq pop=nbo emp=gd3bj scrf=(solvent=dichloromethane)\\Carbene 7a\\0,1\

C,1.9005367051,0.7540544702,-0.387179916\N,1.7748163061,-0.6128372922,-0.0895795642\

N,1.5745683461,-1.6273141632,0.2992840692\C,1.3239528246,1.632480698,0.4509323306\

H,0.8126600241,1.1389516056,1.2869061835\H,2.4728637939,0.9033246817,-1.2942661031\\

Version=ES64L-G16RevB.01\State=1-A\HF=-186.6167804\ RMSD=2.821e-09\RMSF=7.420e-05\Dipole=0.3414929,-1.6800352,-0.4113818\Quadrupole= 2.5214822,-6.1695607,3.6480785, 0.8104653,-1.1212702,-0.6795509\PG=C01 [X(C2H2N2)]\\@

**7a-vdw**

HF=-296.0654659 a.u., NIMAG=0

1\1\GINC-R01N16\FOpt\RPBE1PBE\def2TZVP\C2H2N4\WURTHWE\31-Jul-2024\0\\# pbe1pbe/def2tzvp opt=(maxcycles=100,readfc) geom=check guess=read freq pop=nbo emp=gd3bj scrf=(solvent=dichloromethane)\\Carbene 7a + N2 (vdw)\\0,1\ C,1.0094638665,0.8751565463,-0.3673332096\N,1.4139416494,-0.4699374769,-0.3813796131\ N,1.6228186342,-1.5438512846,-0.2288700716\C,0.2348564811,1.2855926178,0.651370125\

N,-1.7589498212,-1.4735730247,-0.2557697525\N,-2.8400889925,-1.3525905593,-0.2036356288\ H,0.0201 318308,0.4812384715,1.3659439674\H,1.4002963518,1.4072807099,-1.2256308167\\ Version=ES64L-G16RevB.01\State=1-A\HF=-296.0654659\RMSD=8.913e-09\RMSF=2.173e-05\Dipole=0.867558,-1.3093718,-0.778932\Quadrupole=3.3576861,-6.0523871,2.694701,1.6142123,-1.3300156,-3.7258647\PG=C01 [X(C2H2N4)]\\@

**TS 1a/8a**

HF=-295.9542325 a.u., NIMAG=1, -561.1847 cm^-1^

1\1\GINC-R03N20\FTS\RPBE1PBE\def2TZVP\C2H2N4\WURTHWE\22-Jan-2025\0\\#pbe1pbe/ def2tzvp opt=(ts,noeigentest,calcfc) freq pop=nbo emp=gd3bj scrf=(solvent=dichloromethane)\\TS for N-N-ring closure of 1a giving 8a\\0,1\

C,-0.7135375467,1.0677281436,0.1343877332\C,-1.3343299486,-0.1921243062,-0.1320636356\

N,-0.4905632199,-1.1750739859,-0.095242357\N,0.6889630709,-0.7400303751,0.4630538384\

N,0.5760607853,0.8036997998,0.1534587054\N,1.4734096021,0.1053889185,-0.4542771974\

H,-2.3585724877,-0.3115278487,-0.4626167909\H,-1.0990352554,2.0705066539,0.054574704\\

Version=ES64L-G16RevB.01\State=1-A\HF=-295.9542325\RMSD= 4.782e-09\RMSF=1.439e-04\Dipole=-1.432632,1.370741,-0.137985\Quadrupole=1.4006918, 0.2957658,-1.6964576,-1

.2859326,1.0265912,0.2034171\PG=C01 [X(C2H2N4)]\\@

**TS-3a/8a**

**HF=-296.0386971**

1\1\GINC-R01N03\FTS\RPBE1PBE\def2TZVP\C2H2N4\WURTHWE\21-Jan-2025\0\\# pbe1pbe /def2tzvp opt=(ts,noeigentest,calcfc) freq pop=nbo emp=gd3bj scrf=(solvent=dichloromethane)\\TS-Suche für Ringöffnung\\0,1

\N,-1.5538084014,-0.3932629455,-0.0000363985\C,0.3210737802,1.2051695095,0.0000026177\ N,0.9788406454,-0.3085640783,0.0000918385\N,-0.792336821,-1.2545830293,0.0000779918\

C,-0.9480735924,1.1784222153,-0.0000056631\N,2.0494402137,-0.6531937313,-0.0001532313\ H,1.1243617027,1.9160162925,0.000132787\H,-1.7663315272,1.871500767,-0.0000549421\\ Version=ES64L-G16Rev B.01\State=1-A\HF=-296.0386971\RMSD=4.081e-09\RMSF=4.157e-05\Dipole=-0.2061535,1.5482553,0.0001087\Quadrupole=-1.874873,1.3419883,0.5328846,

-0.1693208,0.0005749,0.0001734\PG=C01 [X(C2H2N4)]\\@

HF=-296.0450335 a.u., NIMAG=0

1\1\GINC-R03N44\FOpt\RPBE1PBE\def2TZVP\C2H2N4\WURTHWE\20-Jan-2025\0\\# pbe1pbe/def2tzvp opt=(maxstep=5) freq pop=nbo emp=gd3bj scrf=(solvent =dichloromethane)\\ Five-membered ring 8a from 1a \\0,1\

N,0.5393215466,1.5158974617,-0.0467489481\C,-0.9872003599,-0.2138815979,0.0018290554\

N,0.315876372,-0.8284298052,0.1891321424\N,1.3199273906,0.6265145633,0.1223142716\

C,-0.8657777156,1.0839075561,-0.1291225149\N,0.6780009092,-1.9169876058,0.3414159133\

H,-1.8441658112,-0.8641011334,-0.0059992345\H,-1.6109043316,1.8454895611,-0.281295685\\ Version=ES64L-G16RevB.01\State=1-A\HF=-296.0450335\RMSD=5.233e-09\RMSF=4.487e-05\Dipole=-1.9177786,0.5640044,-0.238414\Quadrupole=1.8211404,-3.1484764,1.327336,-0.3945096,0.0852898,0.4569208\PG=C01 [X(C2H2N4)]\\@

Table S2 Species b R-R=(CH_2_)_3_ Total energies (E_tot_) and Gibbs free energies (G_298_) [a.u.], of compounds **b** and of the related transition states as given in the Gaussian archive entries. The relative energies [kcal/mol] include all involved species according to the respective reactions as given in the schemes in the manuscript. Van der Waals energies are given in italics.

| **Species b**  **R-R=**(CH_2_)_3_ | **E_tot_ [a.u.]** | **E_rel_ [kcal/mol]** | **G_298_ [a.u.]** | **E_rel_ [kcal/mol]** |
| --- | --- | --- | --- | --- |
| **N_2_** | -109.44698 |  | -109.45973 |  |
| **1b** | -412.71938 | 20.92 | -412.63955 | 0.00 |
| **TS-1b/2b** | -412.68757 | 40.88 | -412.60716 | 20.33 |
| **2b** | -412.75272 | 0.00 | -412.66833 | -18.06 |
| **3b** | -193.80762 |  | -193.74498 | - |
| **3b+2N_2_** | -412.70157 | 32.10 | -412.66444 | -15.62 |
| **3b-vdw** | *-412.70560* | *29.57* | *-412.64983* | *-6.45* |
| **TS-2b-4b** | -412.70979 | 26.94 | -412.63141 | 5.11 |
| **4b** | -303.38452 |  | -303.31462 |  |
| **4b+N_2_** | -412.83150 | -49.44 | -412.77435 | -84.59 |
| **4b-vdw** | *-412.83333* | *-50.59* | *-412.76842* | *-80.87* |
| **TS-1b-7b** | -412.68235 | 44.16 | -412.60664 | 20.65 |
| **TS-3b-7b** | -303.24222 |  | -303.17480 |  |
| **TS-3b-7b+N_2_** | -412.68920 | 39.86 | -412.63453 | 3.15 |
| **TS-3b-7b-vdw** | *-412.69161* | *38.34* | *-412.62778* | *7.39* |
| **7b** | -303.26774 |  | -303.19575 |  |
| **7b+N_2_** | -412.71472 | 23.85 | -412.65548 | -10.00 |
| **7b-vdw** | *-412.71702* | *22.40* | *-412.64889* | *-5.86* |
| **TS-2b-8b** | -412.60312 | 93.87 | -412.52255 | 73.42 |
| **8b** | -412.68922 | 39.84 | -412.60808 | 19.75 |

**Gaussian Archive Entries**

(Total energies (a.u.), number of imaginary frequencies (for transition states: imaginary frequencies), coordinates)

**1b**

HF=-412.7193848 a.u., NIMAG=0

1\1\GINC-R02N10\FOpt\RPBE1PBE\def2TZVP\C5H6N4\WURTHWE\12-Aug-2021\0\\# pbe1pbe/def2tzvp opt=(maxstep=8) freq pop=nbo emp=gd3bj scrf=(solvent=dichloromethane)\\

Bis-diazomethane / cyclopentane\\0,1\

C,0.0565869373,-0.0294421706,0.0276395326\C,-0.0365795598,0.036342878,1.5561532356\

C,1.4110081155,-0.0470721038,1.9651036773\C,2.2490542353,0.3243385717,0.8387217715\

C,1.3742518088,0.6610472112,-0.3412890499\N,1.8223617337,-0.3592066111,3.1356155486\

N,2.1901271174,-0.6378782072,4.1806958408\N,3.5260709288,0.4034005208,0.8467905267\

N,4.6667973443,0.4643437508,0.8492876157\H,1.2383002907,1.7453494648,-0.4222934222\

H,1.7877297568,0.3030017832,-1.2850568459\H,-0.8021498288,0.434319899,-0.4580439895\

H,0.1001013094,-1.0753198745,-0.2842193541\H,-0.6385218384,-0.7702676275,1.9767427138\

H,-0.4739107855,0.9874303175,1.8804138176\\

Version=ES64L-G16RevB.01\State=1-A\HF=-412.7193848\RMSD=9.332e-09\RMSF=1.883e-04\Dipole=-1.7129766,0.2468366,-1.1914916\Quadrupole=-1.2918432,2.4355247,-1.1436815,-0.3702975,0.0341273,1.0283018\PG=C01 [X(C5H6N4)]\\@

**TS-1b/2b**

HF=-412.6875741 a.u., NIMAG=1, -393.6403 cm^-1^

1\1\GINC-R02N42\FTS\RPBE1PBE\def2TZVP\C5H6N4\WURTHWE\05-Aug-2024\0\\#pbe1pbe/def2tzvp opt=(ts,noeigentest,calcfc) freq emp=gd3bj pop=nbo scrf=(solvent=dichloromethane)\\TS for formation of tetrazine / cyclopentane\\0,1\

C,-2.3253492244,0.0526157809,-0.2095127973\C,-1.4762664792,-1.2452232446,-0.2401286499\

C,-0.0945139767,-0.6952737472,-0.0985473546\C,-0.101406204,0.6748282594,0.1760392747\

C,-1.4768843144,1.160937377,0.4669910129\N,1.0781946076,-1.2518896439,0.1978054255\

N,2.2155351582,-1.0278284191,0.3008368257\N,1.0275079134,1.2689552144,-0.2033578552\

N,2.1592251611,1.0726057894,-0.3964179624\H,-1.6597622996,1.1954121908,1.5459661594\

H,-1.6803167019,2.1524065823,0.061648991\H,-3.2777361104,-0.1012509412,0.2972699301\

H,-2.5405991687,0.3574808651,-1.2353988979\H,-1.6392756072,-1.813861777,-1.1579646876\

H,-1.7124807539,-1.9019442863,0.6025565857\\

Version=ES64L-G16RevB.01\State=1-A\HF=-412.6875741\RMSD=4.208e-09\RMSF=2.547e-06\Dipole=-2.2771042,-0.0225181,0.1014555\ Quadrupole=-4.1404816,2.439493,1.7009885,-0.061348,0.0134349,1.4953905\PG=C01 [X(C5H6N4)]\\@

**2b**

HF=-412.7527184 a.u., NIMAG=0

1\1\GINC-R09N11\FOpt\RPBE1PBE\def2TZVP\C5H6N4\WURTHWE\12-Aug-2021\0\\# pbe1pbe/def2tzvp opt=(maxstep=8) freq pop=nbo emp=gd3bj scrf=(solvent=dichloromethane)\\

1,2,3,4- Tetrazine / cyclopentane\\0,1\

C,0.0038993862,0.1883094609,-0.0495774431\N,-0.0547028564,0.1748816134,1.2557399283\

N,1.0925180954,-0.0493246014,1.9057890454\N,2.2114312534,-0.2394378785,1.2971511696\

N,2.3199664871,-0.2286191229,-0.0358967169\C,1.2216423561,-0.0185488239,-0.7119387793\

C,1.0396149016,0.0479559369,-2.1880505296\C,-0.4868666328,-0.0971665849,-2.3458014383\

C,-1.0983030555,0.4112746161,-1.0253333409\H,1.3867757034,1.0266396196,-2.5379784392\

H,1.6168665745,-0.7096237791,-2.7180345725\H,-0.8658581743,0.4427813518,-3.2114410718\

H,-0.7366311634,-1.1521349062,-2.4754423771\H,-2.0222736253,-0.0911180732,-0.7392194907\

H,-1.3113077182,1.4852291378,-1.0705292834\\

Version=ES64L-G16RevB.01\State=1-A\HF=-412.7527184\RMSD=4.488e-09\RMSF=3.838e-05\Dipole=-1.392207,0.3238876,-2.6610366\Quadrupole=-1.6750461,4.3367922,-2.6617461,0.9633839,-0.6471763,-0.0482443\PG=C01 [X(C5H6N4)]\\@

**3b**

HF=-193.807618 a.u., NIMAG=0

1\1\GINC-R01N02\FOpt\RPBE1PBE\def2TZVP\C5H6\WURTHWE\13-Aug-2021\0\\# pbe1pbe/ def2tzvp opt=(maxstep=8) freq pop=nbo emp=gd3bj scrf=(solvent=dichloromethane)\\ cyclopentyne\\0,1\

C,-0.2596122742,0.5743799833,-0.0295620147\C,0.0282946905,-0.0762086694,1.4601499094\

C,1.4980031341,0.1717253276,2.0135627643\C,2.182130924,0.988085093,0.883631189\

C,0.9350712289,0.9156583318,0.1429706032\H,-0.71387673,0.3772898968,2.1125628893\

H,-0.1690589227,-1.1391798513,1.3449637699\H,2.0075573258,-0.7738780775,2.1847699885\

H,1.4669934295,0.7321402301,2.9452753426\H,3.0188349611,0.4792149238,0.4068188175\

H,2.475360393,1.9961682328,1.1737114892\\

Version=ES64L-G16RevB.01\State=1-A\HF=-193.807618\RMSD=5.626e-09\RMSF=4.342e-06\Dipole=1.6147627,-0.1315469,1.4047853\Quadrupole=-1.2098763,2.2934378,-1.0835615, 1.1037921,-4.3585403,-0.0743487\PG=C01 [X(C5H6)]\\@

**3b-vdw**

HF=-412.7055994 a.u., NIMAG=0

1\1\GINC-R09N19\FOpt\RPBE1PBE\def2TZVP\C5H6N4\WURTHWE\18-May-2025\0\\#

pbe1pbe/def2tzvp opt=(maxstep=6) freq pop=nbo emp=gd3bj scrf=(solvent =dichloro methane)\\Cyclopentyne + 2N2 vdw\\0,1\

C,-0.4117184415,1.0653258436,-1.4789831351\N,-0.725002318,-2.5859398347,-0.1437398951\ N,0.2695826287,-2.3014661972,0.1974024104\N,3.4665079178,-0.501437565,-0.2505198772\ N,2.8932806083,0.2532866656,0.2861053677\C,-0.1571524307,1.1797286027,-0.256074181\

C,-0.4934585113,1.1570603867,1.1558526698\C,-1.9559182243,0.828646308,0.7498025128\

C,-1.898912253,0.7589426238,-0.8374409897\H,0.0039838075,0.3697927979,1.7215018374\

H,-0.3732124228,2.1122575334,1.6648893549\H,-2.2831525927,-0.1216334329,1.1663604381\

H,-2.6399742715,1.6100642366,1.0729170445\H,-2.5582899062,1.5001724852,-1.2822114646\

H,-2.1579325904,-0.2359504536,-1.1927720929\\

Version=ES64L-G16RevB.01\State=1-A\HF=-412.7055994\RMSD=6.167e-09\RMSF=6.890e-06\Dipole =-0.8818825,-0.113657,1.9029495\Quadrupole=3.7235277,0.5432803,-4.266808,-1.3108495,-0.2794387,3.7942223\PG=C01 [X(C5H6N4)]\\@

**TS 2b/4b**

HF=-412.7097878 a.u., NIMAG=1, -434.0773 cm^-1^

1\1\GINC-R10N10\FTS\RPBE1PBE\def2TZVP\C5H6N4\WURTHWE\17-Sep-2024\0\\# pbe1pbe/def2tzvp opt=(ts,noeigentest,calcfC, maxstep=5) freq pop=nbo emp=gd3bj scrf=(solvent=dichloromethane)\\TS for formation of bis-nitrile 4b from 7b\\0,1\

N,-0.6117318543,1.4939191222,-0.518924406\C,0.3534966787,0.800258767,-0.2312413691\

C,0.3629903915,-0.7194980205,0.2147405848\N,-0.5929834594,-1.4698905711,0.3510764243\

N,-2.1767860504,-0.5793797676,-0.0450186503\N,-2.1835005439,0.5124572813,-0.3657271877\

C,1.8174605971,-1.0757760943,0.4387726792\C,2.6040220052,-0.0480490031,-0.3577312714\

C,1.802975194,1.2379599892,-0.2397817569\H,2.0099877396,-2.1104475038,0.1632378459\

H,2.0094109465,-0.9678212685,1.5110550262\H,3.6138622157,0.0729805591,0.0332859394\

H,2.6770584102,-0.3542890868,-1.40340774\H,1.9922811333,1.7282062688,0.7204890127\

H,1.9846825964,1.9622353283,-1.0309281311\\

Version=ES64L-G16RevB.01\State=1-A\HF=-412.7097878\RMSD= 8.259e-09\RMSF=1.874e-05\Dipole=2.7260974,0.0809814,0.2165313\Quadrupole=0.6804091,-3.1058454,2.4254363, 0.0819268,0.1866183,1.7791215\PG=C01 [X(C5H6N4)]\\@

**4b**

HF=-303.3845247 a.u., NIMAG=0

1\1\GINC-R03N05\FOpt\RPBE1PBE\def2TZVP\C5H6N2\WURTHWE\05-Jun-2025\0\\#

pbe1pbe/def2tzvp opt=(maxstep=8,maxcycle=200) freq pop=nbo emp=gd3bj

scrf=(solvent=dichloromethane)\\Dinitrile-4b\\0,1\

C,-2.6240981374,0.9441586655,1.0322415618\N,-2.7124313756,2.0676115372,1.2597451212\ N,0.0096202288,1.9159339287,-1.1927794022\C,-0.2450886714,0.8046033005,-1.0447221857\

C,-0.5448870289,-0.6077245849,-0.8717054973\C,-1.1042654132,-0.9794261396,0.5006760963\

C,-2.523795377,-0.4832998972,0.7748304328\H,-1.2396390492,-0.9046535067,-1.6626210577\ H,0.3838098551,-1.1576444101,-1.0445126288\H,-1.1293266905,-2.0688729259,0.5499816256\

H,-0.4291862539,-0.638688557,1.287285779\H,-2.9222638056,-0.99490694,1.6546891248\

H,-3.1873582814,-0.7272424705,-0.0598729696\\

Version=ES64L-G16RevB.01\State=1-A\HF=-303.3845247\RMSD=3.442e-09\RMSF=1.276e-05\Dipol

e=-0.208537,-3.6759325,-0.0525461\Quadrupole=1.5318547,-3.2079416,1.676087,-0.0416493, 2.4997381,0.064713\PG=C01 [X(C5H6N2)]\\@

4b-vdw

HF= -412.8333334 a.u., NIMAG=0

1\1\GINC-R03N04\FOpt\RPBE1PBE\def2TZVP\C5H6N4\WURTHWE\04-Sep-2024\0\\# pbe1pbe/def2tzvp opt=(maxstep=8,maxcycle=200,readfc) geom=check guess=read freq pop=nbo emp=gd3bj scrf=(solvent=dichloromethane)\\Dinitrile 4b + N2 vdw\\0,1\

C,0.3912239899,2.0577558414,0.9374985173\N,-0.4154399232,2.6638839826,1.4888734322\

N,-3.3190762932,-1.4976923754,-1.8141194289\N,-2.2429988533,-1.4837179571,-1.647732589\

N,-1.5326570659,-0.8080553509,1.9178382729\C,-0.5865662301,-0.9313885587,1.2763077236\

C,0.5999580335,-1.114870412,0.4567716518\C,0.9208457063,0.0521680857,-0.4755360701\

C,1.4183103956,1.3183585119,0.2206658122\H,1.443369602,-1.3186822167,1.1226799498\

H,0.4405964942,-2.0182085183,-0.1375817682\H,1.7180828457,-0.2822208507,-1.1407504985\

H,0.0573956594,0.281881739,-1.1014588214\H,1.839734163,1.9980701381,-0.5244696763\

H,2.222444476,1.0825689411,0.9236014924\\

Version=ES64L-G16RevB.01\State=1-A\HF=-412.8333334\RMSD=9.580e-09\RMSF=1.146e-04\Dipole=2.7932939,-1.1915005,-2.0506982\ Quadrupole=5.756055,-2.9532914,-2.8027636,1.1349008,3.6903446,-3.5462337\PG=C01 [X(C5H6N4)]\\@

**TS-1b/7b**

HF=-412.6823453 a.u., NIMAG=1, -534.2797cm^-1^

1\1\GINC-R01N25\Freq\RPBE1PBE\def2TZVP\C5H6N4\WURTHWE\17-Sep-2024\0\\#

N Geom=AllCheck Guess=TCheck SCRF=Check GenChk RPBE1PBE/def2TZVP Freq\

\TS for Carbene formation7b from 1b\\0,1\

N,-1.6095557696,-1.4107061695,1.0774821072\C,-0.6519955327,-0.8293166333,-0.2297850452\ C,-0.5567592915,0.5855551387,-0.138908105\N,-1.5795467749,1.3503599227,-0.4143818137\

N,-2.4592113584,2.0114590347,-0.6337524127\N,-2.6166284058,-1.8001079697,1.340393702\ C,0.7746638373,1.132288733,0.3049327634\C,1.6796416919,-0.0509224039,-0.0512024502\ C,0.7784694307,-1.2995460328,0.011393365\H,0.7675775372,1.3222878049,1.3850524794\ H,1.0604535932,2.0577245652,-0.1976863423\H,2.5440091746,-0.1242538595,0.6108569211\ H,2.0531831104,0.0804379947,-1.0690552808\H,1.0676466685,-2.0402695753,-0.738107089\

H,0.8562150892,-1.7919595498,0.9828552008\\

Version=ES64L-G16RevB.01\State=1-A\HF=-412.6823453\RMSD=7.290e-09\RMSF=6.597e-06\ZeroPoint=0.1099798\Thermal=0.1183526\Dipole=1.3091721,0.6300594,0.3109029\\\Polar=138.9640527,-8.5339688,141.6249648,-8.8520963,-29.4114207,98.7229883\ Quadrupole=-0.7007776, 0.3025633,0.3982143,-0.6713876,0.0577901,0.198683\PG=C01 [X(C5H6N4)]\NImag=1\\

**TS-3b/7b**

HF=-303.2422209 a.u., NIMAG=1, -388.0962 cm^-1^

1\1\GINC-R01N39\FTS\RPBE1PBE\def2TZVP\C5H6N2\WURTHWE\17-Sep-2024\0\\#

pbe1pbe/def2tzvp opt=(ts,noeigentest,calcfc,maxcycle=90,maxstep=5) freq pop=nbo emp=gd3bj scrf=(solvent=dichloromethane)\\TS cyclopentyne formation from 7b\\0,1\

C,0.0165652883,0.0194500227,0.1272426746\C,0.3665430382,-0.0690450769,1.6367096573\ C,1.6338649961,-0.0204638907,1.4551963042\C,2.4948913758,0.0366964889,0.2643822213\ C,1.3103377152,0.3379923385,-0.693869739\N,2.6082730602,-0.159154737,2.9987457044\ N,2.9212583906,-0.210162407,4.0425532483\H,3.2464016775,0.826670139,0.2801662014\

H,2.9889196995,-0.9148935876,0.0515642678\H,1.3271197409,1.3969633953,-0.9545202319\ H,1.3760187664,-0.2353640787,-1.6190606711\H,-0.4101605164,-0.9460196653,-0.159709614\

H,-0.7572643891,0.7709263422,-0.0411383464\\

Version=ES64L-G16RevB.01\State=1-A\HF=-303.2422209\RMSD=3.916e-09\RMSF=1.217e-05\Dipole=1.6254151,0.0628561,-0.7562141\Quadrupole=-2.0251559,0.4918088,1.5333471,-0.4506661,3.9254708,0.0304605\PG=C01 [X(C5H6N2)]

\\@

**TS-3b/7b-vdw**

HF=-412.6916125 a.u., NIMAG=1, -388.1847 cm^-1^

1\1\GINC-R02N20\FTS\RPBE1PBE\def2TZVP\C5H6N4\WURTHWE\02-Jun-2025\0\\#

pbe1pbe/def2tzvp opt=(ts,noeigentest,calcfc,maxstep=6) freq pop=nbo em

p=gd3bj scrf=(solvent=dichloromethane)\\TS für carbene formation from 7b vdw\\0,1\ C,0.3834088669,0.7839722296,-1.2437635913\N,-2.4742101755,-1.6713260099,-0.2918481731\

N,-1.5261767287,-2.1868986924,-0.1434077044\N,3.140716714,-1.106775935,-0.1662087299\ N,2.2381067437,-0.5370770478,0.0588698479\C,0.722001156,0.4822996241,-0.0456985006\ C,0.1265042562,0.710779334,1.2786372494\C,-0.97826295,1.6574405719,0.730941271\

C,-0.902502912,1.5440909627,-0.8313239695\H,-0.2713368272,-0.1997330091,1.7333180712\ H,0.7900753734,1.1961922621,1.995333616\H,-1.9623170241,1.3921303462,1.1184406393\

H,-0.7636529101,2.6800181023,1.0422667115\H,-0.9197405399,2.5291869653,-1.3017986473\

H,-1.7549430427,0.9889112961,-1.2325760902\\

Version=ES64L-G16RevB.01\State=1-A\HF=-412.6916125\RMSD=3.340e-09\RMSF=1.306e-06\ Dipole=0.0416883,-0.1788634,1.7656617\Quadrupole=2.4458598,1.3341101,-3.7799699,-1.3417933,2.4941573,1.8601945\PG=C01 [X(C5H6N4)]\\@

**7b**

HF= -303.2677434 a.u., NIMAG=0

1\1\GINC-R10N34\FOpt\RPBE1PBE\def2TZVP\C5H6N2\WURTHWE\02-Feb-2025\0\\# pbe1pbe/def2tzvp opt=(maxstep=5) freq pop=nbo emp=gd3bj scrf=(solvent=dichloromethane) \\Diazo-cyclopentylcarbene\\0,1\

C,-1.6348316869,-0.0082793415,-0.1616936657\C,-0.8108507211,-1.2538081521,0.1911902954\

C,0.5720578925,-0.640710119,0.1447360104\C,0.71765689,0.7257294241,0.141797673\

C,-0.7185975305,1.1801226508,0.1893428469\N,1.6695344378,-1.4117661924,0.1643540857\

N,2.5658078852,-2.0627970825,0.1692381896\H,-0.8787426929,1.5185845246,1.2250565071\

H,-0.8948557895,2.0640845876,-0.4293887258\H,-2.5924233269,0.0137185615,0.3608339462\

H,-1.8420806218,-0.0026368846,-1.2344443845\H,-0.9484852971,-2.077548971,-0.5114833557\

H,-1.0358914388,-1.6277270055,1.1951885774\\

Version=ES64L-G16RevB.01\State=1-A\HF=-303.2677434\RMSD=7.059e-09\RMSF=8.890e-06\Dipole=-1.0060002,-1.3807514,0.0635884\Quadrupole=-0.2888666,-1.3562208,1.6450874,-3.6740765,-0.1772413,0.0246976\PG=C01 [X(C5H6N2)]\\@

**7b-vdw**

HF=-412.7170182 a.u., NIMAG=0

1\1\GINC-R03N02\FOpt\RPBE1PBE\def2TZVP\C5H6N4\WURTHWE\06-Aug-2024\0\\#

pbe1pbe/def2tzvp opt=(maxstep=8) freq pop=nbo emp=gd3bj scrf=(solvent=dichloromethane)\\

azo-cyclopentylcarbene +N2 vdw\\0,1\

C,-0.3933428692,0.0230528387,-1.537664675\N,1.0954584444,-2.4051276071,1.6359372591\

N,1.5682534087,-1.4270599136,1.55728565\N,2.1482252088,1.8401974435,-0.3405870622\ N,1.1541952401,1.3543098125,-0.3936559168\C,-0.0459174524,0.756688615,-0.4291763382\

C,-1.0292833533,0.9194497477,0.7094772621\C,-2.028120801,-0.1789232829,0.3210840895\

C,-1.7932369814,-0.4093608355,-1.1848339713\H,-0.5845408598,0.771929199,1.6956102495\

H,-1.4822564052,1.9157981438,0.692480491\H,-1.7936838117,-1.0943571453,0.869560413\

H,-3.056134845,0.0960534488,0.5618558749\H,-2.4539858478,0.2243823214,-1.7967856596\

H,-2.0033880752,-1.4329977859,-1.506358666\\

Version=ES64L-G16RevB.01\State=1-A\HF=-412.7170182\RMSD=4.560e-09\RMSF=3.744e-06\Dipol

e=-0.3991932,0.6460895,1.5140365\Quadrupole=3.0908033,2.289403,-5.3802063,-0.4112706, 1.0776067,-0.495351\PG=C01 [X(C5H6N4)]\\@

**TS-2b/8b**

HF=-412.6031229 a.u., NIMAG=1, -431.6415 cm^-1^

1\1\GINC-R02N02\FTS\RPBE1PBE\def2TZVP\C5H6N4\WURTHWE\15-Mar-2025\0\\#pbe1pbe/ def2tzvp opt=(ts,noeigentest,calcfC,maxstep=6) freq pop=nbo emp=gd3bj scrf=(solvent= dichloromethane)\\TS for cyclopentyl-five-membered ring\\0,1\

N,-0.0178208866,0.0507076539,0.0232490998\ N,-0.0000535292,0.0462626924,1.4146904542\ C,1.2350682008,-0.002700347,1.7937265785\C,2.1174948148,-0.3470162261,0.7231648341\ N,1.3531847726,-0.6598068791,-0.2917392138\C,3.4761727042,-0.7050019369,1.1925125783\ C,3.4485780671,-0.1129425936,2.6228109193\C,1.9729251644,-0.1038977543,3.0858515787\ N,0.2124794864,-1.2755605472,-0.5240025128\H,4.2735434282,-0.284120523,0.5786481094\ H,3.6048332687,-1.7938081474,1.1986479316\H,3.8171366721,0.9138286585,2.5854481497\ H,4.0876040462,-0.66978761,3.3060513313\H,1.7428659419,0.7145737438,3.7683365825\ H,1.702066264,-1.0391012767,3.5848004337\\

Version=ES64L-G16RevB.01\State=1-A\HF=-412.6031229\ RMSD=6.695e-09\RMSF=4.704e-05\Dipole=2.4348076,-0.0279128,1.4148322\Quadrupole=-1.2826808,0.4461911,0.8364897,-0.3243572,-1.6942367,-0.5663468\PG=C01 [X(C5H6N4)]\\@

**8b**

HF=-412.6892224 a.u., NIMAG=0

1\1\GINC-R01N02\FOpt\RPBE1PBE\def2TZVP\C5H6N4\WURTHWE\15-Mar-2025\0\\#

pbe1pbe/def2tzvp opt=(maxstep=5) freq pop=nbo emp=gd3bj scrf=(solvent= dichloromethane)\\Cyclopentyl-five-membered ring 8b\\0,1\

C,0.7311365529,-1.3415517364,0.0045425976\ C,1.4486248425,-0.0644288811,-0.5155505759\

C,0.4830539961,1.1328518462,-0.300285927\C,-0.8038487665,0.4302961197,-0.0968113294\

C,-0.6756656251,-0.8712873136,0.0597911022\ H,0.8669707205,-2.201486606,-0.653813709\

H,1.0708827278,-1.638773049,1.0016683851\ H,1.6392994678,-0.1774994339,-1.5836156274\

H,2.4096380033,0.0898366064,-0.0264823747\ H,0.4664441016,1.8206520482,-1.1480944631\

H,0.7382628722,1.7230461955,0.5863879534\N,-2.164327726,0.9199208918,0.0083162233\

N,-1.9068714518,-1.5219246587,0.3054204638\N,-2.2643578502,-2.6021438421,0.5074415958\

N,-2.970879865,0.060428813,0.2171656853\\

Version=ES64L-G16RevB.01\State=1-A\HF=-412.6892224\RMSD=4.813e-09\RMSF=8.789e-06\Dipole=2.5037345,0.6084421,-0.3652034\Quadrupole=-2.0069135,-2.0721897,4.0791032,-0.6632221,1.0080372,1.0226426\PG=C01 [X(C5H6N4)]\\@

Table S3 Species c R-R=(CH_2_)_4_**:** Total energies (E_tot_) and Gibbs free energies (G_298_) [a.u.] of compounds **c** and of the related transition states as given in the Gaussian archive entries. The relative energies [kcal/mol] include all involved species according to the respective reactions as given in the schemes in the manuscript. Van der Waals energies are given in italics).

| **Species c**  **R-R=**(CH_2_)_4_ | **E_tot_ [a.u.]** | **E_rel_ [kcal/mol]** | **G_298_ [a.u.]** | **E_rel_ [kcal/mol]** |
| --- | --- | --- | --- | --- |
| **N_2_** | -109.44698 |  | -109.45973 |  |
| **1c** | -452.00808 | 22.77 | -451.90004 | 0.00 |
| **TS-1c/2c** | -451.98838 | 35.13 | -451.87930 | 13.01 |
| **2c** | -452.04436 | 0.00 | -451.93203 | -20.07 |
| **TS-2c/3c** | -451.97714 | 42.18 | -451.87423 | 16.20 |
| **3c** | -233.14102 |  | -233.04733 |  |
| **3c+2N_2_** | -452.03497 | 5.89 | -451.96680 | -41.89 |
| **3c-vdw** | *-452.03903* | *3.34* | *-451.95376* | *-33.71* |
| **TS-2c/4c** | -451.98217 | 39.02 | -451.87581 | 15.20 |
| **4c** | -342.66535 |  | -342.56776 |  |
| **4c+N_2_** | -452.11233 | -42.65 | -452.02750 | -79.98 |
| **4c-vdw** | *-452.11437* | *-43.94* | *-452.01954* | *-74.99* |
| **5c** | -342.55839 |  | -342.45643 |  |
| **5c+N_2_** | -452.00537 | 24.47 | -451.91617 | -10.12 |
| **5c´** | -342.53592 |  | -342.43414 |  |
| **5c’+N_2_** | -451.98290 | 38.57 | -451.89387 | 3.87 |
| **TS-1c/7c** | -451.97439 | 43.91 | -451.87005 | 18.82 |
| **TS 3c/7c** | -342.54481 |  | -342.44791 |  |
| **TS 3c/7c + N_2_** | -451.99178 | 32.99 | -451.90765 | -4.78 |
| **TS-3c/7c-vdw** | *-451.99410* | *31.54* | *-451.90170* | *-1.04* |
| **7c** | -342.55494 |  | -342.45523 |  |
| **7c+N_2_** | -452.00192 | 26.63 | -451.91496 | -9.37 |
| **7c-vdw** | *-452.00432* | *25.12* | *-451.90753* | *-4.70* |
| **TS-2c/8c** | -451.90081 | 90.08 | -451.79232 | 67.59 |
| **8c** | -451.99370 | 31.78 | -451.88419 | 9.95 |

**Gaussian Archive Entries**

(Total energies (a.u.), number of imaginary frequencies (for transition states: imaginary frequencies), coordinates)

**1c**

HF=-452.0080762 a.u., NIMAG=0

1\1\GINC-R08N35\FOpt\RPBE1PBE\def2TZVP\C6H8N4\WURTHWE\01-Jul-2021\0\\# pbe1pbe/def2tzvp opt=(maxstep=5) freq pop=nbo emp=gd3bj scrf=(solvent=dichloromethane)\\Bis-diazoalkane / cyclohexane\\0,1\

C,0.0109670015,0.0706046156,0.0109931119\C,-0.0042832827,-0.0065635033,1.5119813501\

C,1.2565092281,0.0473345328,2.2389983722\ C,2.4882810992,-0.5472408505,1.6158094294\

C,2.5053014601,-0.2752447645,0.1149378169\ C,1.2019445079,-0.7028286523,-0.5460013664\

N,-1.1177105484,-0.1155729475,2.1473927878\N,-2.1011715664,-0.1802462129,2.7178807518\

N,1.3141335663,0.5943465691,3.4021043805\ N,1.3508505858,1.0517778871,4.4443372771\

H,3.3790749881,-0.1355498748,2.0938250527\ H,2.4990476857,-1.6320850005,1.7836560446\

H,2.6685485848,0.7941611203,-0.0590296804\ H,3.3473500429,-0.8069046043,-0.3351733304\

H,1.2580140358,-0.5439674598,-1.6257802696\ H,1.0464080708,-1.7754385769,-0.385166641\

H,0.0862771521,1.1197920026,-0.3031696649\H,-0.9261876155,-0.3217182964,-0.3881713653\\

Version=ES64L-G16RevB.01\State=1-A\HF=-452.0080762\RMSD=7.992e-09\RMSF=3.894e-06\Dipole=1.0076211,-0.4180611,-1.716429\ Quadrupole=-0.3061181,1.9557586,-1.6496405,-1.3228862,2.1500661,-2.0858684\PG=C01 [X(C6H8N4)]\\@

**TS 1c/2c**

HF=-451.9883767 a.u., NIMAG=1, -334.9511 cm^-1^

1\1\GINC-R08N26\FTS\RPBE1PBE\def2TZVP\C6H8N4\WURTHWE\12-Jul-2021\0\\#pbe1pbe/ def2tzvp opt=(ts,noeigentest,calcfC,maxstep=5) freq pop=nbo emp=gd3bj scrf=(solvent= dichloromethane)\\TS for bis-diazoalkane / cyclohexane to give 1,2,3,4-tetrazine\\0,1\

C,-0.0003570977,0.0001659661,0.0004274838\C,-0.0003222407,0.0002213379,1.5297732981\

C,1.4147553874,-0.0009905523,2.116202032\C,2.207602411,-1.0589730497,1.4365916001\

C,1.9951742226,-1.4192284516,0.0936032679\C,0.6856247006,-1.2375219805,-0.5860045221\

N,3.0778829065,-1.888358287,2.0077061057\N,3.961069233,-2.6216608215,1.83781081\

N,3.1421644904,-1.7793368279,-0.477514296\N,4.2113254382,-2.1972439799,-0.307622428\

H,0.8262269185,-1.1351826799,-1.6636747705\H,0.0547597742,-2.1206553188,-0.4231430764\

H,0.5177440256,0.8961343895,-0.3593324343\H,-1.0277434982,0.0559893219,-0.3669648418\

H,-0.5464432485,0.8722243623,1.8971678579\H,-0.5335759601,-0.8868122174,1.8895333911\

H,1.8821985781,0.9785051118,1.9533401093\H,1.3932591281,-0.1735603699,3.193872228\\

Version=ES64L-G16RevB.01\State=1-A\HF=-451.9883767\RMSD=3.760e-09\RMSF=2.502e-05\Dipole=-1.9032589,1.1222652,0.0000052\Quadrupole=-3.6634748,0.4873772,3.1760976, 3.7521717,1.0719349,1.8178597\PG=C01 [X(C6H8N4)]\\@

**2c**

HF=-4 52.0443556 a.u., NIMAG=0

1\1\GINC-R08N01\FOpt\RPBE1PBE\def2TZVP\C6H8N4\WURTHWE\02-Jul-2021\0\\# pbe1pbe/def2tzvp opt=(maxstep=5) freq pop=nbo emp=gd3bj scrf=(solvent=dichloromethane)\\1,2,3,4-Tetrazine / cyclohexane\\0,1\

N,-0.0500051003,0.2202234157,0.0058619418\C,-0.025969638,0.1469251664,1.3243384611\

C,1.2018452848,-0.0431970669,1.9706561575\N,2.3017681999,-0.1224806627,1.2437997415\

N,2.2178313053,-0.0344945363,-0.0677399322\N,1.078093537,0.1248781173,-0.6677500881\

C,1.3103403918,-0.172643812,3.4520125359\C,0.0651622786,0.3317185943,4.1679753695\

C,-1.192578348,-0.2137120276,3.5080438543\C,-1.3072929667,0.283360634,2.074178538\

H,-1.5741103839,1.3473767138,2.0674218196\H,-2.0815024191,0.084042073,4.0668296909\

H,0.044155969,1.4264072059,4.1442942145\H,2.2167914481,0.339359016,3.782106663\

H,1.4630879442,-1.2361036838,3.6729960252\H,0.1076771462,0.0385188368,5.2183360927\

H,-1.1641955056,-1.3085292044,3.5174394308\H,-2.0941331488,-0.2304035983,1.5174055249\\

Version=ES64L-G16RevB.01\State=1-A\HF=-452.0443556\RMSD= 9.556e-09\RMSF=1.348e-04\Dipole=-1.4222172,0.0082348,2.7039856\Quadrupole=-9289395,5.060568,-4.1316285, 0.4156442,2.3230106,0.183404\PG=C01 [X(C6H8N4)]\\@

**TS-2c/3c**

HF=-451.9771352 a.u., NIMAG=1, -459.7250 cm^-1^

1\1\GINC-R02N27\FTS\RPBE1PBE\def2TZVP\C6H8N4\WURTHWE\13-Jul-2021\0\\#

pbe1pbe/def2tzvp opt=(ts,noeigentest,calcfC,maxstep=4) geom=check gues s=read freq pop=nbo emp=gd3bj scrf=(solvent=dichloromethane)\\TS for alkyne formation / cyclohexane\\0,1\

N,-0.071411056,0.0029919068,-0.2564416816\C,-0.1421256353,-0.0075584404,1.5141437901\

C,0.9097000805,0.0140499736,2.2049374034\N,2.5041154651,-0.1253083578,1.4442327986\

N,2.6823613953,-0.1634435774,0.3262793978\N,0.8843095991,-0.0465929729,-0.8624022336\

C,1.0272203797,0.0758791843,3.6694926722\C,-0.3579557771,0.4705529969,4.2082271976\

C,-1.4963307418,-0.2613180252,3.4966277117\C,-1.5341013814,0.0291601334,1.9870218783\

H,-1.9661992652,1.015182343,1.7818076328\H,-2.454069443,0.0120454616,3.9462035187\

H,-0.488080336,1.5497397878,4.0757432717\H,1.7935614022,0.7938621433,3.9779164034\

H,1.3409063739,-0.9009916489,4.0547014211\H,-0.4025195862,0.2746984397,5.2823616145\

H,-1.3735480751,-1.3399372009,3.6402718075\H,-2.154548698,-0.7001116736,1.4568227542\\

Version=ES64L-G16RevB.01\State=1-A\HF=-451.9771352\RMSD=4.008e-09\RMSF=1.204e-05\Dipole=-1.1015272,0.0851903,1.6744755\Quadrupole=-0.3335442,2.5437067,-2.2101625, 0.2935255,2.1571372,-0.1213304\PG=C01 [X(C6H8N4)]\\@

**3c**

HF=-233.1410157 a.u., NIMAG=0

1\1\GINC-R01N31\FOpt\RPBE1PBE\def2TZVP\C6H8\WURTHWE\02-Jul-2021\0\\# pbe1pbe/def2tzvp opt=(maxstep=5) freq pop=nbo emp=gd3bj scrf=(solvent=dichloromethane)\\Cyclohexyne\\0,1\

C,-0.236182152,-0.2107874878,0.2233508916\C,0.0644562432,0.3099334875,1.2766491084\

C,-0.617367396,1.3162097052,2.1034957991\C,-2.0620520104,1.2763929853,1.5121408725\

H,-0.6372690453,1.098402648,3.1730238385\H,-0.1574913976,2.3004474852,1.9788300499\

C,-2.1364147557,1.1475929323,-0.0121408725\H,-2.5894441911,0.4339148383,1.9702007595\

H,-2.586071205,2.1873390661,1.8179026712\C,-1.4485547394,-0.1234490042,-0.6034957991\

H,-3.1873268005,1.1459338265,-0.3179026712\H,-1.6705033686,2.025567032,-0.4702007595\

H,-2.0709916611,-1.0138321913,-0.4788300499\H,-1.2698791193,0.0026898583,-1.6730238385\\

Version=ES64L-G16RevB.01\State=1-A\HF=-233.1410157\RMSD=4.059e-09\RMSF=2.180e-05\Dipole=-1.0662033,0.6155728,0.\Quadrupole=-2.4414549,0.0340468,2.4074082,2.1438474, 0.638262,1.1055023\PG=C02 [X(C6H8)]\\@

**3c-vdw**

HF=-452.0390291 a.u., NIMAG=0

1\1\GINC-R03N12\FOpt\RPBE1PBE\def2TZVP\C6H8N4\WURTHWE\17-May-2025\0\\#

pbe1pbe/def2tzvp opt=(maxstep=6) freq pop=nbo emp=gd3bj scrf=(solvent= dichloromethane)\\Cyclohexyne + 2N2 vdw\\0,1\

C,2.1946961943,0.672509451,-0.2067578314\C,2.0811954431,0.3909060391,1.3258141047\ C,0.8948153607,-0.4740109427,1.3780353697\C,0.3013864307,-1.0925026794,0.5202419003\ C,0.4636310549,-1.1393594655,-0.9393243022\C,1.8871625511,-0.5221499863,-1.1146556595\

N,-1.2787014862,2.2305444483,0.5094208134\N,-1.6774755277,1.9790772619,-0.4725229853\

N,-3.1752818381,-1.3307026845,-0.7878615588\N,-3.2680729693,-1.2267217714,0.292453629\ H,0.4233835261,-2.1380670437,-1.3783131528\H,-0.3009306261,-0.5345132767,-1.4357164106\ H,2.620870013,-1.3127594788,-0.930002787\H,2.0040505305,-0.2135958804,-2.1582654653\ H,3.2084317014,1.0266770996,-0.4181228849\H,1.5134322969,1.4933167787,-0.4493370038\ H,2.9634972862,-0.1309555255,1.7064001413\H,1.9921970587,1.3280806563,1.8787080832\\ Version=ES64L-G16RevB.01\State=1-A\HF=-452.0390291\RMSD=5.336e-09\RMSF=1.470e-06\Dipole=0.7742937,0.4519776,-0.8082902\Quadrupole=3.2608091,-0.4964604,-2.7643486, 0.5093731,0.1995861,2.1577095\PG=C01 [X(C6H8N4)]\\@

**TS-2c/4c**

HF=-451.9821708 a.u., NIMAG=1, -429.1143 cm^-1^

1\1\GINC-R10N14\FTS\RPBE1PBE\def2TZVP\C6H8N4\WURTHWE\12-Jul-2021\0\\#pbe1pbe/def2tzvp opt=(ts,noeigentest,calcfc,maxstep=5) freq pop=nbo emp=gd3bj scrf=(solvent=dichloromethane)\\TS for dinitrile formation from tetrazine\\0,1\

N,-0.0704382078,-0.4370602487,0.0537630425\C,-0.0200302496,-0.1318044676,1.2379647295\

C,1.3192922975,0.0991450568,2.0644823401\N,2.3557866856,0.5609571404,1.6056504492\

N,2.3546721909,0.516331373,-0.2871798482\N,1.5861247712,-0.1335955652,-0.8102209191\

C,1.2308127645,-0.3624512633,3.5062232281\C,-0.0541587039,0.0252739123,4.2125733027\

C,-1.259237488,-0.4743571486,3.4405332336\C,-1.2806218966,0.1160649386,2.0436650342\

H,-1.3704827486,1.2067570106,2.1130369526\H,-2.1868440399,-0.198572678,3.9474513969\

H,-0.1047487169,1.1132358421,4.3231043216\H,2.1123941528,0.0047364148,4.0295031005\

H,1.3057131725,-1.455717211,3.4655506325\H,-0.0363467691,-0.3966596946,5.2201132725\

H,-1.2401422433,-1.5673986184,3.3825763828\H,-2.1266885439,-0.2436222657,1.4600399468\\

Version=ES64L-G16RevB.01\State=1-A\HF=-451.9821708\RMSD=2.962e-09\RMSF=6.352e-05\Dipole=-1.4354643,-0.2278137,2.3903476\Quadrupole=-0.7122758,2.212695,-1.5004192,-2.9531544,0.1786418,-1.4218148\PG=C01 [X(C6H8N4)]\\@

**4c**

HF=-342.6653549 a.u., NIMAG=0

1\1\GINC-R03N45\FOpt\RPBE1PBE\def2TZVP\C6H8N2\WURTHWE\02-Jul-2021\0\\# pbe1pbe/def2tzvp opt=(maxstep=5) freq pop=nbo emp=gd3bj scrf=(solvent=dichloromethane)\\1,4-dinitrile 4c\\0,1\

C,-0.058334872,-0.6785996386,0.2823426411\C,-0.2085595161,0.0159491734,1.5499573895\ C,1.0754766725,0.7153287756,2.0163991133\H,-1.0389965913,0.7219019768,1.4599817485\

H,-0.5081296122,-0.7337132246,2.2868241177\C,1.3398437412,2.0936375648,1.42969099\ H,1.0082557626,0.8252101763,3.1006617148\H,1.9278305796,0.0581238271,1.8245649761\

C,1.4569029557,2.167621826,-0.0988497688\H,0.5694400663,2.7948300656,1.7612853548\ H,2.2839596846,2.4535670439,1.8436879955\C,0.169764016,2.2564610918,-0.7675508155\ H,2.0210076517,3.0596930064,-0.3829604323\H,2.0006380693,1.3079244296,-0.5005005691\ N,0.0578572814,-1.2331773928,-0.7186106049\N,-0.8587800121,2.3573136657,-1.2724606452\\

Version=ES64L-G16RevB.01\State=1-A\HF=-342.6653549\RMSD=9.992e-09\RMSF=4.631e-04\Dipole=1.5743903,0.8140732,2.6457209\Quadrupole=2.5806033,-2.350626,-0.2299773,4.0705402,-5.3839231,-3.054422\PG=C01 [X(C6H8N2)]\\@

**4c-vdw**

HF=-452.1143709 a.u., NIMAG=0

1\1\GINC-R03N33\FOpt\RPBE1PBE\def2TZVP\C6H8N4\WURTHWE\04-Jun-2025\0\\#

pbe1pbe/def2tzvp opt=(maxstep=6) freq pop=nbo emp=gd3bj scrf=(solvent

=dichloromethane)\\1,4-dinitrile 4c + N2 -vdw\\0,1\

C,1.4203599123,1.6727189146,-0.6367860347\C,0.0303787749,1.7414344328,-0.2185063643\ C,0.1664022431,-1.3107665632,-0.8552390025\C,0.9603852401,-1.3136671794,0.3615630526\ C,2.3161895347,-0.6128872561,0.2013295226\C,2.3102462186,0.9012070283,0.3472138469\

N,-0.4324572141,-1.3227628929,-1.8371753085\N,-3.5353327268,-0.8551660922,0.7802697319\ N,-2.5782937885,-1.165044116,1.1973508589\N,-1.057815576,1.8102613181,0.1476791874\ H,1.4623772397,1.2383480468,-1.639498774\H,3.3339213977,1.2513735761,0.1996315293\ H,2.74991216,-0.8937767542,-0.7621432912\H,1.1163062764,-2.3610481003,0.6325472062\ H,0.3683658569,-0.8643266451,1.1636512958\H,2.9802534648,-1.0160899207,0.9686394693\ H,2.0329620821,1.1771786706,1.3680887393\H,1.7737889041,2.7034495329,-0.7229826651\\ Version=ES64L-G16RevB.01\State=1-A\HF=-452.1143709\RMSD=3.488e-09\RMSF=7.026e-06\Dipole=2.9611386,-0.0834627,1.0773156\Quadrupole=2.0327157,1.1799884,-3.2127041, 3.9429426,-1.95444,-5.5634972\PG=C01 [X(C6H8N4)]\\@

**5c**

HF=-342.5583896 a.u., NIMAG=0

1\1\GINC-R08N01\FOpt\RPBE1PBE\def2TZVP\C6H8N2\WURTHWE\02-Jul-2021\0\\# pbe1pbe/def2tzvp opt=(maxstep=5) freq pop=nbo emp=gd3bj scrf=(solvent=dichloromethane)\\ Four-membered ring 5c / cyclohexane\\0,1\

C,0.035052618,-0.0193046733,0.0246270186\C,-0.0458418527,-0.036105572,1.5654012341\

C,1.3337985957,0.1510938577,2.044121506\C,2.4554480927,0.6710711855,1.2336489242\

C,2.3866157009,1.0057848792,-0.1984633336\C,0.885404807,1.1318210665,-0.5319879558\

N,2.0485797361,0.0331730067,3.1023029202\N,3.2848648416,0.630640162,2.2109285963\

H,2.9563290398,1.9008660415,-0.4585026376\H,2.8182310791,0.1718591366,-0.7662210148\

H,0.5073705012,2.0816559928,-0.1383149355\H,0.7666379681,1.1737712188,-1.6160036909\

H,-0.975371676,0.0524319047,-0.3813900496\H,0.4463459952,-0.9725492297,-0.3250143401\

H,-0.6459237516,0.8160175307,1.908759903\H,-0.5178041674,-0.9403066666,1.9565884637\\

Version=ES64L-G16RevB.01\State=1-A\HF=-342.5583896\ RMSD=2.207e-09\RMSF=2.025e-05\Dipole=-1.6780263,0.171675,-2.2126596\Quadrupole=-0.090976,3.1467812,-3.0558052, 0.4072091,-5.2834655,0.4850935\PG=C01 [X(C6H8N2)]\\@

**5c´**

HF=-342.535922 a.u., NIMAG=0

1\1\GINC-R08N02\FOpt\RPBE1PBE\def2TZVP\C6H8N2\WURTHWE\15-Jul-2021\0\\# pbe1pbe/def2tzvp opt=(maxstep=5) freq pop=nbo emp=gd3bj scrf=(solvent=dichloromethane)\\ Four-membered ring 5c´ / cyclohexane \\0,1\

C,-0.009689443,-0.0193209655,0.0333905452\C,0.0114474909,0.0598489085,1.5676379794\

C,1.4263341084,0.0421921237,2.1803008665\C,2.1508135762,-0.969903001,1.4009553052\

C,1.7882828202,-1.5596487954,0.2781674554\C,0.5920083812,-1.3185161358,-0.5387699905\

N,3.4387261703,-1.834645979,1.3944814754\N,3.0960674676,-2.3933422448,0.3398986383\

H,0.8323908093,-1.1978143117,-1.5995244043\H,-0.1147480525,-2.1544097109,-0.4685933537\

H,0.5535047463,0.826299417,-0.3737746175\H,-1.0393909391,0.0878468469,-0.314580025\

H,-0.5103049869,0.9653194362,1.8850544061\H,-0.5474312514,-0.7886963874,1.9747060749\

H,1.9093097632,1.0242530348,2.1072354539\H,1.3823524093,-0.2086359326,3.2445941284\\

Version=ES64L-G16RevB.01\State=1-A\HF=-342.535922\RMSD =9.882e-09\RMSF=3.831e-05\Dipole=-1.6257909,1.061746,-0.0331403\Quadrupole=-3.3166542,0.073727,3.2429272, 3.8572269,0.0851509,0.4163289\PG=C01 [X(C6H8N2)]\\@

**TS-1c/7c**

HF=-451.9743855 a.u. NIMAG=1, -541.8208 cm^-1^

1\1\GINC-R01N14\FTS\RPBE1PBE\def2TZVP\C6H8N4\WURTHWE\18-Feb-2025\0\\#pbe1pbe/def2tzvp opt=(ts,noeigentest,calcfC,maxstep=5) freq pop=nbo emp=gd3bj scrf=(solvent=dichloromethane)\\TS for N2-elimination from tetrazine to give carbene \\0,1\

C,2.1612674984,0.6438924745,-0.1750621062\C,1.1537125231,1.1372839883,0.8588496627\

C,-0.2361288749,0.5902955364,0.7266418611\C,-0.2861791268,-0.7098347356,0.162247609\

C,0.7793503781,-1.4060412676,-0.6491409036\C,2.1513974772,-0.8760827626,-0.2505814962\

N,-1.0291621112,1.6613166589,-0.3711627023\N,-1.9967284741,2.133553451,-0.6414159487\

N,-1.4556986777,-1.3105203292,0.2528085309\N,-2.4507274038,-1.8196787874,0.3148765546\

H,0.7285143309,-2.4890438074,-0.5115103859\H,0.6079437664,-1.2084251482,-1.7158014816\

H,2.4269060496,-1.2852085854,0.7276998399\H,2.8950505249,-1.2334333369,-0.9669864361\

H,3.1646377748,1.0015614458,0.073519586\H,1.9046214321,1.0643770597,-1.1550969451\

H,1.482130599,0.8140653442,1.856244998\H,1.1219903141,2.2293248018,0.8922547635\\

Version=ES64L-G16RevB.01\State=1-A\HF=-451.9743855\RMSD=5.444e-09\RMSF=4.223e-05\Dipole= 1.2342861,-0.6283397,-0.4723682\Quadrupole=-1.6762722,1.1424404,0.5338317, 0.8627369, 0.7853805,0.8678864\PG=C01 [X(C6H8N4)]\\@

**Ts-3c/7c**

HF=-342.5448065 a.u., NIMAG=1, -523.8097 cm^-1^

1\1\GINC-R01N39\FTS\RPBE1PBE\def2TZVP\C6H8N2\WURTHWE\17-Apr-2025\0\\#

pbe1pbe/def2tzvp opt=(ts,noeigentest,calcfc,maxstep=6) freq pop=nbo emp=gd3bj scrf=(solvent=dichloromethane)\\ \\TS for N2-elimination from tetrazine to give alkyne \\0,1\

C,0.0548310965,1.3341702377,0.0695153779\C,-0.527489704,-0.0194421834,-0.0147570674\

C,-0.1472865445,-1.2498589762,-0.1028905518\C,1.3578579895,-1.3847982399,-0.1155624869\ C,2.1009769777,-0.13300712,0.3605488287\C,1.5386035387,1.1320308826,-0.2783452965\

N,-2.1791232132,0.0622718045,0.0245861081\N,-3.2490413494,-0.1733093568,0.0403445032\ H,1.6636218569,-2.2572162307,0.4704241293\H,1.6424126667,-1.6247470924,-1.1486158962\ H,2.0086859902,-0.0554752684,1.4501655096\H,3.1697559202,-0.2191088889,0.1417291637\ H,2.1043400805,2.0119161078,0.0357266138\H,1.6351787298,1.057464928,-1.3664734376\

H,-0.055693592,1.7550639079,1.0749880038\H,-0.4095794437,2.0331044883,-0.6314625018\\ Version=ES64L-G16RevB.01\State=1-A\HF=-342.5448065\RMSD=6.595e-09\RMSF=1.101e-05\ Dipole=0.0515987,1.5643212,0.0830991\Quadrupole=2.7704184,-3.0272466,0.2568282,-2.5441424,-0.3696942,-0.2807454\PG=C01 [X(C6H8N2)]\\@

**TS-3c/7c-vdw**

HF=-451.9941003 a.u., NIMAG=, -524.2158 cm^-1^

1\1\GINC-R02N01\FTS\RPBE1PBE\def2TZVP\C6H8N4\WURTHWE\17-May-2025\0\\#

pbe1pbe/def2tzvp opt=(ts,noeigentest,calcfc,maxstep=6) freq pop=nbo emp=gd3bj scrf=(solvent=dichloromethane)\\ for N2-elimination from tetrazine to give alkyne vdw\\0,1\

C,2.1906146497,-0.0069886894,0.0997918364\C,1.480533616,-0.0922902376,1.4545071863\

C,-0.0066380115,-0.3420666123,1.3576481226\C,-0.3581530928,-0.7560632958,0.1870094759\ C,0.2470802882,-1.0848545796,-1.1176016899\C,1.7575316131,-1.130289598,-0.8358091171\

N,-0.3622243781,3.0670244243,-0.5902979944\N,-1.1525978055,2.3977167553,-0.9273128758\

N,-1.9995653821,-0.9541183067,0.1223638251\N,-3.0727024086,-0.9598102278,0.3431874088\ H,-0.0976436126,-2.0460502032,-1.5083202947\H,0.0132576426,-0.3221626711,-1.8690568838\ H,1.9980695238,-2.09403999,-0.3752287935\H,2.3012043601,-1.0806783345,-1.7817717504\ H,3.2759192576,-0.0327160355,0.2375869866\H,1.9570079616,0.9571419695,-0.3662645601\ H,1.8990172447,-0.9130919362,2.0518247202\H,1.6726755338,0.8103565686,2.0431193979\\ Version=ES64L-G16RevB.01\State=1-A\HF=-451.9941003\RMSD=5.504e-09\RMSF=7.527e-07\Dipole=0.0820841,-0.4641726,-1.4753261\Quadrupole=2.440613,1.2790817,-3.7196947,0.1173173,1.7314681,1.0087311\PG=C01 [X(C6H8N4)]\\@

**7c**

HF=-342.55494 a.u., NIMAG=0

1\1\GINC-R09N47\FOpt\RPBE1PBE\def2TZVP\C6H8N2\WURTHWE\02-Feb-2025\0\\# pbe1pbe/def2tzvp opt=(maxstep=5) freq pop=nbo emp=gd3bj scrf=(solvent= dichloromethane)\\Diazo-cyclohexyl-carbene 7c\\0,1\C,-0.6015996705,-1.4537051067,0.4193407313\

C,0.5974565145,-0.6482979462,-0.0262113323\C,0.7331317074,0.6267538216,-0.4986023962\

C,-0.5706526603,1.3560579016,-0.5708695525\C,-1.7652215626,0.7484097276,0.162757555\

C,-1.8521129987,-0.749013909,-0.0906981824\N,1.7867076865,-1.3109542435,0.0437865149\

N,2.7457346682,-1.8574079817,0.1095912895\H,-0.4140455085,2.3978727623,-0.2696136359\

H,-0.7871708122,1.4284544537,-1.64900233\H,-1.6592510677,0.9254261124,1.239402517\

H,-2.6909510847,1.2421816894,-0.1459598513\H,-2.733061778,-1.1794072289,0.3907498152\

H,-1.9533520627,-0.9286970627,-1.1665875183\H,-0.6230287814,-1.5223449512,1.5125991023\

H,-0.5467585894,-2.4760490388,0.0351042736\\

Version=ES64L-G16RevB.01\State=1-A\HF=-342.5549419\RMSD=6.459e-09\RMSF=7.985e-06\Dipole=-0.9934776,-1.2207537,0.5397087\ Quadrupole=-1.2655686,0.4105966,0.854972,-3.4846499,1.7501604,0.6978059\PG=C01 [X(C6H8N2)]\\@

**7c-vdw**

HF=-452.004323 a.u., NIMAG=0

1\1\GINC-R01N24\FOpt\RPBE1PBE\def2TZVP\C6H8N4\WURTHWE\17-May-2025\0\\#

pbe1pbe/def2tzvp opt=(maxstep=6) geom=check guess=read freq pop=nbo emp=gd3bj scrf=(solvent=dichloromethane)\\Diazo-cyclohexyl-carbene 7c +N2 vdw\\0,1\ C,1.8187861889,1.2680510232,0.0295618429\C,1.5521867494,0.4256850625,1.2766610756\ C,0.5320109739,-0.6633384407,1.180193785\C,0.1710164182,-0.9351012021,-0.1092817289\ C,0.6092969202,-0.3612633357,-1.4366019414\C,1.9140411027,0.3913472658,-1.2099321119\

N,-1.8179451045,2.2344454016,1.2019656157\N,-2.1993721153,1.5636685025,0.4332366208\

N,-0.7702674922,-1.9168340918,-0.195121239\N,-1.5394523438,-2.7069729444,-0.277014554\

H,0.7404058353,-1.1541191665,-2.1782705779\H,-0.1590352391,0.3171416555,-1.8245228978\ H,2.7283113095,-0.3302705137,-1.082468184\H,2.1484973618,0.9867407229,-2.0951630061\ H,2.7353381113,1.8511721242,0.1558058824\H,1.005505218,1.9904425173,-0.1038445097\ H,2.4755091398,-0.087213467,1.5905915529\H,1.3093239657,1.0630498862,2.1346233754\\ Version=ES64L-G16RevB.01\State=1-A\HF=-452.004323\RMSD=6.454e-09\RMSF=1.407e-05\Dipole=0.1895416,0.2911352,-1.608051\Quadrupole=2.2114722,0.292115,-2.5035872,

0.1847247,-0.0509421,3.1839076\PG=C01 [X(C6H8N4)]\\@

**TS-2c/8c**

HF=-451.9008101 a.u., NIMAG=1, -495.3180 cm^-1^

1\1\GINC-R02N05\FTS\RPBE1PBE\def2TZVP\C6H8N4\WURTHWE\15-Mar-2025\0\\#pbe1pbe/def2tzvp opt=(ts,noeigentest,calcfC,maxstep=6) freq pop=nbo emp=gd3bj scrf=(solvent=dichloromethane)\\TS for Cyclohexyl-five-membered ring\\0,1\

N,-0.0297861373,-0.0771791324,0.0269435844\N,0.0618437181,-0.0214335219,1.3485688783\

C,1.0080342641,-0.0144491908,2.2656175243\C,2.1097164324,0.7245235784,1.7108616451\

N,1.7828497877,1.2755637538,0.5842205914\N,0.4140639421,1.2311097338,0.4319455414\

C,3.4754488786,0.6096194281,2.2949593925\C,3.3965222716,0.0828185229,3.7251522884\

C,2.5244263984,-1.1623336863,3.8192525559\ C,1.061895949,-0.8622763669,3.4773378689\

H,3.9720874778,1.5808543951,2.249865151\ H,4.0613975512,-0.0750397627,1.6729022033\

H,2.9940032423,0.8612954034,4.3818851336\ H,4.4048643458,-0.1383058389,4.0797375683\

H,2.9058664969,-1.9253708751,3.1330521386\ H,2.5716512721,-1.5849979331,4.824294984\

H,0.5999296504,-0.3209937749,4.3114900943\ H,0.482189346,-1.7767442069,3.3317362872\\

Version=ES64L-G16RevB.01\State=1-A\HF=-451.9008101\RMSD=5.419e-09\RMSF=2.747e-05\Dipole=1.3493602,-0.9269781,2.4243832\ Quadrupole=1.3579496,0.9324928,-2.2904424,0.936091,-3.0744067,0.8087633\PG=C01 [X(C6H8N4)]\\@

**8c**

HF=-451.9937048 a.u., NIMAG=0

1\1\GINC-R01N02\FOpt\RPBE1PBE\def2TZVP\C6H8N4\WURTHWE\15-Mar-2025\0\\# pbe1pbe/def2tzvp opt=(maxstep=5) freq pop=nbo emp=gd3bj scrf=(solvent=dichloro methane)\\Cyclohexyl-five-membered ring 8c\\0,1\

C,-0.0821730698,1.4042712845,0.6145823758\C,-1.2971874454,0.8051983966,-0.0977778703\

C,-1.3570335032,-0.7129131319,0.0474821195\C,-0.1351533833,-1.395817529,-0.5693255918\

C,1.0734570816,-0.6406909901,-0.1817134218\C,1.0999389149,0.5671938671,0.3413663862\

H,0.1049697445,2.4312511174,0.2868349456\H,-0.2451872071,1.4544967911,1.6980425923\

H,-1.2453877825,1.0623088476,-1.1609430999\H,-2.2108092079,1.2567283151,0.2933920759\

H,-1.4088252971,-0.9722621375,1.1099205548\H,-2.2649889339,-1.1000382202,-0.4190854984\

H,-0.2069799643,-1.4233525726,-1.6626824062\H,-0.044562467,-2.4353935592,-0.2407730698\

N,2.4060067672,-1.1687023554,-0.3334283486\N,2.4659341018,1.021440118,0.6349302853\

N,3.3252730986,0.2459469969,0.3399211115\N,2.8413735528,-2.1639382385,-0.73071114\\

Version=ES64L-G16RevB.01\State=1-A\HF=-451.9937048\ RMSD=4.100e-09\RMSF=1.458e-05\Dipole=-2.6049132,0.5471762,0.0669423\Quadrupole=-3.9416296,0.0166027,3.925027, 1.5297131,0.1092246,-1.9683398\PG=C01 [X(C6H8N4)]\\@

Table S4 Species d R=(CH)_4_**:** Total energies (E_tot_) and Gibbs free energies (G_298_) [a.u.] of compounds **d** and of the related transition states as given in the Gaussian archive entries. The relative energies [kcal/mol] include all involved species according to the respective reactions as given in the schemes in the manuscript. Van der Waals energies are given in italics.

| **Species d**  **R=(CH)_4_** | **E_tot_ [a.u.]** | **E_rel_ [kcal/mol]** | **G_298_ [a.u.]** | **E_rel_ [kcal/mol]** |
| --- | --- | --- | --- | --- |
| **N_2_** | -109.44698 |  | -109.45973 |  |
| **1d** | -449.57361 | 30.58 | -449.51249 | 0.00 |
| **TS-1d/2d** | -449.56008 | 39.07 | -449.49776 | 9.25 |
| **2d** | -449.62235 | 0.00 | -449.55481 | -26.55 |
| **TS-2d/3d** | -449.56705 | 34.70 | -449.50939 | 1.95 |
| **3d** | -230.72001 |  | -230.67131 |  |
| **3d+2N_2_** | -449.61396 | 5.26 | -449.59077 | -49.12 |
| **3d-vdw** | *-449.61830* | *2.54* | *-449.57940* | *-41.98* |
| **TS-2d-4d** | -449.53332 | 55.87 | -449.47423 | 24.01 |
| **4d** | -340.21394 |  | -340.16251 |  |
| **4d+N_2_** | -449.66092 | -24.20 | -449.62224 | -68.87 |
| **4d-vdw** | *-449.65511* | *-20.56* | *-449.60538* | *-58.29* |
| **5d** | -340.14749 |  | -340.08989 |  |
| **5d+N_2_** | -449.59447 | 17.49 | -449.54963 | -23.30 |
| **5d’** | -340.10376 |  | -340.04700 |  |
| **5d´+N_2_** | -449.55073 | 44.94 | -449.50673 | 3.62 |
| **TS-3d/7d** | -340.13473 |  | -340.08254 |  |
| **TS-3d/7d+N_2_** | -449.58171 | 25.50 | -449.54227 | -18.69 |
| **TS-3d/7d-vdw** | *-449.58434* | *23.85* | *-449.53543* | *-14.39* |
| **7d** | -340.14272 |  | -340.08747 |  |
| **7d+N_2_** | -449.58970 | 20.49 | -449.54721 | -21.78 |
| **7d-vdw** | *-449.59233* | *18.83* | *-449.54095* | *-17.86* |
| **TS-5/1d-8d** | -449.55449 | 42.58 | -449.49341 | 11.98 |
| **8d** | -449.58745 | 21.90 | -449.52257 | -6.32 |

**Gaussian Archive Entries**

(Total energies (a.u.), number of imaginary frequencies (for transition states: imaginary frequencies), coordinates)

**1d**

HF=-449.5736135 a.u., NIMAG=0

1\1\GINC-R08N37\FOpt\RPBE1PBE\def2TZVP\C6H4N4\WURTHWE\13-Jul-2021\0\\#

pbe1pbe/def2tzvp opt freq pop=nbo emp=gd3bj scrf=(solvent=dichloromethane)\\ Benzo-bis(diazo)alkane 1d\\0,1\

C,-0.01361665,0.,-0.0044994865\C,-0.0225773926,0.,1.401547471\

C,1.177283032,0.,2.0569371851\C,2.401196302,0.,1.3461881587\

C,2.4265190507,0.,-0.0209051649\C,1.2007687195,0.,-0.7099154062\

H,-0.9715573602,0.,1.9232592213\H,1.2013225218,0.,3.1398718838\

H,3.329613428,0.,1.9041391905\H,3.3498212062,0.,-0.586745448\

N,1.1683285881,0.,-2.0612713946\N,0.0387785749,0.,-2.6617406326\

N,-1.1335684902,0.,-1.9810425939\N,-1.1714567529,0.,-0.7023229837\\

Version=ES64L-G16RevB.01\State=1-A'\HF=-449.6223467\RMSD=9.374e-09\RMSF=1.728e-04\Dipole=1.590564, 0.,2.7394225\Quadrupole=0.2091396,0.7246899,-0.9338296,0.,-1.0023659,0.\PG=CS [SG(C6H4N4)]\\@

**TS-1d/2d**

HF=-449.5600769 a.u., NIMAG=1, -310.3248 cm^-1^

1\1\GINC-R03N42\FTS\RPBE1PBE\def2TZVP\C6H4N4\WURTHWE\14-Dec-2024\0\\#pbe1pbe/def2tzvp opt=(ts,noeigentest,calcfC,maxstep=3,maxcycles=200) geom=check guess=read freq pop=nbo emp=gd3bj scrf=(solvent=dichloromethane)\\TS for Benzotetrazine-formation\\0,1\

C,2.3093806626,-0.7288326021,-0.0643942711\C,1.1490299606,-1.4390192783,-0.0711546073\

C,-0.0590914614,-0.7073844936,0.0456039429\C,-0.0441115712,0.7201007632,-0.0583683843\

C,1.1756915349,1.4279889683,0.080740153\ C,2.3218469807,0.6952313838,0.0953516714\

N,-1.234489331,-1.1684252196,0.4912310687\N,-2.372302737,-1.0748310956,0.6019010765\

N,-1.2021987342,1.2044324721,-0.5243429898\N,-2.3396370337,1.1337320325,-0.654210225\

H,1.1352956464,-2.5200712995,-0.1481837296\H,3.2539054096,-1.2496816954,-0.1629842791\

H,3.2744139101,1.1975213609,0.211336841\H,1.1816877635,2.5091297035,0.1575427327\\

Version=ES64L-G16RevB.01\State=1-A\HF=-449.5600769\RMSD=1.773e-09\RMSF=2.976e-05\Dipole=1.5961389,-0.0160359,0.014153\ Quadrupole=-3.5555883,5.6005096,-2.0449213,0.0802251,0.0022631,1.3564792\PG=C01 [X(C6H4N4)]\\@

**2d**

HF=-449.6223467 a.u., NIMAG=0

1\1\GINC-R08N37\FOpt\RPBE1PBE\def2TZVP\C6H4N4\WURTHWE\13-Jul-2021\0\\# pbe1pbe/def2tzvp opt freq pop=nbo emp=gd3bj scrf=(solvent=dichloromethane)\\ Benzotetrazine\\0,1\

C,-0.01361665,0.,-0.0044994865\C,-0.0225773926,0.,1.401547471\

C,1.177283032,0.,2.0569371851\C,2.401196302,0.,1.3461881587\

C,2.4265190507,0.,-0.0209051649\C,1.2007687195,0.,-0.7099154062\

H,-0.9715573602,0.,1.9232592213\H,1.2013225218,0.,3.1398718838\

H,3.329613428,0.,1.9041391905\H,3.3498212062,0.,-0.586745448\

N,1.1683285881,0.,-2.0612713946\N,0.0387785749,0.,-2.6617406326\

N,-1.1335684902,0.,-1.9810425939\N,-1.1714567529,0.,-0.7023229837\\

Version=ES64L-G16RevB.01\State=1-A'\HF=-449.6223467\RMSD=9.374e-09\RMSF=1.728e-04\Dipole= 1.590564,0.,2.7394225\Quadrupole=0.2091396,0.7246899,-0.9338296,0.,-1.0023659,0.\PG=CS [SG(C6H4N4)]\\@

**TS-2d/3d**

HF=-449.5670471 a.u., NIMAG=1, -430.7919 cm^-1^

1\1\GINC-R01N14\FTS\RPBE1PBE\def2TZVP\C6H4N4\WURTHWE\26-Aug-2024\0\\# pbe1pbe/def2tzvp opt=(ts,noeigentest,calcfc) freq pop=nbo scrf=(solvent=dichloromethane) emp=gd3bj\\TS for benzyne formation from benzotetrazine 2d\\0,1\

C,0.0655228341,0.630944987,0.0050655708\C,1.1426432406,1.48327205,0.0123184871\

C,2.3825977405,0.8467390681,0.0066616489\C,2.4805410163,-0.5471829011,-0.0050042372\

C,1.3417594739,-1.3507904443,-0.0115019538\C,0.156118842,-0.6573154723,-0.0058183037\

H,1.0404315639,2.5640362075,0.0219130905\H,3.2883795701,1.4426069822,0.0113483567\

H,3.4607340934,-1.0105632293,-0.0089217145\H,1.3915898743,-2.4352439894,-0.019914742\

N,-1.3105490728,-1.7042306036,-0.0138944427\N,-2.3649570748,-1.3067629432,-0.0113137526\

N,-2.5219499838,0.9217840329,0.0066918797\N,-1.5335111178,1.4628502555,0.0123701128\\

Version=ES64L-G16RevB.01\State=1-A\HF=-449.5670471\ RMSD=6.193e-09\RMSF=4.184e-05\Dipole=1.6025141,0.111726,0.0012239\Quadrupole=-0.6243949,2.0522379,-1.4278431,-0.1878062,-0.0033303,0.029005\PG=C01 [X(C6H4N4)]\\@

**3d**

HF=-230.7200121 a.u., NIMAG=0

1\1\GINC-R01N08\FOpt\RPBE1PBE\def2TZVP\C6H4\WURTHWE\13-Jul-2021\0\\# pbe1pbe/def2tzvp opt freq pop=nbo emp=gd3bj scrf=(solvent=dichloromethane)\\Benzyne\\0,1\

C,0.1221612714,0.,0.0528611598\C,-0.0576432303,0.,1.4179815839\

C,1.1907581566,0.,2.0610236571\ C,2.4019553318,0.,1.361738642\

C,2.4692652542,0.,-0.0409297099\C,1.1971340368,0.,-0.5677746557\

H,-0.994180299,0.,1.9594316413\H,1.2139599806,0.,3.1460780489\

H,3.3300390877,0.,1.9243592068\H,3.4064432931,0.,-0.5812695742\\

Version=ES64L-G16RevB.01\State=1-A1\HF=-230.7200121\RMSD=2.992e-09\RMSF=1.005e-04\Dipole= 0.4329481,0.,0.7498882\Quadrupole=2.819327,-3.2093943,0.3900674,0.,-2.1038005,0.\PG=C02V [SGV(C6H4)]\\@

**3d-vdw**

HF=-449.6183016 a.u., NIMAG=0

1\1\GINC-R03N07\FOpt\RPBE1PBE\def2TZVP\C6H4N4\WURTHWE\12-May-2025\0\\#

pbe1pbe/def2tzvp opt=(maxstep=2,calcfc) geom=check guess=read freq pop=nbo emp=gd3bj scrf=(solvent=dichloromethane)\\Benzyne + 2N2 vdw\\0,1\

N,1.5711182866,1.2415529238,-0.9326013299\C,-0.9176042885,-0.4230210242,1.2806528719\ N,1.5548227289,-2.8725550265,0.4323422966\N,1.3166830719,0.4023759494,-1.5787898635\

C,-0.9131707397,0.7825174026,1.5758445041\N,2.4257825528,-2.2185519889,0.4356498448\

C,-1.482975424,-1.011376399,0.1721722117\C,-1.4740884552,1.8213154498,0.8677779205\

C,-2.0884984279,1.326755614,-0.2940394379\C,-2.0922023649,-0.0309990786,-0.627790635\

H,-1.4680390078,2.8724516356,1.1234457634\H,-2.5767267676,2.0307089008,-0.9601931113\

H,-2.5832009917,-0.3433266988,-1.5437709077\H,-1.482349173,-2.0613036601,-0.0885941279\\

Version=ES64L-G16RevB.01\State=1-A\HF=-449.6183016\RMSD=4.633e-09\RMSF=4.626e-06\ Dipole=-0.4626799,0.1824054,-0.7021393\Quadrupole=-0.957811,3.2080731,-2.2502621,-1.9440569,2.4891916,0.1442901\PG=C01 [X(C6H4N4)]\\@

**TS-2d/4d**

HF=-449.5333169 a.u., NIMAG=1, -394.0146 cm^-1^

1\1\GINC-R01N18\FTS\RPBE1PBE\def2TZVP\C6H4N4\WURTHWE\07-Jan-2025\0\\#pbe1pbe/def2tzvp opt=(ts,noeigentest,calcfC,maxstep=8) geom=check freq pop=nbo emp=gd3bj scrf=(solvent=dichloromethane)\\TS for benzotetrazine-to dinitrile 4d\\0,1\

C,-0.0545354547,0.817800232,0.0691748423\C,1.2705526844,1.4292743151,-0.0783316781\

C,2.400918763,0.7193945687,-0.0624581084\C,2.4008853149,-0.7194715717,0.0624377202\

C,1.2704926145,-1.4293101749,0.0782986718\C,-0.0545782246,-0.8177677782,-0.069108466\

H,1.2751561161,2.5081810759,-0.144807537\H,3.3515615489,1.2345228776,-0.1326578976\

H,3.3515073791,-1.2346472643,0.1325762761\H,1.2750626935,-2.5082232811,0.144670033\

N,-1.0308671961,-1.5141540488,-0.3155906336\N,-2.7116479138,-0.5158542646,-0.2234725959\

N,-2.7116561015,0.5159318191,0.2234375955\N,-1.0307822237,1.5141804953,0.3158317778\\

Version=ES64L-G16RevB.01\State=1-A\HF=-449.5333169\RMSD=6.195e-09\RMSF=5.763e-06\Dipole=2.5720607,-0.0000991,-0.000173\Quadrupole=2.5543465,-1.004045,-1.5503015, 0.0000581,-0.000086,-2.1250089\PG=C01 [X(C6H4N4)]\\@

**4d**

HF=-340.213942 a.u., NIMAG=0

1\1\GINC-R02N27\FOpt\RPBE1PBE\def2TZVP\C6H4N2\WURTHWE\13-Jul-2021\0\\# pbe1pbe/ def2tzvp opt freq pop=nbo emp=gd3bj scrf=(solvent=dichloromethane)\\Dinitrile 4d\\0,1\

C,0.201872412,0.,0.0897974836\C,-0.0889237504,0.,1.4779678493\

C,1.4538576379,0.,-0.3960364317\C,2.6372910812,0.,0.4205177562\

C,3.8891973263,0.,-0.0656295794\C,4.1797565859,0.,-1.4538916968\

N,-0.3439708866,0.,2.6024531216\H,-0.6531605855,0.,-0.5756132906\

H,1.5791459815,0.,-1.4738242465\H,2.5122942379,0.,1.4983282642\

H,4.7443846032,0.,0.5996203716\N,4.43526641,0.,-2.5782328267\\

Version=ES64L-G16RevB.01\State=1-A'\HF=-340.213942\RMSD=5.511e-09\RMSF=4.546e-05\Dipole=-0.0000193,0.,-0.0000547\Quadrupole=3.9230725,5.3011061,-9.2241786, 0.,16.1356593,0.\PG=CS[SG(C6H4N2)]\\@

**4d-vdw**

HF=-449.6551137 a.u., NIMAG=0

1\1\GINC-R08N11\FOpt\RPBE1PBE\def2TZVP\C6H4N4\WURTHWE\04-Jun-2025\0\\#

pbe1pbe/def2tzvp opt=(maxstep=9,maxcycle=120) freq pop=nbo emp=gd3bj

scrf=(solvent=dichloromethane)\\Dinitrile 4d N2 vdw\\0,1\

C,-0.2425604411,1.4193948618,0.5159130237\C,0.4580464427,1.3649691376,-0.7166093289\ C,1.633316157,0.7481817387,-0.8902988391\C,2.3722205056,-0.017449979,0.096138276\ C,1.9005961302,-1.0009734881,0.8720908784\C,0.5780603372,-1.5104942789,0.8045962121\ H,0.0072759051,1.9169275639,-1.5333437813\H,2.1203344945,0.8738912381,-1.8528816206\ H,3.4333988472,0.202963991,0.1671716962\H,2.5587843569,-1.5055148217,1.5701295142\

N,-0.4765161144,-1.9733975971,0.7609370526\N,-2.6825177395,-0.0825738361,-1.445005466\

N,-3.4077953262,-0.4412276936,-0.716020382\N,-0.8311225551,1.5089341635,1.5027237647\\ Version=ES64L-G16RevB.01\State=1-A\HF=-449.6551137\RMSD=4.711e-09\RMSF=2.555e-05\Dipole=2.7409039,0.6016892,-1.5608885\Quadrupole=6.4295947,-4.6936883,-1.7359064,-1.8772824,3.4930372,-5.0154448\PG=C01 [X(C6H4N4)]\\@

**5d**

HF=-340.1474921 a.u., NIMAG=0

1\1\GINC-R01N14\FOpt\RPBE1PBE\def2TZVP\C6H4N2\WURTHWE\15-Jul-2021\0\\# pbe1pbe/def2tzvp opt freq pop=nbo emp=gd3bj scrf=(solvent=dichloromethane)\\Four-membered ring 5d at benzene\\0,1\

C,0.0015644939,0.,0.0385009581\C,0.0138479597,0.,1.3894291791\

C,1.1661644268,0.,2.165601746\C,2.4226049334,0.,1.6691180058\

C,2.4536616459,0.,0.2421112111\C,1.3122023056,0.,-0.5266192369\

N,-0.7689124342,0.,2.6678112232\N,0.2748680572,0.,3.3711054463\

H,-0.8861149836,0.,-0.5803284015\H,1.4089283079,0.,-1.6057320179\

H,3.4172555115,0.,-0.2531562829\H,3.329709272,0.,2.2591149103\\

Version=ES64L-G16RevB.01\ State=1-A'\HF=-340.1474921\RMSD=5.488e-09\RMSF=5.508e-05\Dipole=0.9798246, 0.,-1.4546728\Quadrupole=2.2541003,-1.4015898,-0.8525104, 0.,3.8277808,0.\PG=CS[SG(C6H4N2)]\\@

**5d´**

HF=-340.1037595 a.u., NIMAG=0

1\1\GINC-R08N37\FOpt\RPBE1PBE\def2TZVP\C6H4N2\WURTHWE\13-Jul-2021\0\\# pbe1pbe/def2tzvp opt freq pop=nbo emp=gd3bj scrf=(solvent=dichloromethane)\\ \\Four-membered ring 5d´ at benzenel\\0,1\

N,0.5263263245,0.,0.2854166944\C,-0.0529925903,0.,1.4372893078\

C,1.1965191709,0.,2.1969236156\N,1.9524515782,0.,1.1524881342\

C,1.2366863467,0.,3.6332676149\C,0.0033084232,0.,4.1785838211\

C,-1.2630343149,0.,3.4087334258\C,-1.3466218819,0.,2.0627527395\

H,-2.2870017551,0.,1.5264918397\H,-2.1723239778,0.,3.9988513828\

H,-0.1020393312,0.,5.2574178173\H,2.145639679,0.,4.2212260396\\

Version=ES64L-G16RevB.01\ State=1-A'\HF=-340.1037595\RMSD=5.740e-09\RMSF=5.076e-05\Dipole=-1.5200319,0.,2.5000084\ Quadrupole=2.1864346,-0.7105606,-1.475874, 0.,3.532058,0.\PG=CS [SG(C6H4N2)]\\@

**TS-3d/7d**

HF=-340.1347307 a.u., NIMAG=1, -439.2295 cm^-1^

1\1\GINC-R03N27\FTS\RPBE1PBE\def2TZVP\C6H4N2\WURTHWE\16-Dec-2024\0\\#

pbe1pbe/def2tzvp opt=(ts,noeigentest,calcfc,maxstep=2) freq pop=nbo emp=gd3bj scrf=(solvent=dichloromethane)\\TS for formation of benzyne from carbene\\0,1\

C,0.000671378,0.,-0.0003444388\C,0.0007134026,0.,1.390672263\

C,1.1940691582,0.,2.1206215529\C,2.4061743349,0.,1.4487461382\

C,2.2329712609,0.,0.0829463715\C,1.2032952401,0.,-0.7429332013\

N,3.7040418632,0.,-0.7190898511\N,4.4651620798,0.,-1.5035449522\

H,-0.9422456519,0.,1.9289311758\H,-0.9587955021,0.,-0.5130861605\

H,1.1787489135,0.,3.2036454422\H,3.3661260881,0.,1.949357254\\

Version=ES64L-G16RevB.01\State=1-A'\HF=-340.1347307\RMSD=4.132e-09\RMSF=5.726e-05\Dipole=1.2764967,0.,1.1880977\Quadrupole=4.7960376,-3.2473164,-1.5487212,0.,-2.8742711,0.\PG=CS [SG(C6H4N2)]\\@

**TS-3d/7d-vdw**

HF =-449.5843445 a.u., NIMAG=1, -439.2481 cm^-1^

1\1\GINC-R02N05\FTS\RPBE1PBE\def2TZVP\C6H4N4\WURTHWE\20-May-2025\0\\#

pbe1pbe/def2tzvp opt=(ts,noeigentest,calcfc,maxstep=6) freq pop=nbo emp=gd3bj scrf=(solvent=dichloromethane)\\ [\\TS](file:///\\TS) for formation of benzyne from carbene +n2 vdw\\ 0,1\

C,-0.0008571464,0.0028993205,0.0003101467\C,-0.0007405043,0.0028046257,1.3913372971\ C,1.1924377207,0.0006880315,2.1212435387\C,2.4042076504,-0.0032084217,1.4493197564\ C,2.2314055936,-0.0035884166,0.0831690727\C,1.2013291443,-0.0004501844,-0.7425429077\ N,3.7017418527,-0.021390157,-0.7174299141\N,4.4647803753,-0.0428743016,-1.4997519022\ N,1.8390340954,-3.400566148,0.6653943813\N,0.756714865,-3.4762255963,0.7618056417\

H,-0.9437000161,0.0032919885,1.9295095955\H,-0.9604321238,0.0038366434,-0.5121668901\ H,1.1770836139,-0.0002079512,3.2042595074\H,3.3641243235,-0.0077321854,1.9500337657\\

Version=ES64L-G16RevB.01\State=1-A\HF=-449.5843445\RMSD=5.580e-09\RMSF=2.717e-06\Dipole=1.26538,0.0110644,1.1817319\Quadrupole=4.648966,-3.1102152,-1.5387508,1.6178462,-2.706814,1.6523175\PG=C01 [X(C6H4N4)]\\@

**7d**

HF=-340.1427215 a.u., NIMAG=0

1\1\GINC-R01N24\FOpt\RPBE1PBE\def2TZVP\C6H4N2\WURTHWE\16-Dec-2024\0\\# pbe1pbe/ def2tzvp opt freq pop=nbo emp=gd3bj scrf=(solvent=dichloromethane)\\Carbene 7d\\0,1\

C,0.0267242577,0.,0.0040329613\C,0.0365822112,0.,1.4073815299\

C,1.2401378869,0.,2.0627776728\ C,2.3797291728,0.,1.2353610004\

C,2.4926805232,0.,-0.1574151018\C,1.2015327256,0.,-0.7313305976\ N,3.597667629,0.,1.8946208569\ N,4.5853084533,0.,2.3779135955\

H,-0.9311137398,0.,-0.5081198236\H,1.1094489496,0.,-1.8156301953\

H,-0.890895917,0.,1.965902743\H,1.3139027528,0.,3.1437733527\\

Version=ES64L-G16RevB.01\State=1-A'\HF=-340.1427215\RMSD=7.550e-09\RMSF=3.085e-05\ Dipole=-0.7073287,0.,1.9528012\Quadrupole=2.6921099,-2.5136849,-0.1784251,0.,5.0095397,0.\ PG=CS [SG(C6H4N2)]\\@

**7d-vdw**

HF=-449.5923319 a.u., NIMAG=0

1\1\GINC-R03N19\FOpt\RPBE1PBE\def2TZVP\C6H4N4\WURTHWE\19-May-2025\0\\#

pbe1pbe/def2tzvp opt=(maxstep=6) freq pop=nbo emp=gd3bj scrf=(solvent =dichloromethane)\\ Carbene 7d + N2 vdw\\0,1\

C,0.0647180488,-1.8796952904,0.202048629\C,-1.1810552854,-1.3323670738,-0.1403543778\

C,-1.2619998273,0.0051216111,-0.4277697697\C,-0.0484216681,0.715534454,-0.3490404257\ C,1.2393026594,0.2828949915,-0.022277604\C,1.2107093336,-1.1020941734,0.2555397967\

N,-0.1272252425,2.0685705648,-0.6338752813\N,-0.1443216076,3.1464237043,-0.8504467601\ H,0.1208852294,-2.9400650641,0.4304466033\H,2.1377069851,-1.6009013455,0.5310564851\

H,-2.0663561691,-1.9545600856,-0.1755577656\H,-2.1949127079,0.486964318,-0.6958088613\

N,-0.9950568272,0.6680256555,2.9987449017\N,-0.3599899213,-0.1212142665,3.3990244298\\ Version=ES64L-G16RevB.01\State=1-A\HF=-449.5923319\RMSD=3.786e-09\RMSF=7.667e-06\Dipole=-2.0149025,0.1033841,-0.4097835\Quadrupole=-4.4439418,5.3268294,-0.8828877,-1.4672519,2.6990622,-1.8935502\PG=C01 [X(C6H4N4)]\\@

**TS-1d/8d**

HF=-449.554487 a.u., NIMAG=1, -477.8893 cm^-1^

1\1\GINC-R01N07\FTS\RPBE1PBE\def2TZVP\C6H4N4\WURTHWE\04-Mar-2025\0\\#pbe1pbe/def2tzvp opt=(ts,noeigentest,calcfC,maxstep=8) freq pop=nbo emp=gd3bj scrf= (solvent=dichloromethane)\\TS for formation of five-membered ring from bisdiazoethane\\0,1\

N,-1.0192955851,1.6573993515,0.1500649895\C,-0.2115311815,-0.559026507,0.0394191103\

N,-1.4868244345,-0.9881380087,-0.103668188\N,-2.1102221217,1.6585536013,-0.2498031284\

C,0.0682185394,0.824690891,0.0142276827\N,-2.3944628193,-1.6298499694,0.1447746309\

C,0.8570305652,-1.4878035537,0.0447885259\C,1.3866878073,1.2829088106,-0.0063061601\

C,2.4089667447,0.3716687107,-0.0582792742\C,2.1374860898,-1.0161871261,-0.0043028214\

H,1.5714122631,2.3505007622,-0.0067123089\H,3.433179143,0.7145015631,-0.1297343619\

H,2.9613362654,-1.7185724618,0.0356524874\H,0.6405287242,-2.5459110636,0.1296888161\\

Version=ES64L-G16RevB.01\State=1-A\HF=-449.554487\RMSD=6.023e-09\RMSF=3.982e-05\Dipole=1.9861567,-0.4330283,0.0673298\Quadrupole=-1.893492,3.0212939,-1.1278019,1.1413473,-0.6265099,0.0491669\PG=C01 [X(C6H4N4)]\\@

**8d**

HF=-449. 5874507 a.u., NIMAG=0

1\1\GINC-R08N20\FTS\RPBE1PBE\def2TZVP\C6H4N4\WURTHWE\10-Feb-2025\0\\#pbe1pbe/def2tzvp opt=(ts,noeigentest,calcfC,maxstep=2) freq pop=nbo em

p=gd3bj scrf=(solvent=dichloromethane)\\Five-membered ring 8d at benzol\\0,1\

N,1.016944733,-1.7219224515,0.0024955589\C,0.177126624,0.4667926231,-0.0064817443\

N,1.6065808683,0.6383764014,0.0022238565\N,2.0631564332,-1.1434696038,0.0124246056\

C,-0.1535503269,-0.8589617209,-0.0058122614\N,2.3229568212,1.5431061358,-0.0058292311\

C,-0.7713770528,1.4628521969,-0.0009821113\C,-1.4637198297,-1.2798020104,-0.0040141396\

C,-2.4433607403,-0.2910839443,0.0000406501\C,-2.1024453682,1.059222761,0.0016485566\

H,-1.7118296267,-2.3341711748,-0.0048867156\H,-3.4884309324,-0.5757252818,0.0035471346\

H,-2.8857483314,1.8074192313,0.0057882383\H,-0.4811532714,2.507346838,-0.0001923972\\

Version=ES64L-G16RevB.01\State=1-A\HF=-449.5874507\RMSD=7.131e-09\RMSF=1.864e-04\Dipole=-2.6282299,0.2283552,-0.0003373\Quadrupole=-1.4310133,0.7022299,0.7287834,-0.6845921,-0.0327711,0.0457385\PG=C01 [X(C6H4N4)]\\@

Table S5 Species e R=Ph**:** Total energies (E_tot_) and Gibbs free energies (G_298_) [a.u.] of compounds **e** and of the related transition states as given in the Gaussian archive entries. The relative energies [kcal/mol] include all involved species according to the respective reactions as given in the schemes in the manuscript. Van der Waals energies are given in italics.

| **Species e**  **R=Ph** | **E_tot_ [a.u.]** | **E_rel_ [kcal/mol]** | **G_298_ [a.u.]** | **E_rel_ [kcal/mol]** |
| --- | --- | --- | --- | --- |
| **N_2_** | -109.44698 |  | -109.45973 |  |
| **1e** | -757.83299 | 11.47 | -757.66667 | 0.00 |
| **TS -1e/2e** | -757.81676 | 21.66 | -757.64787 | 11.80 |
| **2e** | -757.85127 | 0.00 | -757.67867 | -7.53 |
| **TS-2e/3e** | -757.80050 | 31.86 | -757.63664 | 18.84 |
| **3e (tolane)** | -539.02423 |  | -538.87183 |  |
| **3e+2N_2_** | -757.91818 | -41.99 | -757.79129 | -78.20 |
| **3e-vdw** | *-757.92392* | *-45.51* | *-757.77871* | *-70.31* |
| **TS-2e/4e** | -757.78594 | 41.00 | -757.62057 | 28.93 |
| **4e (benzonitrile)** | -757.91774 | -41.71 | -757.79155 | -78.36 |
| **4e-vdw** | *-757.92691* | *-47.47* | *-757.77463* | *-67.75* |
| **6e** | -757.72459 | 79.50 | -757.56043 | 66.66 |
| **TS-1e/7e** | *-757.79281* | *36.68* | *-757.63049* | *22.70* |
| **TS-3e/7e** | -648.36846 |  | -648.21270 |  |
| **TS-3e/7e+N_2_** | -757.81544 | 22.49 | -757.67243 | -3.62 |
| **TS-3e/7e-vdw** | *-757.81895* | *20.28* | *-757.66598* | *0.43* |
| **7e** | -648.37121 |  | -648.21238 |  |
| **7e+N_2_** | -757.81818 | 20.76 | -757.67211 | -3.41 |
| **7e-vdw** | *-757.82108* | *18.94* | *-757.66613* | *0.34* |
| **TS-1e/8e** | -757.71294 | 86.81 | -757.54482 | 76.46 |
| **8e** | -757.80592 | 28.46 | -757.63672 | 18.79 |

**Gaussian Archive Entries**

(Total energies (a.u.), number of imaginary frequencies (for transition states: imaginary frequencies), coordinates)

**1e**

HF=-757.8329937 a.u., NIMAG=0

1\1\GINC-R09N07\FOpt\RPBE1PBE\def2TZVP\C14H10N4\WURTHWE\24-May-2021\0\ \# pbe1pbe/def2tzvp opt=(maxstep=6) freq pop=nbo emp=gd3bj scrf=(solvent=dichloromethane)\\Bis-diazoethane with 2 phenyl groups\\0,1\

C,-0.0942198603,-0.0377445519,-0.0478322348\C,-0.11850887,0.0037033879,1.3489873463\

C,1.0970227797,0.0646373748,2.038733139\C,2.2960136017,0.0793762032,1.3474799538\

C,2.3126960183,0.0376210991,-0.0407250228\C,1.1101377475,-0.0200262071,-0.7312428982\

C,-1.4049549372,-0.0083750225,2.0347512521\N,-1.4249039199,0.0010591754,3.3395032587\

N,-1.4389015757,-0.0036845165,4.4659567313\C,-2.7125783399,0.0057190143,1.3742572723\

N,-3.3323385753,-1.1409520029,1.311722998\N,-3.8756450629,-2.1268397122,1.2674667104\

C,-3.333413674,1.1769480343,0.7675081724\C,-2.6861603661,2.4122081361,0.8602984653\

C,-3.2442274627,3.5434426837,0.2887128726\C,-4.4558390421,3.4697364239,-0.3842283548\

C,-5.1044252017,2.2453242224,-0.4805868845\C,-4.5528761947,1.1090631966,0.0853920195\

H,1.1065210502,-0.0521201757,-1.8148359353\H,3.2535473838,0.0505077557,-0.577665709\

H,3.227212058,0.1247430043,1.9006462344\H,1.1100301549,0.1012313645,3.1224557652\

H,-1.0281624068,-0.0833547219,-0.59566554\H,-2.7252930371,4.491623529,0.3716415842\

H,-1.739904421,2.4796821079,1.3842231544\H,-5.0768814511,0.1640663678,-0.0060561547\

H,-6.0513502271,2.1720270889,-1.0032169491\H,-4.8907359101,4.35631841,-0.8297726179\\

Version=ES64L-G16RevB.01\State=1-A\HF=-757.8329937\RMSD= 6.929e-09\RMSF=2.321e-05\Dipole=0.2191292,0.3895226,-0.4255021\Quadrupole=5.2625428,-2.9691664,-2.2933764,-1.6519672,2.9784255,-0.4033599\PG=C01 [X(C14H10N4)]\\@

**TS-1e/2e**

HF=-757.8167577 a.u., NIMAG=1, -263.5720 cm^-1^

1\1\GINC-R08N19\FTS\RPBE1PBE\def2TZVP\C14H10N4\WURTHWE\24-May-2021\0\\# pbe1pbe/def2tzvp opt=(ts,noeigentest,calcfC,maxstep=6) freq pop=nbo emp=gd3bj scrf=(solvent=dichloromethane)\\TS for formation of tetrazine 2e\\0,1\

C,-0.0000523426,-0.0008951743,0.0024378796\C,-0.000872061,0.0003913662,1.399748053\

C,1.2203341844,0.0000541172,2.078498705\C,2.4111324997,0.0024704776,1.3731343865\

C,2.4062130653,0.0075158454,-0.0157614185\C,1.195107912,0.0085075644,-0.6955406054\

C,-1.2403043944,0.0148901024,2.1510539902\N,-2.3931643491,0.3618350654,1.5370052726\

N,-3.4342083928,0.8412293523,1.6729091292\C,-1.4981003627,-0.2222783652,3.5155311616\

N,-2.3474689594,0.7228017686,3.9758652044\N,-3.1906142147,1.4593169497,3.695308218\

C,-1.0626487584,-1.2886009483,4.3954193249\C,-1.2119939242,-1.1914700071,5.7813267786\

C,-0.8104633102,-2.2312414925,6.6018869095\C,-0.2442888427,-3.3771519982,6.058553014\

C,-0.0877736007,-3.4762794312,4.68205093\C,-0.4930756674,-2.44387615,3.8541504922\

H,3.3509961079,0.0049941174,1.9129346462\H,3.340166781,0.0082445706,-0.564963619\

H,1.1805882113,0.0077995234,-1.779323676\H,-0.9416527251,-0.0252652349,-0.5347592846\

H,1.2297080826,0.0099379669,3.162031293\H,0.3499348846,-4.3677693383,4.2480102964\

H,-0.3812642399,-2.5319598312,2.7799216662\H,-1.6303033361,-0.2895312414,6.2141200329\

H,-0.9327177012,-2.1422733809,7.6751695041\H,0.0750848492,-4.1866721911,6.7039759213\\

Version=ES64L-G16RevB.01\State=1-A\HF=-757.8167577\RMSD=3.346e-09\RMSF=1.277e-05\Dipole=1.2149301,-0.7840074,0.0932787\Quadrupole=-5.4387727,-3.3568496,8.7956223, 2.7197787,-2.3227641,-1.6758627\PG=C01 [X(C14H10N4)]\\@

**2e**

HF=-757.8512698 a.u., NIMAG=0

1\1\GINC-R09N07\FOpt\RPBE1PBE\def2TZVP\C14H10N4\WURTHWE\24-May-2021\0\

\# pbe1pbe/def2tzvp opt=(maxstep=5,gdiis) freq pop=nbo emp=gd3bj scrf=

(solvent=dichloromethane)\\Tetrazine 5e\\0,1\

N,-2.1277453091,0.8517330572,-0.5383790811\N,-2.9949877016,0.0914916493,-1.1517707689\

N,-2.63772348,-1.0726829115,-1.6257811095\N,-1.5098450739,-1.6198892733,-1.2586554906\

C,-0.6504773394,-0.9571558942,-0.4930584755\C,-0.9017780197,0.4116676937,-0.2791226278\

C,0.5564525136,3.4017018907,1.4422755121\C,-0.351603464,2.450678579,1.0088788198\

C,0.0715578953,1.3988819491,0.1970540575\C,1.4089796778,1.3243508562,-0.1927081575\

C,2.308702763,2.2871660883,0.2287339492\C,1.8867152009,3.3222151313,1.0532839055\

C,0 .9506780658,-1.4521324546,1.3430675133\C,0.4717018977,-1.720615254,0.0606224634\

C,1.0355331239,-2.7573917342,-0.6820685921\C,2.0790597842,-3.5000288985,-0.1563444792\

C,2.553527166,-3.2278363173,1.1195956989\C,1.9818677877,-2.2087686174,1.8705878659\

H,0.2249021494,4.2082635826,2.0852297694\H,-1.3920554412,2.5067645971,1.3031257714\

H,1.7421191258,0.5202508125,-0.8372292951\H,3.3433587403,2.2302532991,-0.0873168607\

H,2.5958024866,4.0691026966,1.3905340186\H,0.5066377943,-0.6598739063,1.9330852306\

H,0.6555519904,-2.9651490231,-1.6743984129\H,2.5232379532,-4.2945646531,-0.7439466173\

H,3.3681975347,-3.8120200431,1.5312084001\H,2.3411471784,-2.003013902,2.8716989924\\

Version=ES64L-G16RevB.01\State= 1-A\HF=-757.8512698\RMSD=3.933e-09\RMSF=1.752e-05\Dipole=2.9333447,0.3 132168,1.4416148\Quadrupole=-8.1712725,6.954933,1.2163395,-3.1467767,- 6.6293256,2.3356357\PG=C01 [X(C14H10N4)]\\@

**TS-2e/3e**

HF=-757.8004965 a.u., NIMAG=1, -508.1147 cm^-1^

1\1\GINC-R07N10\FTS\RPBE1PBE\def2TZVP\C14H10N4\WURTHWE\26-May-2021\0\\

# pbe1pbe/def2tzvp opt=(ts,noeigentest,calcfC,maxstep=6) freq pop=nbo emp=gd3bj scrf=(solvent=dichloromethane)\\TS for tolane formation from tetrazine 2e\\0,1\

C,0.0545233501,0.2315516612,-0.031172339\C,-0.0156555499,0.0498053357,1.3552072032\

C,1.1587509461,-0.1998924365,2.0663489016\C,2.3749967517,-0.2625566781,1.4039407815\

C,2.4386138743,-0.0782663454,0.0307593758\C,1.271886112,0.169296913,-0.6832698807\

C,-1.3106651788,0.1217265428,1.9871880033\N,-1.0664671731,0.5789474793,3.6022563843\

N,-1.7979263764,1.0034939006,4.3565697226\C,-2.5149323904,-0.2148146711,1.7022994173\

C,-3.1756716442,-1.06328865,0.7404833724\C,-2.5469105443,-2.2583756601,0.3710312505\

C,-3.1387274125,-3.1039886389,-0.5486146767\C,-4.3628252823,-2.7742916305,-1.1189392593\

C,-4.9889228148,-1.5900534358,-0.7594897454\C,-4.403610905,-0.7370055147,0.1633823179\

N,-3.7100516943,0.7084061816,2.4757539019\N,-3.6668445804,1.2827703784,3.4516960962\

H,3.2789324553,-0.4636043008,1.9671792565\H,1.1153274573,-0.3607656067,3.1356293783\

H,-0.8541012425,0.430830017,-0.5868099212\H,1.3118563517,0.318752027,-1.7560028073\

H,3.3913919303,-0.1289632222,-0.4828562252\H,-1.5958496874,-2.5184434925,0.8203500579\

H,-4.890638849,0.1928746835,0.4264988887\H,-5.9398133799,-1.3217063094,-1.205086538\

H,-4.8236787583,-3.4375414381,-1.8413452672\H,-2.6436037175,-4.0291398878,-0.8199814478

\\Version=ES64L-G16RevB.01\State=1-A\HF=-757.8004965\RMSD=7.143e-09\RMSF=1.084e-05\Dipole=0.4834385,-0.7009027,-1.2133737\Quadrupole=5.8580393,-2.4940945,-3.3639448, 0.6262247,4.4533775,-1.917841\PG=C01[X(C14H10N4)]\\@

**3e (Tolane)**

HF=-539.024229 a.u., NIMAG=0

1\1\GINC-R02N20\FOpt\RPBE1PBE\def2TZVP\C14H10\WURTHWE\12-Nov-2024\0\\#

pbe1pbe/def2tzvp opt freq pop=nbo emp=gd3bj scrf=(solvent=dichloromethane)\\tolane\\0,1\

C,-0.0240923268,-0.0255810891,0.0245477731\C,-0.0170856328,-0.0468520482,1.4242343164\ C,1.2092266784,-0.0181968489,2.098737819\C,2.3954291041,0.0304537369,1.3875199232\ C,2.3794964347,0.0512719377,-0.0011312015\C,1.1671384732,0.0231251206,-0.6782105892\

C,-1.236126862,-0.0967065137,2.1492464878\C,-2.2745283774,-0.1392164479,2.7669416337\

C,-3.493531463,-0.1890976951,3.4919954405\C,-3.4865373037,-0.205936053,4.891754836\

C,-4.6777014272,-0.254566518,5.5946285998\C,-5.8899901685,-0.2870696925,4.9176052146\

C,-5.9058948459,-0.2706283935,3.5288993059\C,-4.7197526925,-0.2220380234,2.817555286\

H,-0.9712674613,-0.0476185917,-0.5006927212\H,1.2183925571,-0.0345195079,3.1818603339\ H,3.3389483639,0.0522837227,1.9202799506\H,3.3102869264,0.089383162,-0.5547173716\ H,1.1496228939,0.0392332317,-1.7617107532\H,-6.8493397764,-0.2958406549,2.9961536542\

H,-4.7289534489,-0.2091039868,1.7343861837\H,-2.5394280177,-0.1805046859,5.4169583488\

H,-4.6601758476,-0.2672016006,6.6781758373\H,-6.8207307543,-0.3251153848,5.4712746103\\ Version=ES64L-G16RevB.01\State=1-A\HF=-539.024229\RMSD=4.746e-09\RMSF=4.437e-06\Dipole=0.0000284,0.0000574,0.0000365\Quadrupole=8.0067271,-12.056113,4.0493859, 0.7075635,-3.6473954,-0.3418097\PG=C01 [X(C14H10)]\\@

**3e-vdw**

HF=-757.9237923 a.u., NIMAG=0

1\1\GINC-R02N34\FOpt\RPBE1PBE\def2TZVP\C14H10N4\WURTHWE\17-May-2025\0\

\# pbe1pbe/def2tzvp opt=(maxstep=6) freq pop=nbo emp=gd3bj scrf=(solvent=dichloromethane) \\Tolane + 2 N2 vdw\\0,1\

C,-2.7194730537,1.3781469825,0.2669715563\C,-2.0322259431,0.2235300576,0.6594527355\

C,-2.7621822491,-0.925330805,0.9862452043\C,-4.1444803029,-0.9149791006,0.9210551205\

C,-4.8187670721,0.2348591772,0.5307266778\C,-4.1019581453,1.3789310294,0.2044787744\

C,-0.6148838274,0.2134880499,0.7106449266\N,-0.0257609599,1.678602612,-2.4869472085\ N,0.7167919234,0.9362483645,-2.7763853188\C,0.5933796968,0.1944084543,0.722836574\ N,0.048922743,-2.7009490863,-1.3014568848\N,-0.6496861697,-2.1764070993,-1.9518705791\ C,2.0111193068,0.1639449154,0.6977545451\C,2.6988820853,-1.0329679848,0.9297614763\ C,4.0819111964,-1.0612187017,0.8889557695\C,4.7987247345,0.0971324446,0.6178586813\ C,4.1239179736,1.2889380812,0.3866961083\C,2.7411219499,1.3268328639,0.4251242647\

H,-4.6996979987,-1.8101278327,1.1759536479\H,-5.90123359,0.2391809819,0.4803904347\

H,-4.6239527329,2.2780199075,-0.1016115383\H,-2.1568095071,2.2675228347,0.0105661173\

H,-2.2328682087,-1.8208455791,1.288582351\H,4.6791581035,2.1951097179,0.1743512394\

H,2.2113801477,2.2541338548,0.2434304331\H,2.1363047307,-1.9349050215,1.1379234278\ H,4.6042593601,-1.9935635931,1.0689978396\H,5.881598809,0.0710924747,0.5863926244\\ Version=ES64L-G16RevB.01\State=1-A\HF=-757.9237923\RMSD=3.885e-09\RMSF=6.728e-06\Dipole=-0.0000733,-0.0137917,-0.0506168\Quadrupole=10.0540301,0.9293903,-10.9834204, 0.7725939,-0.0794114,-3.3244864\PG=C01 [X(C14H10N4)]\\@

**TS-2e/4e**

HF=-757.7859361 a.u., NIMAG=1, -407.7929 cm^-1^

1\1\GINC-R01N03\FTS\RPBE1PBE\def2TZVP\C14H10N4\WURTHWE\04-Jun-2021\0\\

# pbe1pbe/def2tzvp opt=(ts,noeigentest,calcfC,maxstep=6) freq pop=nbo emp=gd3bj scrf=(solvent=dichloromethane)\\TS for formation of 2 benzonitriles + N2\\0,1\

C,-0.3718410789,0.2944831633,-0.0305819376\C,-0.2168228081,-0.202525402,1.2589360232\

C,1.0492175416,-0.5134340352,1.742406312\C,2.1557390045,-0.348633929,0.9255510897\

C,2.0041610345,0.1455543063,-0.3627580248\C,0.7412816394,0.4741858809,-0.8355234111\

C,-1.4050894231,-0.3429093773,2.1438471346\N,-1.3805691971,0.0759711671,3.2946347604\

N,-3.128892039,0.1856083319,4.0327297004\N,-4.015597024,0.145670521,3.3301811475\

N,-3.8407079824,-0.8198141662,1.7020706256\C,-2.6658950766,-1.0439774763,1.4376180946\

C,-2.2983384475,-2.0541522457,0.4084416566\C,-1.3270145664,-3.0076161103,0.693643311\

C,-1.022664229,-3.9797587656,-0.2452794659\C,-1.6733311585,-3.9932805702,-1.4711751577\

C,-2.6437090139,-3.0412362462,-1.7520951087\C,-2.9662604787,-2.0775128677,-0.8108190655\

H,3.1402637179,-0.6049455311,1.2983395594\H,1.1575521991,-0.8960323255,2.7497288816\

H,-1.3568279637,0.5455687718,-0.4054787641\H,0.6191763096,0.8671443387,-1.8377414579\

H,2.8713678119,0.2753444787,-0.9993293371\H,-0.8120820101,-2.9959852707,1.6467965183\

H,-3.7235256108,-1.3320507146,-1.0197512032\H,-3.1545946054,-3.0496301647,-2.7075433084\

H,-1.423940315,-4.7473048202,-2.208309917\H,-0.269918122,-4.7253572905,-0.0189537753\\

Version=ES64L-G16RevB.01\State=1-A\HF=-757.7859361\RMSD

=4.928e-09\RMSF=1.450e-05\Dipole=1.710345,-0.956116,-2.1041838\Quadrup

ole=-0.7460479,1.6629978,-0.9169498,-0.235443,5.2648771,-4.1693107\PG=

C01 [X(C14H10N4)]\\@

Ph-CN

**4e (Benzonitrile)**

HF=-324.235341 a.u., NIMAG=0

1\1\GINC-R02N38\FOpt\RPBE1PBE\def2TZVP\C7H5N1\WURTHWE\13-Nov-2024\0\\#

pbe1pbe/def2tzvp opt freq pop=nbo emp=gd3bj scrf=(solvent= dichloromethane)\\benzonitrile\\0,1\

C,0.1016731078,0.0818140819,0.\C,-1.1379569071,-0.5590094582,0.\

C,-1.1875634472,-1.9417963136,0.\C,-0.0124907492,-2.6816785093,0.\

C,1.2196764617,-2.0412831593,0.\C,1.2842684685,-0.6591695854,0.\ C,0.1604302235,1.5068650727,0.\N,0.2080433324,2.6582873657,0.\

H,-2.0475417529,0.0282808135,0.\H,-2.1470478633,-2.4442732863,0.\

H,-0.0575102431,-3.764298612,0.\H,2.1346862445,-2.6207992425,0.\

H,2.2393901245,-0.1492831673,0.\\

Version=ES64L-G16RevB.01\State=1-A'\HF=-324.235381\RMSD=9.933e-09\RMSF=4.688e-05\Dipole=-0.0946731,-2.2934882,0.\Quadrupole=7.4600648,-7.0294764,-0.4305884,-0.5991909,0.,0.\PG=CS [SG(C7H5N1)]\\@

2 PhCN + N_2_

**4e-vdw**

HF=-757.9269121 a.u., NIMAG=0

1\1\GINC-R01N04\FOpt\RPBE1PBE\def2TZVP\C14H10N4\WURTHWE\05-Jun-2021\0\

\# pbe1pbe/def2tzvp opt freq pop=nbo emp=gd3bj scrf=(solvent=dichloromethane)\\2 Benzonitrile + N2 vdw\\0,1\

C,0.2529384463,-0.5068991757,1.3067824495\C,1.2076621474,0.4352394676,1.6905720328\

C,2.1808430026,0.8680203208,0.7887991283\C,2.1909138303,0.3570427876,-0.4971236751\ C,1.2396759085,-0.5789481714,-0.8828253934\C,0.2743130825,-1.009469708,0.0177485788\ C,1.1803826111,0.9735005651,3.0105352023\N,1.1545234781,1.4145649111,4.0746856101\

C,-3.6668917025,5.2832913424,1.5265838768\N,-4.4273914013,6.010854462,1.9959763995\

C,-2.7271537436,4.3815815106,0.9462294873\C,-3.1698766979,3.3948450458,0.0645509885\

C,-2.2537295412,2.5176617491,-0.4881245645\C,-0.9066936017,2.6230465669,-0.1687526669\

C,-0.4694575863,3.6054793868,0.70911978\C,-1.3741780495,4.4878516524,1.2717614421\

N,-2.4076322852,1.9940873742,3.4748351477\N,-2.5752123218,1.1349985609,2.826419857\

H,0.5813276917,3.6826396127,0.9601372368\H,-1.044856831,5.2541496105,1.9619436411\

H,-4.2229046498,3.3211334349,-0.1760592159\H,-2.5920962985,1.7471385376,-1.1700126327\

H,-0.1939509949,1.9323961629,-0.6028417701\H,2.9153767179,1.600159853,1.0998168652\

H,-0.4973447266,-0.8334978922,2.0152414121\H,-0.4664895732,-1.738952407,-0.2855113058\

H,1.2507227134,-0.9741728431,-1.8915879192\H,2.9426478769,0.6915389931,-1.2013562491\\ Version=ES64L-G16RevB.01\State=1-A\HF=-757.9269121\RMSD=1.801e-09\RMSF=6.179e-06\Dipole=1.5979312,-2.3580573,-2.993198\Quadrupole=1.0743602,-1.5246096,0.4502494, 15.1365145,-3.1488503,1.5724973\PG=C01 [X(C14H10N4)]\\@

**TS-1e/7e**

HF=-757.7928146 a.u., NIMAG=1, -475.2673 cm^-1^

1\1\GINC-R01N07\FTS\RPBE1PBE\def2TZVP\C14H10N4\WURTHWE\25-May-2021\0\\

# pbe1pbe/def2tzvp opt=(ts,noeigentest,calcfC,maxstep=6) freq pop=nbo

emp=gd3bj scrf=(solvent=dichloromethane)\\TS for N2-elimination from bis(diazo)alkane 1e to give carbene 7e\\0,1\

C,0.0047243986,-0.000011737,0.0022083101\C,0.0039752364,0.0001790163,1.4084811369\

C,1.2452944869,-0.0031264493,2.0579246743\C,2.4259876224,-0.0277182367,1.3358852891\

C,2.4132151913,-0.0467794008,-0.0524565099\C,1.190099288,-0.0328772137,-0.7099006494\

C,-1.2799422384,-0.1167667513,2.0595476866\C,-2.3609218869,0.7033184673,1.7300974887\

N,-3.529554978,0.1543512198,2.0993323269\N,-4.4787836356,-0.308843696,2.4500212772\

C,-2.4419245869,2.0486258963,1.1389403112\C,-3.5810646065,2.493870142,0.4673186503\

C,-3.6207642399,3.7662521075,-0.0792132145\C,-2.5226123101,4.6084506249,0.0266123955\

C,-1.38525748,4.1690196305,0.6904795042\C,-1.3431010032,2.9014222112,1.2466942257\

N,-1.0014939148,0.0393505349,3.8189793915\N,-1.3895708995,-0.3042153999,4.7964093173\

H,-0.5235157805,4.8200485911,0.7818274307\H,-2.552936786,5.6013183517,-0.4061727565\

H,-4.5131634157,4.0978233079,-0.5972623081\H,-4.4417480047,1.8423566532,0.3602712272\

H,-0.4553564066,2.5663824091,1.7683074536\H,1.1548785271,-0.0383011666,-1.7937644266\

H,-0.9406323051,0.0087682801,-0.5280377178\H,1.2879835295,0.0405489842,3.1395028628\

H,3.3702017388,-0.021607905,1.8692567921\H,3.3405916021,-0.0607554795,-0.6121873798

\\Version=ES64L-G16RevB.01\State=1-A\HF=-757.7928146\RMSD=6.522e-09\RMSF=6.232e-07\Dipole=-0.5058009,0.8798329,-0.3696383\Quadrupole=4.9351647,-3.1271574,-1.8080073,-0.2598329,3.507568,-0.4995529\PG=C01 [X(C14H10N4)]\\@

**TS-3e/7e**

HF=-648.3684613 a.u., NIMAG=1, -407.8133 cm^-1^

1\1\GINC-R07N17\FTS\RPBE1PBE\def2TZVP\C14H10N2\WURTHWE\22-Jun-2021\0\\

# pbe1pbe/def2tzvp opt=(ts,noeigentest,calcfc,maxstep=5) freq pop=nbo emp=gd3bj scrf=(solvent=dichloromethane)\\TS for N2 elimination to give tolane\\0,1\

C,0.0512348806,0.5862441721,0.0495571326\C,0.0337130621,-0.0899162168,1.2678103007\ C,1.239128766,-0.5395184285,1.8120127975\C,2.4310210199,-0.3138217446,1.1462924422\ C,2.4426215847,0.3596607018,-0.0684126635\C,1.2486416752,0.807788586,-0.6127760622\ C,-1.1914692644,-0.3516844297,2.0229197756\N,-2.4183396971,0.0992295864,1.2113668391\

N,-3.4251383147,0.3349354537,0.8408630293\C,-1.6151456185,-0.8410042765,3.1509913445\

C,-0.8476411372,-1.3722935825,4.236687205\C,-0.3392353768,-0.5323012502,5.2408534838\ C,0.3898360464,-1.0542295722,6.2957977601\C,0.6311746067,-2.419011636,6.3904709385\ C,0.1205704704,-3.2590320318,5.4091180117\C,-0.6167500906,-2.7523359287,4.3521148832\

H,3.3585834019,-0.6691126651,1.5797795638\H,3.3776671882,0.5337926579,-0.5873407087\ H,1.2452155595,1.3355893747,-1.5591771374\H,-0.8683135716,0.9507925961,-0.3946964934\ H,1.2333803729,-1.0659099178,2.7578484437\H,0.2934036231,-4.3283573506,5.4672186815\

H,-1.0159338964,-3.421809658,3.5987852839\H,-0.5185827006,0.5351046906,5.1808830226\ H,0.7759233014,-0.3815455143,7.0542056487\H,1.2012550537,-2.8218033646,7.2189288307\\

Version=ES64L-G16RevB.01\State=1-A\HF=-648.3684613\RMSD=7.790e-09\RMSF=1.752e-06\Dipole=0.386086,0.7099695,-1.603426\Quadrupole=-1.0185958,-0.5568121,1.5754079,-2.2365811,4.7510332,-2.873321\PG=C01 [X(C14H10N2)]\\@

**TS-3e/7e-vdw**

HF=-757.8189461 a.u., NIMAG=1, -430.7124 cm^-1^

1\1\GINC-R03N10\FTS\RPBE1PBE\def2TZVP\C14H10N4\WURTHWE\20-May-2025\0\\

# pbe1pbe/def2tzvp opt=(ts,noeigentest,calcfc,maxstep=6) freq pop=nbo emp=gd3bj scrf=(solvent=dichloromethane)\\TS for N2-elimination to give tolane vdw\\0,1\

C,0.8126303717,-1.4272291779,0.0391513376\C,1.422318484,-0.2817862484,-0.4760673566\ C,2.77203503,-0.0552432923,-0.2151701834\C,3.4981425955,-0.9538389475,0.5504011182\ C,2.8862466059,-2.0859783939,1.067016587\C,1.5417048367,-2.3180290796,0.8068128736\ C,0.5884130602,0.6473196799,-1.2360795517\N,1.4860011141,1.6283709143,-2.0299594741\

N,1.8908978352,2.4260220546,-2.6668360623\C,-0.6526770979,0.94302618,-1.4822912436\ C,-1.823357974,0.3324253846,-0.9234993374\C,-2.6065228404,-0.5573547564,-1.6742433943\

C,-3.7348176991,-1.1427097092,-1.1238872916\C,-4.1364466307,-0.8379559802,0.1702396767\

C,-3.3828721029,0.0633978514,0.9119405039\C,-2.2417293308,0.6399875052,0.381590347\ N,0.9315726697,0.2648438859,3.213281055\N,0.9873203624,1.1590863789,2.5938135702\

H,1.0561498728,-3.2020951265,1.2030802823\H,3.4539794293,-2.7855002981,1.668887265\ H,4.5461563492,-0.7623018014,0.7484285289\H,3.2662917423,0.8313601844,-0.5966795383\

H,-0.2356235926,-1.6085777359,-0.1612700644\H,-4.3131968732,-1.8395936552,-1.7209262741\ H,-2.3142811108,-0.7921300725,-2.6913615813\H,-1.6593364868,1.3357524656,0.9746531733\

H,-3.6825430001,0.3177955127,1.9229951051\H,-5.0264786195,-1.2896067225,0.5915749294\\ Version=ES64L-G16RevB.01\State=1-A\HF=-757.8189461\RMSD=3.866e-09\RMSF=1.401e-06\Dipole=1.7488043,-0.2868388,0.2277558\Quadrupole=2.8650102,-1.262151,-1.6028592, 5.1629504,-2.8382361,-0.1553328\PG=C01 [X(C14H10N4)]\\@

**6e**

1\1\GINC-R02N14\FOpt\RPBE1PBE\def2TZVP\C14H10N4\WURTHWE\23-May-2021\0\

\# pbe1pbe/def2tzvp opt=(maxstep=5,gdiis) geom=check guess=read freq pop=nbo emp=gd3bj scrf=(solvent=dichloromethane)\\ Bis-(nitrile-imine \0,1\

N,-1.6817272679,-2.2852517936,-1.7127632114\C,-1.8755321514,-1.1121830366,-1.5457782729\ N,-1.3784739159,-3.4387132718,-2.0066730822\N,0.0696746056,-3.667027102,-2.0526668336\ N,0.7251843093,-2.6340050292,-1.9000181551\C,1.2834639387,-1.5774279575,-1.8889605102\

C,-1.9223844609,1.8975872075,0.6351169175\C,-2.1390861715,1.3305842585,1.8831583771\

C,-2.3028519606,-0.0445839297,2.0093207727\C,-2.2341026663,-0.8553576739,0.8920474597

\C,-2.0169098639,-0.2887536516,-0.3691654586\C,-1.8746646197,1.0934344834,-0.4913114737\ C,2.9134077207,1.3997488689,-0.3694394526\C,2.5054406564,0.4632410482,-1.303960794\ C,1.7640537314,-0.6480859882,-0.9010920154\C,1.4289735976,-0.8070008279,0.4493384899\ C,1.8266578414,0.1395303977,1.3741366385\C,2.569942368,1.2413680592,0.9663127964\

H,-1.7952150998,2.9690854391,0.5390910289\H,-2.1828326957,1.9615312291,2.7632389552\

H,-2.4742171165,-0.4840737831,2.9848420753\H,-2.3443546603,-1.9296514254,0.9839909726\

H,-1.7094642755,1.5211364045,-1.4725552847\H,3.4912679136,2.2602738092,-0.6840682166\ H,2.7489621907,0.5823604606,-2.3525725719\H,0.8480054141,-1.667158256,0.7591173538\

H,1.5560446112,0.0210958926,2.4165390563\H,2.8797990274,1.9815511679,1.6947744392\\

Version=ES64L-G16RevB.01\State=1-A\HF=-757.7245863\RMSD=7.035e-09\RMSF=9.559e-06\Dipole=0.5876398,2.7658935,2.2644358\Quadrupole=1.9136765,-5.4212405,3.507564, 1.7422366, -4.2466177,-6.670406\PG=C01 [X(C14H10N4)]\\@

**7e**

HF= -648.3712086 a.u., NIMAG=0

1\1\GINC-R02N16\FOpt\RPBE1PBE\def2TZVP\C14H10N2\WURTHWE\21-Jun-2021\0\

\# pbe1pbe/def2tzvp opt=(maxstep=5,gdiis) freq pop=nbo emp=gd3bj scrf=

(solvent=dichloromethane)\\Carbene 7e\\0,1\

C,-0.0141899182,0.0494868022,-0.0177333987\C,-0.0462356934,0.0089913093,1.3750841519\

C,1.1519787678,0.0207909937,2.0856032254\C,2.3637806165,0.0767551214,1.4144064745\

C,2.3899333367,0.1266994243,0.0284014776\C,1.1978255215,0.1132343943,-0.6840703045\

C,-1.3573209677,-0.0227425677,2.064822794\N,-1.3634614493,-0.8529152206,3.178419893\

N,-1.387967536,-1.4997696501,4.0714040125\C,-2.5780822326,0.5361792045,1.8856740599\

C,-2.7557380837,1.5127974274,0.836093973\C,-1.9474237076,2.6583504565,0.7435302666\

C,-2.1835713357,3.6151408247,-0.2276290692\C,-3.1974884785,3.4371676497,-1.1613958062\

C,-3.9985419521,2.3044591211,-1.0884346919\C,-3.8055439319,1.3719258939,-0.0830431069\

H,1.2115653061,0.1450619897,-1.7672377408\H,3.33683229,0.1736449203,-0.4963765375\

H,3.2892992321,0.0883150515,1.9779848682\H,1.1433970954,-0.001147251,3.1703402926\

H,-0.9428496093,0.0329651387,-0.5739367635\H,-1.557829284,4.5000181169,-0.2689405348\

H,-1.1376589534,2.794985879,1.4514210694\H,-4.4593797826,0.5107336954,-0.0035299747\

H,-4.7988796401,2.1593686072,-1.8056453477\H,-3.368408997,4.1798725744,-1.9317744978\\

Version=ES64L-G16RevB.01\State=1-A\HF=-648.3712086\RMSD=6.684e-09\RMSF=1.346e-05\Dipole=1.6470446,-0.4863147,-0.0799924\Quadrupole=-1.1886537,-1.7350248,2.9236784, 0.5026405,5.7368112,-3.0910838\PG=C01 [X(C14H10N2)]\\@

**7e-vdw**

HF=-757.8210814 a.u., NIMAG=0

1\1\GINC-R04N05\FOpt\RPBE1PBE\def2TZVP\C14H10N4\WURTHWE\18-May-2025\0\

\# pbe1pbe/def2tzvp opt=(maxstep=6) freq pop=nbo emp=gd3bj scrf=(solvent=dichloromethane)\\ Carbene 7e + N2 vdw\\0,1\

C,-0.19792784,0.058584233,0.1204338047\C,-0.1455854284,0.0904348012,1.5132509611\

C,1.0888650624,-0.0199698526,2.1501298819\C,2.2517194948,-0.154906757,1.4076048607\ C,2.1946101464,-0.1764788594,0.0219025351\C,0.966492587,-0.0692202434,-0.6174894532\

C,-1.4028656445,0.2554131911,2.2781689854\N,-1.4106115552,-0.4583028469,3.4721130111\

N,-1.4436798405,-1.0120236098,4.4250668936\C,-2.5831426668,0.8971351531,2.1149850285\

C,-2.7668102223,1.7651628988,0.97612492\C,-1.9091977444,2.8486864671,0.7233549736\

C,-2.1447656202,3.7045391618,-0.3379961804\C,-3.2095531985,3.4806142398,-1.2027695053\

C,-4.0606142015,2.40770162,-0.9694694005\C,-3.865572603,1.5810668796,0.1243014568\

N,-3.1408439329,-2.307511466,-0.094116914\N,-2.7281623694,-2.5547875965,0.8830750644\ H,0.91299951,-0.0921532011,-1.6996389795\H,3.1037317297,-0.2788805985,-0.5587603823\ H,3.2054435048,-0.2368922602,1.9155236416\H,1.1510218273,0.0105644162,3.2327733194\

H,-1.153592179,0.137056216,-0.3812984306\H,-1.4790230558,4.5444751442,-0.5040804601\

H,-1.0601141798,3.0168036471,1.3763521111\H,-4.5553506122,0.7691832822,0.3254903271\

H,-4.9004973134,2.2279735065,-1.6315098047\H,-3.380469965,4.1426066858,-2.0434629041\\ Version=ES64L-G16RevB.01\State=1-A\HF=-757.8210814\RMSD=3.219e-09\RMSF=1.956e-06\Dipole=1.5807488,-0.6617488,-0.0899824\Quadrupole=0.8890499,-3.220758,2.3317081, 1.6556164,5.3604222,-3.2827877\PG=C01 [X(C14H10N4)]\\@

**TS-1e/8e**

HF=-757.7129358 a.u., NIMAG=1, -481.7555 cm^-1^

1\1\GINC-R10N18\FTS\RPBE1PBE\def2TZVP\C14H10N4\WURTHWE\06-Mar-2025\0\\

# pbe1pbe/def2tzvp opt=(ts,noeigentest,calcfC,maxstep=6) freq pop=nbo emp=gd3bj scrf=(solvent=dichloromethane)\\TS for five-membered ring 8e from bis(diazo)ethane 1e\\0,1\

C,2.8768134663,0.0368185056,-0.4138439619\C,1.6136827624,-0.0430564623,0.1852462829\

C,1.2403249788,-1.2248835186,0.8339602091\C,2.0984326693,-2.3093648453,0.8528909865\

C,3.3400195032,-2.2288164073,0.2364483336\C,3.7278584737,-1.0508784096,-0.3924595419\

C,0.7155239968,1.0855226113,0.1438346795\N,1.1348047596,2.3295475958,-0.0963312072\

N,0.7501739775,3.3605651921,-0.824005847\C,-0.7339238396,1.1583285537,0.137082852\

N,-1.1336203441,2.3940489052,0.1036777956\N,-0.0822413928,3.2407213,0.3489297489\

C,-1.667974233,0.0381955484,-0.038895534\C,-1.4086234916,-0.9852635594,-0.9482758468\

C,-2.3201556689,-2.014568104,-1.1117405319\C,-3.4919482645,-2.0335892118,-0.3670046069\

C,-3.7559133504,-1.0134238037,0.5361472388\C,-2.8506152065,0.0227646831,0.6975945464\

H,-2.1162508783,-2.8033400653,-1.8260046909\H,-4.2011931008,-2.8431576414,-0.4933717001\

H,-4.6702601169,-1.0236074239,1.11740781\H,-3.0497704889,0.8234830898,1.3994421015\

H,-0.4980172575,-0.9716970226,-1.5346816991\H,1.8004467175,-3.2197709575,1.358837077\

H,0.2788765588,-1.2879299553,1.3269315786\H,3.1847570912,0.9597034037,-0.8916498758\

H,4.7008652219,-0.9813796472,-0.8637327492\H,4.0114804569,-3.0790113536,0.2559345523

\\Version=ES64L-G16RevB.01\State=1-A\HF=-757.7129358\RMSD=9.147e-09\RMSF=3.944e-06\Dipole=0.3919434,-2.9975928,0.1498559\Quadrupole=10.9415845,-9.1662147,-1.7753698, 0.7591891,-3.6931581,1.4610196\PG=C01 [X(C14H10N4)]\\@

**8e**

HF=-757.8059211 a.u., NIMAG=0

1\1\GINC-R03N20\FOpt\RPBE1PBE\def2TZVP\C14H10N4\WURTHWE\23-Jan-2025\0\

\# pbe1pbe/def2tzvp opt=(maxstep=5) freq pop=nbo emp=gd3bj scrf=(solve

nt=dichloromethane)\\Five-membered ring 8e\\0,1\

N,-0.5571883548,-2.2293386983,-0.1456261465\C,1.0333386729,-0.5454562095,-0.0490744943\

N,1.764742815,-1.8347631012,-0.068818504\N,0.3912269588,-2.946939158,-0.1446034504\

C,-0.2715480906,-0.7769855545,-0.0429941941\N,2.882612739,-2.1155801493,-0.0592710054\

C,2.2545503949,2.839709585,-1.0396122032\C,1.5051391799,1.6781890627,-0.9951157927

\C,1.8234579982,0.6718259083,-0.0804272374\C,2.9102543343,0.8422117602,0.7771124961\

C,3.652389136,2.0121867208,0.7317910117\C,3.328119061,3.0112209595,-0.1739675954\

C,-1.4298555939,0.0883755677,0.0664853768\C,-1.3460031076,1.3317867913,0.6999926849\

C,-2.464116162,2.1382194357,0.8010114422\C,-3.679046921,1.721006059,0.2696417104\

C,-3.7719943125,0.4852525779,-0.3538324241\C,-2.6577638869,-0.3319854374,-0.4497431249\

H,2.0029475104,3.6133215276,-1.7553740712\H,0.6701128306,1.5397750525,-1.67146569\

H,3.1632624431,0.0680545172,1.4899425224\H,4.487919898,2.1423944706,1.4092836545\

H,3.9123565688,3.9232432752,-0.2085365337\H,-0.404292287,1.658007614,1.1226462431\

H,-2.3901769832,3.0966890338,1.3009523748\H,-4.5525635252,2.3575857312,0.3477008211\

H,-4.7176006427,0.153546738,-0.7659919525\H,-2.7304786734,-1.29954008,-0.929790918\\ Version=ES64L-G16RevB.01\State=1-A\HF=-757.8059211\RMSD=9.036e-09\RMSF=1.051e-05\Dipole=-0.9549445,2.4167855,0.0930005\Quadrupole=4.9233772,-6.4376061, 1.5142289,5.7792369,3.9182247,0.0840367\PG=C01 [X(C14H10N4)]\\@

Table S6 Species f R=CN**:** Total energies (E_tot_) and Gibbs free energies (G_298_) [a.u.] of compounds **f** and of the related transition states as given in the Gaussian archive entries. The relative energies [kcal/mol] include all involved species according to the respective reactions as given in the schemes in the manuscript. Van der Waals energies are given in italics.

| **Species f**  **R=CN** | **E_tot_ [a.u.]** | **E_rel_ [kcal/mol]** | **G_298_ [a.u.]** | **E_rel_ [kcal/mol]** |
| --- | --- | --- | --- | --- |
| **N_2_** | -109.44698 |  | -109.45973 |  |
| **1f** | -480.41996 | -6.78 | -480.40921 | 0.00 |
| **TS-1f/2f** | -480.38866 | 12.86 | -480.37627 | 20.67 |
| **2f** | -480.40916 | 0.00 | -480.39515 | 8.83 |
| **TS-1f/3f** | -480.37552 | 21.11 | -480.36802 | 25.84 |
| **3f** | -261.60314 |  | -261.59965 |  |
| **3f+2N_2_** | -480.49709 | -55.18 | -480.51911 | -68.97 |
| **3f-vdw** | *-480.49967* | *-56.80* | *-480.51081* | *-63.75* |
| **TS-2f/4f** | -480.34599 | 39.64 | -480.33948 | 43.75 |
| **4f** | -185.50857 |  | -185.51268 |  |
| **4f+2N_2_** | -480.46412 | -34.49 | -480.48509 | -47.62 |
| **4f-vdw** | *-480.46823* | *-37.07* | *-480.47524* | *-41.43* |
| **TS-1f/7f** | -480.38714 | 13.82 | -480.38009 | 18.27 |
| **TS-3f/7f** | -370.96430 |  | -370.96318 |  |
| **TS-3f/7f +N_2_** | -480.41128 | -1.33 | -480.42292 | -8.60 |
| **TS-3f/7f-vdw** | *-480.41330* | *-2.60* | *-480.41678* | *-4.75* |
| **7f** | -370.96854 |  | -370.96523 |  |
| **7f+N_2_** | -480.41552 | -3.99 | -480.42496 | -9.88 |
| **7f-vdw** | *-480.41750* | *-5.23* | *-480.41858* | *-5.88* |
| **TS-3f/8f** | -480.27772 | 82.48 | -480.26730 | 89.05 |

**Gaussian Archive Entries**

(Total energies (a.u.), number of imaginary frequencies (for transition states: imaginary frequencies), coordinates)

**1f**

HF=-480.4199585° a.u., NIMAG=0

1\1\GINC-R09N18\FOpt\RPBE1PBE\def2TZVP\C4N6\WURTHWE\11-Nov-2024\0\\# p

be1pbe/def2tzvp opt=(maxstep=5) freq pop=nbo emp=gd3bj scrf=(solvent=dichloromethane)\\ Dicyano-bis-1,2-(diazo)ethane\\0,1\

C,-0.1090516579,-0.2427371877,-0.0712710007\N,-0.1662157764,-0.4433678792,1.0646973545\

C,0.0087189746,-0.0323537761,-1.4574887126\C,1.1387958003,-0.4586789099,-2.2674665764\

C,2.2836150306,0.3195065339,-2.5195766506\N,3.2013336168,0.9955300817,-2.7059560201\

N,-0.9684173094,0.6327323105,-2.0534068083\N,-1.7722812637,1.1936821645,-2.5818336854\

N,1.1117147253,-1.6786180994,-2.78060322\N,1.0619408943,-2.7110822212,-3.1948920496\\ Version=ES64L-G16RevB.01\State=1-A\HF=-480.4199585\RMSD=3.563e-09\RMSF=3.953e-05\Dipol

e=-1.3508521,-0.7465175,-1.4913127\Quadrupole=-0.5138586,4.932769,-4.4189104,-5.7291722, 3.8001012,2.9785628\PG=C01 [X(C4N6)]\\@

**TS-1f/2f**

HF=-480.3886611 a.u., NIMAG=1, -326.3648cm^-1^

1\1\GINC-R02N09\FTS\RPBE1PBE\def2TZVP\C4N6\WURTHWE\24-Oct-2024\0\\# pb

e1pbe/def2tzvp opt=(ts,noeigentest,calcfC,maxstep=12,maxcycles=200) freq pop=nbo emp=gd3bj scrf=(solvent=dichloromethane)\\TS for tetrazine formation 2f\\0,1\

N,0.6482495584,0.5466785517,0.3598631447\N,0.6506362255,-0.5425266709,1.8507101982\

N,1.7475381673,-0.5215806893,2.2532721873\C,2.9508666018,-0.0474918222,1.790365253\

C,2.948023509,0.047621827,0.4107568909\N,1.7434537767,0.5239675532,-0.0472634202\

C,3.9819352074,0.2315345767,2.7056480585\N,4.8499350215,0.4664557372,3.4260575729\

C,3.9752079737,-0.2326829651,-0.5085403262\N,4.840031763,-0.4685844262,-1.2324396348\\

Version=ES64L-G16RevB.01\State=1-A\HF=-480.3886611\RMSD=3.185e-09\RMSF=4.874e-05\Dipole=-1.5694441,0.0008984,0.0034686\Quadrupole=-4.7440051,6.8036618,-2.0596567, 0.0046613,0.0051495,-1.7643747\PG=C01[X(C4N6)]\\@

**2f**

HF=-480.4091601 a.u., NIMAG=0

1\1\GINC-R03N40\FOpt\RPBE1PBE\def2TZVP\C4N6\WURTHWE\23-Oct-2024\0\\# pbe1pbe/ def2tzvp opt=(maxstep=5) freq pop=nbo emp=gd3bj scrf=(solvent=dichloromethane)\\ Dicyanotetrazine 2f\\0,1\

N,0.0548937471,0.6694804612,-1.784281645\N,0.0576194384,1.3571776831,-0.6735584095\

C,0.0090852767,0.7057000441,0.480139245\C,-0.0399281443,-0.6866547928,0.4758228467\

N,-0.0388727485,-1.3329249344,-0.6818640913\N,0.006631232,-0.6400011151,-1.7884297918\

C,0.0104532334,1.4758342191,1.6833778551\C,-0.0902712868,-1.4640237881,1.6724661599\

N,0.0102755801,2.070490933,2.6665238401\N,-0.1297273281,-2.0684697102,2.6487639908\\

Version=ES64L-G16RevB.01\State=1-A\HF=-480.4091601\RMSD=3.360e-09\RMSF=1.545e-04\Dipole=0.0079832,0.0045877,-0.4374923\Quadrupole=9.4570245,-2.057507,-7.3995175,-0.

4021148,0.3424427,0.011037\PG=C01 [X(C4N6)]\\@

**TS-1f/3f**

HF=-480.3755181 a.u., NIMAG=2, -558.0446 cm^-1^, -189,0960 cm^-1^

1\1\GINC-R09N35\FTS\RPBE1PBE\def2TZVP\C4N6\WURTHWE\11-Nov-2024\0\\# pb

e1pbe/def2tzvp opt=(ts,noeigentest,calcfC,maxstep=6) freq pop=nbo emp=

gd3bj scrf=(solvent=dichloromethane)\\TS for alkyne formation from dicyanotetrazine\\0,1\

C,-0.2109911653,-0.2169126055,0.0685860565\N,-0.4711116802,-0.3783293985,1.1813115671\

C,0.1102633382,-0.0521380546,-1.2737962611\C,1.1865330918,-0.114519679,-1.9990034657\

C,2.5574955322,-0.0746281208,-1.7728525121\N,3.6934012265,-0.0173840602,-1.5770843712\

N,-1.111123333,0.7027788408,-2.0603109934\N,-1.4157510898,0.7329097802,-3.1423718481\

N,0.8763879418,-0.6877790911,-3.5010646486\N,-0.0048612615,-0.5770520106,-4.1907669312\\

Version=ES64L-G16RevB.01\State=1-A\HF=-480.3755181\RMSD=4.213e-09\RMSF=7.011e-05\Dipole=-1.0415631,0.1239348,-1.5555918\Quadrupole=-4.0599269,7.52201,-3.4620831,-0.574063,0.5228464,1.2375038\PG=C01 [X(C4N6)]\\@

**3f**

HF=-261.6031392 a.u., NIMAG=0

1\1\GINC-R01N11\FOpt\RPBE1PBE\def2TZVP\C4N2\WURTHWE\23-Oct-2024\0\\# p

be1pbe/def2tzvp opt=(maxstep=5) freq pop=nbo emp=gd3bj scrf=(solvent=dichloro methane)\\Dicyanoethyne\\0,1\

N,-1.963302649,2.140841457,0.\C,-1.4048791084,1.1303131078,0.\

C,-0.7450147825,-0.0625724649,0.\C,-0.1614944104,-1.1179339926,0.\

C,0.4965702567,-2.3117267201,0.\N,1.0524256937,-3.3236193872,0.\\

Version=ES64L-G16RevB.01\State=1-A'\HF=-261.6031392\RMSD=6.697e-09\RMSF=7.464e-05\Dipole=0.001917,0.0010412,0.\Quadrupole=1.6435563,-7.1177949,5.4742387,6.9452202, 0.,0.\PG=CS [SG(C4N2)]\\@

**3f-vdw**

HF=-480.4996749 a.u., NIMAG=0

1\1\GINC-R01N26\FOpt\RPBE1PBE\def2TZVP\C4N6\WURTHWE\10-May-2025\0\\# p

be1pbe/def2tzvp opt=(maxstep=6) geom=check guess=read freq pop=nbo emp=gd3bj scrf=(solvent=dichloromethane)\\Dicyanoethyne + 2 N2 vdw\\0,1\

C,-0.090997551,-1.5627059922,-0.5398638237\N,-0.1401329612,-2.7134886756,-0.615840795\

C,-0.0371023551,-0.2042788674,-0.4461660583\C,0.0036207517,0.9974110997,-0.3555095288\

C,0.0496490954,2.3556517008,-0.2530426931\N,0.0887563515,3.5062492965,-0.1651997974\

N,3.25453632,-0.8298026078,0.1890279495\N,4.0431442172,-0.3757752777,0.7876015768\

N,-3.1811408225,-0.5855432913,0.925894055\N,-4.0013822796,-0.3611709263,0.2452591775\\

Version=ES64L-G16RevB.01\State=1-A\HF=-480.4996749\RMSD=9.328e-09\RMSF=1.272e-04\Dipole=0.016346,-0.0096564,-0.0018384\Quadrupole=5.1813951,-10.4213668,5.2399717,-0.8026475,-1.0779939,-1.3014491\PG=C01 [X(C4N6)]\\@

**TS-2f/4f**

HF =-480.3459913 a.u., NIMAG=1, -345.5741 cm^-1^

1\1\GINC-R04N05\FTS\RPBE1PBE\def2TZVP\C4N6\WURTHWE\03-May-2025\0\\# pb

e1pbe/def2tzvp opt=(ts,noeigentest,readfC,maxstep=1) geom=check guess= read freq pop=nbo emp=gd3bj scrf=(solvent=dichloromethane)\\TS for formation of 2 dicyanes + N2\\0,1\

N,2.6304215768,-0.5582737757,-0.0381945234\N,0.9421081915,-1.586341117,-0.0547680204\

C,-0.0158424707,-0.833687891,-0.0127752715\C,-0.0157643158,0.8336951642,0.0127770531\

N,0.9422591898,1.5862586703,0.0547187423\N,2.6304765989,0.5580313582,0.0380550006\

C,-1.338625094,-1.3655831216,0.0207694887\C,-1.338499517,1.3657129709,-0.0206971815\

N,-2.3991227189,-1.808080974,0.0502334332\N,-2.3989574405,1.8083097156,-0.050104721

\\Version=ES64L-G16RevB.01\State=1-A\HF=-480.3459913\RMSD=9.375e-09\RMSF=7.098e-06\Dipole=1.3228166,-0.0000637,-0.0000353\Quadrupole=-5.6731254,-1.8561789,7.5293043, 0.0001708,0.0003497,-0.0355081\PG=C01 [X(C4N6)]\\@

**NC-CN**

**4f**

HF=-185.5085732 a.u., NIMAG=0

1\1\GINC-R03N34\FOpt\RPBE1PBE\def2TZVP\C2N2\WURTHWE\11-Dec-2024\0\\# p

be1pbe/def2tzvp opt=(maxstep=5) freq pop=nbo emp=gd3bj scrf=(solvent=d

ichloromethane)\\Cyanogen\\0,1\

N,-1.5620606584,0.4309329508,0.\C,-0.419446703,0.2977387117,0.\

C,0.9481007477,0.1320682621,0.\N,2.0894666217,-0.0114284209,0.\\

Version=ES64L-G16RevB.01\State=1-A1\HF=-185.5085732\RM

SD=2.051e-09\RMSF=2.863e-04\Dipole=0.0007545,0.006228,0.\Quadrupole=-4

.9482012,2.41924,2.5289612,0.9058165,0.,0.\PG=C02V [SGV(C2N2)]\\@

**NC-CN + 2 N_2_**

**4f-vdw**

HF= -480.4682322a.u., NIMAG=0

1\1\GINC-R01N17\FOpt\RPBE1PBE\def2TZVP\C4N6\WURTHWE\04-Jun-2025\0\\# pbe1pbe/def2tzvp opt=(maxstep=15,maxcycle=120) geom=check guess=read freq pop=nbo emp=gd3bj scrf=(solvent=dichloromethane)\\cyanogen + N2 vdw\\ 0,1\N,2.0905747614,-0.1452247178,0.1259845061\N,-1.0706662129,-1.0214195198,2.1761953642\ C,-1.2019001122,-1.590419379,1.1850168432\C,-0.5015461414,1.7592143935,-0.7965914622\

N,-1.2886134278,0.9252228817,-0.8858831276\N,2.6072244075,-1.0439581841,0.459835718\

C,-1.3497168329,-2.2765118095,0.0002617004\C,0.4430841773,2.7548055825,-0.685857336\

N,-1.4671555682,-2.8558875597,-0.9865671053\N,1.2325719491,3.5863323121,-0.5930901007\\ Version=ES64L-G16RevB.01\State=1-A\HF=-480.4682322\RMSD=4.417e-09\RMSF=5.528e-06\ Dipole=0.0276825,0.0397953,-0.0160491\Quadrupole=1.7035304,-1.2566623,-0.4468681,-3.7323352,-1.2513732,-3.4058452\PG=C01 [X(C4N6)]\\@

**TS-1f/7f**

HF=-480.3871367 a.u., NIMAG=1, -530.7243 cm^-1^

1\1\GINC-R01N14\FTS\RPBE1PBE\def2TZVP\C4N6\WURTHWE\23-Feb-2025\0\\# pb

e1pbe/def2tzvp opt=(ts,noeigentest,calcfc,maxstep=6,tight) geom=check guess=read freq pop=nbo emp=gd3bj scrf=(solvent=dichloromethane)\\TS for formation of dicyanocarbene\\0,1\

C,-0.1850766874,-0.286135697,-0.174124836\N,- 0.2672428794,-0.7748155705,0.8671803957\ C,0.0041375247,0.2886280523,-1.4550852805\C,1.1215094898,0.2294890074,-2.2938043885\ C,2.4005273563,0.0355427531,-1.7577264998\N,3.4845299267,-0.0222800217,-1.3552702539\

N,-1.0535032091,0.9737262391,-1.9424798111\N,-1.9092680484,1.5295934375,-2.3673252498\ N,0.961980543,-1.1286197619,-3.4070772215\N,0.6526485845,-1.5281828375,-4.3816402625\\ Version=ES64L-G16RevB.01\State=1-A\HF=-480.3871367\RMSD=6.521e-09\RMSF=1.415e-05\Dipole=-2.1792849,0.4858334,-1.9133941\Quadrupole=-3.2988441,6.3544683,-3.0556242,-2.8541662,-0.8424194,4.0408183\PG=C01 [X(C4N6)]\\@

**TS-3f/7f**

HF=-370.9643042 a.u., NIMAG=1, -464.8377 cm^-1^

1\1\GINC-R02N06\FTS\RPBE1PBE\def2TZVP\C4N4\WURTHWE\09-May-2025\0\\# pb

e1pbe/def2tzvp opt=(ts,noeigentest,calcfc,maxstep=6) freq pop=nbo emp=gd3bj scrf=(solvent=dichloromethane)\\TS for elimination of N2 from carbene 7f \\0,1\ C,0.6751519381,1.3664586654,0.0001556609\N,0.9267939133,2.490641616,0.0002025743\ C,0.2873612055,0.0131835531,0.00009535\C,-0.7759384652,-0.712174133,0.0000510953\

C,-2.1037662592,-0.3758353695,0.00002145\N,-3.2494710918,-0.1839740387,-0.0000093295\ N,1.6113807947,-0.8735232534,0.0000739633\N,2.3572099645,-1.67157904,0.0000202356\\ Version=ES64L-G16RevB.01\State=1-A\HF=-370.9643042\RMSD=5.060e-09\RMSF=5.265e-06\

Dipole=2.7388728,-1.3440554,0.0000062\Quadrupole=-4.4257991,-1.5905315,6.0163306, -4.9293357,-0.0004952,-0.000394\PG=C01 [X(C4N4)]\\@

**TS-3f/7f-vdw**

HF=-480.4132966 a.u., NIMAG=1, -464.6315 cm^-1^

1\1\GINC-R01N26\FTS\RPBE1PBE\def2TZVP\C4N6\WURTHWE\10-May-2025\0\\# pb

e1pbe/def2tzvp opt=(ts,noeigentest,calcfc,maxstep=6) freq pop=nbo emp=gd3bj scrf=(solvent=dichloromethane)\\TS for N2-elimination from carbene vdw\\0,1\

C,-0.6399388533,-1.0277862117,0.961653826\N,-0.3196378392,-1.9336633437,1.5971716898\

C,-0.9572366131,0.1161413137,0.2053381716\C,-0.4643658939,1.2630305672,-0.1078755615\ C,0.7164572985,1.8673861215,0.2328401809\N,1.6923305316,2.4523912038,0.4671613271\

N,-2.382355172,-0.1214234849,-0.4667672677\N,-3.3182665477,-0.0558279536,-1.0261858018\

N,2.2085676545,-1.0195257219,-0.9368622228\N,3.2649554345,-1.2063864904,-0.7491563415\\ Version=ES64L-G16RevB.01\State=1-A\HF=-480.4132966\RMSD=8.113e-09\RMSF=9.168e-06\Dipole=-2.7131811,-0.815948,-1.0927178\Quadrupole=6.7222127,-8.0451084,1.3228956,-4.1310079,-0.2822159,2.8246585\PG=C01 [X(C4N6)]\\@

**7f**

HF=-370.9685416 a.u., NIMAG=0

1\1\GINC-R03N29\FOpt\RPBE1PBE\def2TZVP\C4N4\WURTHWE\09-May-2025\0\\# p

be1pbe/def2tzvp opt=(maxstep=6) freq pop=nbo emp=gd3bj scrf=(solvent=dichloromethane)\\ dicyano-carbene 7f\\0,1\

C,-1.1741579526,-1.2981537715,-0.393621158\N,-1.4975241332,-2.1887816559,-1.047495133\

C,-0.6778844908,-0.241343721,0.4235902116\C,0.5129063379,-0.0177160267,1.0022490412\

C,1.5391503456,-0.9335430789,0.8379957515\N,2.4717587799,-1.6237880373,0.7742662045\

N,-1.6315480724,0.760160254,0.6659462583\N,-2.3457138143,1.5638230372,0.877299824\\ Version=ES64L-G16RevB.01\State=1-A\HF=-370.9685416\RMSD=6.658e-09\RMSF=7.665e-05\Dipole=-2.408352,2.1186403,0.3663946\Quadrupole=-3.1541237,0.9671009,2.1870228,-1.1676582,-3.9656276,-3.1944139\PG=C01 [X(C4N4)]\\@

**7f-vdw**

HF=-480.4175021 a.u., NIMAG=0

1\1\GINC-R01N02\FOpt\RPBE1PBE\def2TZVP\C4N6\WURTHWE\18-May-2025\0\\# p

be1pbe/def2tzvp opt=(maxstep=6) freq pop=nbo emp=gd3bj scrf=(solvent=dichloromethane)\\Dicyanocarbene 7f vdW\\0,1\

C,-1.1266522805,0.7127858644,-0.5510891022\N,-1.2754171062,1.7674255277,-0.9880061501\

C,-0.8471853564,-0.5951382568,-0.0597028256\C,0.2016412635,-1.420026339,-0.2081653431\ C,1.286776565,-1.0224223561,-0.9711399273\N,2.2507388794,-0.802361014,-1.581657143\N,-1.8781826193,-1.1146482132,0.7388421916\N,-2.6602004219,-1.5532177201,1.3687251906\ N,1.6494571649,1.6245116803,1.1908352814\N,2.3997439114,2.4080838267,1.0952398275\\

Version=ES64L-G16Rev B.01\State=1-A\HF=-480.4175021\RMSD=7.251e-09\RMSF=2.674e-05\ Dipole=-2.5216232,-0.8876281,1.7757287\Quadrupole=1.9447618,-2.4854679,0.5407061, 7.2311701,1.3275082,0.5022019\PG=C01 [X(C4N6)]\\@

**TS-1f/8f**

HF=-480.2777228 a.u., NIMAG=1, -567.3270 cm^-1^

1\1\GINC-R08N36\FTS\RPBE1PBE\def2TZVP\C4N6\WURTHWE\06-Mar-2025\0\\# pb

e1pbe/def2tzvp opt=(ts,noeigentest,calcfc,maxstep=6) freq pop=nbo emp= gd3bj scrf=(solvent=dichloromethane)\\TS for five-membered ring 8f from 1f\\0,1\

N,1.9159908731,-0.6776116659,-0.4568953689\N,0.8607031462,-1.465847484,-0.1493304041\

C,-0.2308854641,-0.7626636497,-0.1050644993\C,0.0167063894,0.6604876444,-0.1536721134\ N,1.3392432845,0.735335962,-0.0119036752\N,2.2966022559,0.3002067609,0.6651839204\

C,-1.5194353169,-1.3360044061,0.0775137143\C,-0.8643294802,1.7432826581,-0.0818194866\

N,-2.573453996,-1.772230506,0.2143014688\N,-1.613109692,2.6186436864,-0.0348815562\\

Version=ES64L-G16RevB.01\State=1-A\HF=-480.2777228\RMSD=8.928e-09\RMSF=2.530 e-05\ Dipole=1.284502,0.1912349,-0.0554262\Quadrupole=-3.8976508,-2.516915,6.4145658, 2.4440274,0.8227997,-0.0344153\PG=C01 [X(C4N6)]\\@

Table S7 Species g R=NMe_2_**:** Total energies (E_tot_) and Gibbs free energies (G_298_) [a.u.] of compounds **g** and of the related transition states as given in the Gaussian archive entries. The relative energies [kcal/mol] include all involved species according to the respective reactions as given in the schemes in the manuscript. Van der Waals energies are given in italics.

| **Species g**  **R=NMe_2_** | **E_tot_ [a.u.]** | **E_rel_ [kcal/mol]** | **G_298_ [a.u.]** | **E_rel_ [kcal/mol]** |
| --- | --- | --- | --- | --- |
| **N_2_** | -109.44698 |  | -109.45973 |  |
| **1g** | -563.79092 | 42.69 | -563.63958 | 0.00 |
| **TS-1g/2g** | -563.77406 | 53.28 | -563.62064 | 11.88 |
| **2g** | -563.85896 | 0.00 | -563.69789 | -36.59 |
| **3g** | -344.99043 |  | -344.85189 |  |
| **3g+2N_2_** | -563.88438 | -15.95 | -563.77136 | -82.69 |
| **3g-vdw** | *-563.88983* | *-19.37* | *-563.75769* | *-74.12* |
| **4g** | -227.22700 |  | -227.16496 |  |
| **4g+N_2_** | -563.90098 | -26.36 | -563.78965 | -94.17 |
| **TS-2g/7g** | -563.78221 | 48.16 | -563.63038 | 5.77 |
| **TS-3g/7g** | -454.36595 |  | -454.22331 |  |
| **TS-3g/7g+N_2_** | -563.81293 | 28.89 | -563.68305 | -27.28 |
| **TS-3g/7g-vdw** | *-563.81558* | *27.22* | *-563.67550* | *-22.54* |
| **7g** | -454.38032 |  | -454.23424 |  |
| **7g+N_2_** | -563.82730 | 19.87 | -563.69398 | -34.14 |
| **7g-vdw** | -563.82992 | 18.22 | -563.68709 | -29.82 |
| **TS-2g/8g** | -563.71625 | 89.55 | -563.56056 | 49.59 |
| **8g** | -563.78867 | 44.11 | -563.63446 | 3.21 |
| **TS-2g/9g** | -563.73384 | 78.51 | -563.58204 | 36.11 |
| **9g** | -563.83168 | 17.12 | -563.67122 | -19.86 |

**Gaussian Archive Entries**

(Total energies (a.u.), number of imaginary frequencies (for transition states: imaginary frequencies), coordinates)

**1g**

HF=-563.7909233 a.u., NIMAG=0

1\1\GINC-R08N43\FOpt\RPBE1PBE\def2TZVP\C6H12N6\WURTHWE\03-Feb-2025\0\\

# pbe1pbe/def2tzvp opt=(maxstep=5) freq pop=nbo emp=gd3bj scrf=(solvent=dichloro methane)\\ Bisdimethylaminodbisdiazomethane, best rotamer\\0,1\

N,-1.8200777838,-0.0310679343,-2.2301643517\N,-1.2499642646,-0.110226701,-1.2448831647\

C,-0.7202049234,-0.1576157829,-0.0625564725\C,0.7211755311,-0.1581858698,0.0633387365\

N,1.2509653079,-0.1140850896,1.2457814602\N,1.8209944888,-0.037845741,2.231339007\

N,-1.5600202708,-0.2789039043,1.056309658\N,1.560971353,-0.2769822295,-1.0558122442\

C,2.0335988804,-1.6268449607,-1.3125742064\C,-2.0327626256,-1.6293168802,1.3099753503\

C,2.6023280971,0.7257689818,-1.1472725397\C,-2.6012671268,0.7237347505,1.1501141319\

H,2.7867257623,-1.9571386104,-0.581653336\H,1.1909490349,-2.3185666568,-1.2823251476\

H,2.4808047686,-1.6690856157,-2.3080435274\H,-1.1901799122,-2.321046425,1.2780652423\

H,-2.4799006567,-1.6738256755,2.305376686\H,-2.7859642468,-1.9578397564,0.5783356043\

H,3.3902152018,0.5980255093,-0.3881535325\H,3.0722689188,0.6635734878,-2.1307182607\

H,2.1655444334,1.718149856,-1.0313721627\H,-3.0712115209,0.6592868539,2.1334120871\

H,-2.1643816249,1.7163399709,1.0365363368\H,-3.3891728218,0.5978574229,0.3907026458\\ Version=ES64L-G16RevB.01\State=1-A\HF=-563.7909233\RMSD=6.043e-09\RMSF=1.856e-06\Dipole=-0.0000105,-0.3522927,-0.0003328\Quadrupole=3.9373892,3.1186744,-7.0560635, 0.0138971,-10.8141404,0.0151271\PG=C01 [X(C6H12N6)]\\@

**TS-1g/2g**

HF=-563.7740562 a.u., NIMAG=1, -195.1690 cm^-1^

1\1\GINC-R04N05\FTS\RPBE1PBE\def2TZVP\C6H12N6\WURTHWE\03-May-2025\0\\#

pbe1pbe/def2tzvp opt=(ts,noeigentest,readfC,maxstep=1) geom=check guess=read freq pop=nbo emp=gd3bj scrf=(solvent=dichloromethane)\\TS for 1,2,3,4-tetrazine ring closure to give bisdimethylaminotetrazine\\0,1\

C,0.0357821624,-0.0233121316,-0.0104206174\N,0.0212928505,-0.0018761721,1.4358198043\

C,1.3443810249,-0.0088491768,2.0318825118\C,-0.8600344469,-0.9182824505,2.0274193579\

N,-1.7657437205,-1.5614060839,1.3244949531\N,-2.4909110296,-2.4193158009,1.0605871171\

C,-0.9501184245,-1.2846255261,3.397852059\N,-0.8228852742,-0.4510888578,4.520734013\

C,-1.4905822173,0.8283442108,4.361126074\N,-1.1933858604,-2.5701046956,3.5061683335\

N,-1.5691687038,-3.6137190993,3.192005939\C,-1.1009321658,-1.0960349097,5.7848783646\

H,0.4497476876,-0.9563197979,-0.4214956712\H,-0.9783716317,0.1048340599,-0.394132533\

H,0.6427818197,0.8097293107,-0.3648077138\H,-0.4824722893,-1.9896083238,5.8895049137\

H,-0.8473670276,-0.4069511596,6.5904806709\H,-2.1565452753,-1.3869110524,5.8966299802\

H,1.9071577874,-0.92407948,1.7957391542\H,1.9005126292,0.8494250631,1.6533527994\

H,1.2628460152,0.0794256599,3.1156793014\H,-1.1879715973,1.483962437,5.1784445727\

H,-1.1885525002,1.2877202328,3.4191804864\H,-2.5866968243,0.7357119893,4.3708420238\\ Version=ES64L-G16RevB.01\State=1-A\HF=-563.7740562\RMSD=9.010e-09\RMSF=1.918 e-06\ Dipole=1.0924904,1.8295226,0.5810888\Quadrupole=-0.7575062,-6.5626065,7.3201127,-3.3255209,-5.1748084,-2.0252519\PG=C01 [X(C6H12N6)]\\@

**2g**

HF=-563.8589589 a.u., NIMAG=0

1\1\GINC-R03N37\FOpt\RPBE1PBE\def2TZVP\C6H12N6\WURTHWE\23-Oct-2024\0\\

# pbe1pbe/def2tzvp opt=(maxstep=5) freq pop=nbo emp=gd3bj scrf=(solven

t=dichloromethane)\\ Bisdimethylamino-tetrazine 2g\\0,1\

N,-0.0225346323,0.7806764418,-1.8980427001\N,-0.4482060746,1.3562422908,-0.7820531467\

C,-0.2486361011,0.7769521228,0.3892533141\C,0.1812169006,-0.5934447829,0.3677814178\

N,0.7397667828,-1.0243942401,-0.7500317072\N,0.6635527981,-0.3044080945,-1.8608043673\

N,-0.4978285522,1.4954592555,1.5111334224\N,0.0905837167,-1.4523636016,1.4125168295\

C,0.6965694631,-2.7599778534,1.270123716\C,-1.058447915,2.8220582187,1.3568294543\

C,-1.0647027402,-1.4259652063,2.2913790637\C,0.3326814687,1.3349865714,2.690577082\

H,0.1138644509,-3.4115319138,0.6091156033\H,1.7000378099,-2.6671873981,0.8607433056\

H,0.7468256505,-3.2149437748,2.2594273248\H,-1.8870075503,2.8008388689,0.6524444457\

H,-1.4174327068,3.1489524578,2.3328031345\H,-0.3154102707,3.540797249,0.9931401941\

H,-1.8153896939,-2.1453734066,1.9457906457\H,-0.7632354715,-1.6978036409,3.3042002027\

H,-1.5202242372,-0.439819967,2.3073111473\H,-0.2698342241,1.4816616325,3.5884114085\

H,0.7814613475,0.3460457562,2.7195851983\H,1.1384997811,2.0774010145,2.6860980112

\\Version=ES64L-G16RevB.01\State=1-A\HF=-563.8589589\RMSD=5.451e-09\RMSF=4.500e-05\Dipole=-0.6197437,-0.2548252,3.9420394\Quadrupole=1.8715489,6.9101261,-8.781675, 1.3018114,1.8111887,1.2476401\PG=C01 [X(C6H12N6)]\\@

**3g**

HF=-344.9904271 a.u., NIMAG=0

1\1\GINC-R01N39\FOpt\RPBE1PBE\def2TZVP\C6H12N2\WURTHWE\23-Oct-2024\0\\

# pbe1pbe/def2tzvp opt=(maxstep=5) freq pop=nbo emp=gd3bj scrf=(solvent=dichloromethane)\ \Bisdimethylaminoethine\\0,1\

C,-2.6974558421,-0.7591164992,-0.8875912572\N,-1.9321598429,-0.1790957821,0.2006556117\

C,-0.6035182241,-0.1319367146,0.0610078784\C,0.6024793698,-0.1309331842,-0.0724449175\

N,1.9310049332,-0.1763368325,-0.2137840434\C,2.6946019222,-0.7996149571,0.8515097191\

C,-2.5751944188,0.9406983241,0.862405204\C,2.5771917429,0.9661868988,-0.8321889147\

H,-2.2038213086,-1.666076504,-1.2342554451\H,-2.7999047656,-0.0676875483,-1.7358943964\

H,-3.6970897905,-1.0167911979,-0.5304525258\H,2.1985231813,-1.7178716814,1.1631765489\

H,2.7987510155,-0.1414215241,1.7256489477\H,3.6936061937,-1.0460607563,0.4848102559\

H,-3.5758939339,0.6448938669,1.1851779641\H,-1.9937399718,1.2260358114,1.7381264701\

H,-2.6683061121,1.8141956853,0.2016644152\H,1.9965317925,1.2865580764,-1.6962436532\

H,3.577041893,0.6801439743,-1.1662023073\H,2.6728081664,1.8134265445,-0.1384385544\\ Version=ES64L-G16RevB.01\State=1-A\HF=-344.9904271\RMSD=6.158e-09\RMSF=2.690e-06\Di

pole=0.0004063,0.3109131,0.0060398\Quadrupole=6.140073,-3.0380514,-3.1020216,-0.0258673,0.7121034,0.0002475\PG=C01 [X(C6H12N2)]\\@

**3g-vdw**

HF=-563.8898255 a.u., NIMAG=0

1\1\GINC-R01N25\FOpt\RPBE1PBE\def2TZVP\C6H12N6\WURTHWE\17-May-2025\0\\

# pbe1pbe/def2tzvp opt=(maxstep=6) freq pop=nbo emp=gd3bj scrf=(solvent=dichloromethane)\\ Bisdimethylaminoethine + 2N2 vdw\\0,1\

C,-2.8852831384,-1.5066216124,-0.0804661121\N,-1.9605329468,-0.6827170594,-0.8353445061\ C,-2.4853682564,0.6050339562,-1.2484850421\C,-0.6615682586,-0.7619215579,-0.5356579495\ C,0.5295963207,-0.823668619,-0.3126085536\ N,1.844809697,-0.8539200623,-0.0851331044\ C,2.3005768479,-0.619241721,1.2713416599\N,-1.56721567,1.1499901129,2.3810503105\

N,-0.6438011063,1.7266581387,2.4111399969\ C,2.6405376281,-1.8027490524,-0.8402913951\ N,0.8120250468,2.5730696055,-0.7177610509\ N,1.881865602,2.3944662684,-0.6153632943\ H,3.360176805,-0.3544058261,1.2571258384\ H,1.7394720396,0.2074852888,1.7045266881\ H,2.1727543245,-1.5043269657,1.910420311\ H,-1.7699711548,1.0914660458,-1.9099715059\

H,-3.4232184462,0.4575418421,-1.7891875274\H,-2.680180384,1.2683836082,-0.3947058385\ H,3.6898548045,-1.5003233722,-0.8112361413\ H,2.5626412357,-2.8224580195,-0.4375012395\ H,2.3060526586,-1.810693515,-1.8768308554\H,-3.8194924925,-1.6070793556,-0.6379759018\

H,-2.4538431246,-2.4965869714,0.0620396667\H,-3.1145670319,-1.0773351567,0.9044515466\\

Version=ES64L-G16RevB.01\State=1-A\HF=-563.8898255\RMSD=2.248e-09\RMSF=1.134e-05\ Dipole=-0.0712821,-0.101084,0.2941702\Quadrupole=4.5305884,-2.2820315,-2.2485569,-2.2246626,1.1405789,-0.7019099\PG=C01 [X(C6H12N6)]\\@

**Me2N-CN**

**4g**

HF=-227.2270015 a.u., NIMAG=0

1\1\GINC-R01N15\FOpt\RPBE1PBE\def2TZVP\C3H6N2\WURTHWE\11-Dec-2024\0\\#

pbe1pbe/def2tzvp opt=(maxstep=5) freq pop=nbo emp=gd3bj scrf=(solvent=dichloro-methane)\\dimethylcyanamide\\0,1\

C,-0.6250032235,-0.1911985733,0.1031583756\N,-1.9403752504,-0.1775441999,0.1558644471\

C,-2.6788044271,-0.82695272,-0.9159738721\C,-2.5906479372,0.9369462436,0.8273593858\

H,-2.1230112294,-1.6940831961,-1.2680863536\H,-2.8523299607,-0.1447101158,-1.754825909\

H,-3.6403459946,-1.1609358352,-0.5242416928\H,-1.9748859376,1.2695912026,1.6610190003\

H,-3.5524444339,0.5977852231,1.2139845059\H,-2.7563300747,1.7763121267,0.1438001142\

N,0.5344304692,-0.2322391557,0.0860999988\\ Version=ES64L-G16RevB.01\State=1-A\HF=-227.2270015\RMSD=5.009e-09\RMSF=2.149e-05\Dipole=-2.4114908,0.2568297,-0.1379471\Quadrupole=-5.6957546,2.8548464,2.8409082, 0.4961108,0.0126435,1.6232453\PG=C01 [X(C3H6N2)]\\@

**TS-2g/7g**

HF=-563.7822124 a.u., NIMAG=1, -421.3046 cm^-1^

1\1\GINC-R02N13\FTS\RPBE1PBE\def2TZVP\C6H12N6\WURTHWE\24-Oct-2024\0\\#

pbe1pbe/def2tzvp opt=(ts,noeigentest,calcfc,maxstep=6,maxcycle=200) freq pop=nbo emp=gd3bj scrf=(solvent=dichloromethane)\\TS for N2-elimination of bis-dimethylamino-bis(diazoethane)\\0,1\

N,0.5088368488,-0.5569228928,0.4145257362\N,0.9688479053,0.8900783845,1.7689506658\ N,2.0948225362,0.7562949928,1.9693152914\C,3.2120337229,-0.0640710438,1.649240507\ C,3.1316670524,-0.4861429915,0.3813324163\N,1.4636413721,-1.0067870047,-0.0205176356\ N,4.2680965928,-0.0275204972,2.556623608\C,5.3489291371,0.8708490916,2.1857655951\ N,3.9832054621,-1.0986825834,-0.4234714538\C,5.0250870975,-1.9754058461,0.080765492\ C,3.8756232174,0.1030959726,3.9434762614\C,3.6672752043,-1.2644205318,-1.8246481133\ H,3.2481205226,-2.2569552227,-2.026486397\H,2.9495991274,-0.5073979523,-2.133493237\ H,4.5780960418,-1.149807729,-2.4170806877\H,3.1370524569,-0.6597780481,4.1933610113\

H,4.7527395842,-0.043516025,4.5755460413\H,3.4512210651,1.0917653607,4.1761872449\ H,4.7177519819,-3.0244659875,-0.0090272677\H,5.9432985701,-1.8340378621,-0.4943367883\ H,5.2143534779,-1.7529601212,1.1289313025\H,6.2165883314,0.6799482568,2.8203758464\ H,5.6341272251,0.696339526,1.1476813844\H,5.0616378308,1.9278259985,2.2930774713\\ Version=ES64L-G16 RevB.01\State=1-A\HF=-563.7822124\RMSD=5.115e-09\RMSF=3.831e-06\Dipole=2.2915982,-0.3811291,-0.0796367\Quadrupole=-6.4971659,-0.2326476,6.72 98135, 0.7419191,-0.907001,1.5563646\PG=C01 [X(C6H12N6)]\\@

**TS-3g/7g**

HF=-454.3659515 a.u., NIMAG=1, -573.9808 cm^-1^

1\1\GINC-R10N33\FTS\RPBE1PBE\def2TZVP\C6H12N4\WURTHWE\17-Apr-2025\0\\#

pbe1pbe/def2tzvp opt=(ts,noeigentest,calcfC,maxstep=6) freq pop=nbo emp=gd3bj scrf=(solvent=dichloromethane)\\TS for dimethylamino-carbene-N2-elimination\\0,1\

N,-2.2901017164,2.2371815024,-0.0785301322\N,-1.4871142247,1.5194741369,0.1801024532\

C,-0.4086819128,0.3186557626,-0.1394597722\C,0.8157298832,0.8426138045,0.142780581\

N,-1.0645461286,-0.8526392276,-0.0320356691\C,-1.1200194942,-1.5197518096,1.2562438726\

N,1.938069847,0.1592297472,-0.03768101\C,2.0619883364,-1.1967629984,-0.5483172897\

C,-2.1940462299,-1.102808718,-0.900353834\C,3.2167426489,0.8065027941,0.1596845873\

H,3.7659374435,0.8833548479,-0.7865833669\H,3.0459551229,1.8027669914,0.5591671799\

H,3.8346457718,0.2317460166,0.8583765859\H,-1.9830159209,-0.7043647213,-1.8918317166\

H,-2.3693342033,-2.1775076698,-0.9768174405\H,-3.1152691666,-0.6362696042,-0.5212243672\

H,2.6012570076,-1.193431647,-1.5019929749\H,2.628880268,-1.8173321062,0.1531342881\

H,1.0785582119,-1.6331641007,-0.696931498\H,-1.3046580756,-2.5868296854,1.1151354966\

H,-0.1692292865,-1.3879317594,1.7705445943\H,-1.9210871815,-1.1081435558,1.8857364324

\\Version=ES64L-G16RevB.01\State=1-A\HF=-454.3659515\RMSD=4.007e-09\RMSF=1.453e-06\ Dipole=-0.1511627,-1.863152,-0.042825\Quadrupole=4.4092506,-3.5518366,-0.857414,1.6624817,-0.5478948,-0.5812163\PG=C01 [X(C6H12N4)]\\@

**TS-3g/7g-vdw**

HF=- 563.8155808 a.u., NIMAG=1, -568.2043 cm^-1^

1\1\GINC-R01N27\FTS\RPBE1PBE\def2TZVP\C6H12N6\WURTHWE\17-May-2025\0\\#

pbe1pbe/def2tzvp opt=(ts,noeigentest,calcfc,maxstep=6) freq pop=nbo emp=gd3bj scrf=(solvent=dichloromethane)\\ \\TS for dimethylamino-carbene-N2-elimination -vdw\\0, 1\

C,0.2442136331,-2.7311474672,-0.0554924154\N,0.6147309364,-1.4030872352,0.3990751063\ C,1.8519979091,-1.3335903855,1.1450415329\C,0.2267516582,-0.3301609848,-0.315441083\

C,-0.9137315979,0.1912402914,-0.8437125671\N,-2.071269384,0.1671144975,-0.1991194016\

C,-3.2226044746,0.8450621456,-0.7542761269\N,1.411667872,0.0150184416,-1.3961283839\ N,2.3451914672,0.5256118826,-1.7091171355\C,-2.3194907444,-0.3826743463,1.124264379\ N,1.102532953,2.6202615375,1.0030681444\N,1.9766354157,2.9114157577,0.4208583738\

H,-3.5224683421,1.6874026165,-0.1192910585\H,-2.9680158272,1.212904022,-1.744832012\

H,-4.0745789832,0.1599900505,-0.8246446698\H,1.9543461618,-0.3424792917,1.5849441944\ H,1.8431429628,-2.0783083687,1.9429577721\H,2.727571873,-1.5298946071,0.5089685202\

H,-2.6235934039,0.4136278663,1.8122789205\H,-3.1296032183,-1.1179590956,1.0853258232\

H,-1.4216979242,-0.8600310168,1.5061176486\H,0.2555515041,-3.4287283794,0.784664227\

H,-0.758790186,-2.6995975911,-0.4783688185\H,0.9386997392,-3.0988923398,-0.82342397\\ Version=ES64L-G16RevB.01\State=1-A\HF=-563.8155808\RMSD=4.501e-09\RMSF=2.528e-06\ Dipole=-0.2951429,-1.3444589,1.3325077\Quadrupole=2.3217098,1.740817,-4.0625268,-2.7677332,0.8079622,0.3208244\PG=C01 [X(C6H12N6)]\\@

**7g**

HF=-454.3803243 a.u., NIMAG=0

1\1\GINC-R07N18\FOpt\RPBE1PBE\def2TZVP\C6H12N4\WURTHWE\02-Feb-2025\0\\# pbe1pbe/ def2tzvp opt=(maxstep=5) freq pop=nbo geom=check guess=read emp=gd3bj scrf=(solvent= dichloromethane)\\Bisdimethylamino-carbene 7g\\0,1\

N,-2.1355681316,2.181693671,0.1357168523\N,-1.294700194,1.4437663303,0.0931135703\

C,-0.4196064824,0.4236862913,0.0298332702\C,0.9026089375,0.8960572714,0.0243111929\

N,-1.0665775788,-0.8366641693,-0.0063548109\C,-1.7302641064,-1.2227597406,1.2231060417\

N,1.9391395268,0.0883009817,-0.0272686762\C,1.9530632813,-1.3702878299,-0.0841767928\

C,-1.8584626292,-1.0889463551,-1.1933478296\C,3.2753356189,0.6516555863,-0.0288074828\

H,3.8125613713,0.3557095175,-0.9360673873\H,3.1977798647,1.7344748501,0.0151651764\

H,3.8438193108,0.2861935453,0.832963428\H,-1.2660136924,-0.8670454845,-2.0815691007\

H,-2.1470791942,-2.1420158892,-1.2197467647\H,-2.7816571614,-0.4876070853,-1.2296324162\

H,2.4756639264,-1.6988256689,-0.987633775\H,2.4947257371,-1.7678947354,0.7795170387\

H,0.9363670447,-1.7494144811,-0.0887678606\H,-2.0163073796,-2.2754590545,1.1661412057\

H,-1.0470478815,-1.0920033414,2.0629129722\H,-2.6424425457,-0.6366793375,1.4200568634\\ Version=ES64L-G16RevB.01\State=1-A\HF=-454.3803243\RMSD=6.056e-09\RMSF=5.354e-06\ Dipole=0.067971,-1.7657699,-0.0560596\Quadrupole=4.3353587,-4.6616928,0.3263341,1.03318,-0.0093424,-0.2501381\PG=C01 [X(C6H12N4)]\\@

**7g-vdw**

HF=-563.8299187 a.u., NIMAG=0

1\1\GINC-R01N07\FOpt\RPBE1PBE\def2TZVP\C6H12N6\WURTHWE\16-May-2025\0\\

# pbe1pbe/def2tzvp opt=(maxstep=6) freq pop=nbo emp=gd3bj scrf=(solven

t=dichloromethane)\\Bis-dimethylaminocarbene + N2 vdw\\0,1\

C,0.4604516199,0.2499326422,0.026670614\N,-0.253461648,0.1900602171,1.2865715992\

C ,0.6104968853,-0.0234755893,2.4302923582\C,-1.2472958908,1.185436514,1.4614279147\

C,-2.6475270871,1.2289315077,1.5630376513\N,-3.3915291979,0.1465327826,1.5118239879\

C,-4.8315934817,0.2724705286,1.6270329076\N,-0.7216482444,2.4190299231,1.5588351356\

N,-0.1456733048,3.3765647467,1.6320449409\C,-2.9671671723,-1.2414896621,1.3562700365\

N,-1.4624989441,1.9572126878,4.8913629314\N,-0.9140287691,2.8981853758,4.9208007067\

H,-5.194522464,-0.2880820594,2.4949831314\H,-5.081865111,1.3238541506,1 .7380554208 \

H,-5.3217791712,-0.1327126816,0.7355788326\H,0.0035516442,-0.1692813563,3.3244264528\ H,1.2122409169,-0.9201920743,2.2671902167\H,1.3012928083,0.8165255379,2.6096881134\

H,-3.2978753137,-1.826549396,2.2197997022\H,-3.4307487492,-1.6707663558,0.4629464571\

H,-1.8866198146,-1.2896780158,1.2696734383\H,1.0514798178,-0.6599197721,-0.0989243195\

H,-0.2538723539,0.3183395883,-0.7944170091\H,1.1484525835,1.1086814316,-0.0361425097\\ Version=ES64L-G16RevB.01\State=1-A\HF=-563.8299187\RMSD=7.022e-09\RMSF=3.469e-06\ Dipole=0.4298948,-1.7140758,-0.2313498\Quadrupole=3.5266164,-3.7752384,0.2486221,1.0992784, -0.7211016,1.0768956\PG=C01 [X(C6H12N6)]\\@

**TS-2g/8g**

HF =-563.7162509 a.u., NIMAG=1, -623.7353 cm^-1^

1\1\GINC-R01N02\FTS\RPBE1PBE\def2TZVP\C6H12N6\WURTHWE\07-Mar-2025\0\\#

pbe1pbe/def2tzvp opt=(ts,noeigentest,calcfC,maxstep=3) freq pop=nbo emp=gd3bj scrf=(solvent=dichloromethane)\\TS-search for five-membered ring 8g\\0,1\

N,0.1328410877,2.6680999496,-0.7508080274\N,0.9937677288,1.5979983569,-0.4072108022\

C,0.6653866841,0.3490409632,-0.0716042957\C,-0.730737636,0.2611388526,0.3792161908\

N,-1.1811869851,1.4483378435,0.602883126\N,-0.1928311064,2.3979014853,0.5552752007\

N,1.5184268791,-0.6630385068,-0.2351485563\N,-1.4879416405,-0.8719571,0.4099914924\

C,-1.3885142568,-1.8275616216,-0.6808436121\C,1.579342061,-1.7392851639,0.737384306\

C,-2.8359754867,-0.7024791892,0.912264528\C,2.7150198505,-0.4378884301,-1.0164314175\

H,-2.0303045512,-1.5329172039,-1.5201705035\H,-0.3659647728,-1.900895329,-1.0416616867\

H,-1.7070209325,-2.8078786779,-0.3247891621\H,0.6413502226,-1.8024566385,1.2838875167\

H,1.7624699651,-2.6859943044,0.2276240137\H,2.3923365695,-1.557204915,1.4483054669\

H,-3.4781738115,-0.1770743025,0.1944971628\H,-3.2578940474,-1.688632257,1.1069675647\

H,-2.8137267122,-0.1301848729,1.8387393034\H,3.1685835715,-1.4015063238,-1.2447847379\

H,2.4674898842,0.0716335758,-1.9456275468\H,3.4353204352,0.1737478097,-0.460533524\\ Version=ES64L-G16RevB.01\State=1-A\HF =-563.7162509\RMSD=9.249e-09\RMSF=2.134e-05\Dipole=0.4515529,-4.079654 6,0.0928499\Quadrupole=9.1276885,-9.1492591,0.0215705,-1.0217251,-1.1822719,1.9800084\PG=C01 [X(C6H12N6)]\\@

**8g**

HF=-563.7886723 a.u., NIMAG=0

1\1\GINC-R02N02\FOpt\RPBE1PBE\def2TZVP\C6H12N6\WURTHWE\15-Mar-2025\0\\

# pbe1pbe/def2tzvp opt=(maxstep=6) freq pop=nbo emp=gd3bj scrf=(solvent=dichloro methane)\\Five-membered ring 8g\\0,1\

N,1.7226337479,-0.2849446268,0.2987120117\N,-1.4070422811,-0.8410126204,0.1267780966\

C,-0.6369288101,0.3024044713,-0.0773112447\C,0.6945625818,0.5084051383,0.0029597956\

C,1.5347571396,-1.6805580104,0.6246289921\C,-2.2850984463,-0.7870446091,1.2840811582\

N,-1.3074831927,1.5185345051,-0.4414500442\N,1.0546862006,1.9065947382,-0.3047238786\

N,0.1307710137,2.6197045422,-0.5703525844\N,-2.4039076105,1.828780482,-0.6252383513\

C,-2.0529511898,-1.3628160802,-1.0664273821\C,3.0990495284,0.1649946277,0.3232669117\

H,0.4777157401,-1.9305023863,0.5803694871\H,1.9185375927,-1.8797652371,1.6296293507\

H,2.090360491,-2.3027468291,-0.0832581447\H,-2.69148999,-1.7846607871,1.4642832715\

H,-1.7101701972,-0.4846141979,2.1605180214\H,-3.1247184166,-0.0915067839,1.1545382314\

H,-1.3128684852,-1.4704498719,-1.8606954076\H,-2.8692049447,-0.7235506876,-1.4277870759\

H,-2.4644653937,-2.3490801822,-0.8410614082\H,3.5180987213,0.0055279256,1.3208949117\

H,3.164526751,1.2174634027,0.0704252167\H,3.6865964499,-0.4167039231,-0.3927509347

\\Version=ES64L-G16RevB.01\State=1-A\HF=-563.7886723

\RMSD=5.027e-09\RMSF=7.602e-06\Dipole=1.547686,-2.5884238,0.7871928\Qu

adrupole=3.3261404,-5.7998535,2.4737131,2.5875787,-0.6127007,2.4431862

\PG=C01 [X(C6H12N6)]\\@

**TS 2g-35**

HF=-563.733841 a.u., NIMAG=1, -128.1469 cm^-1^

1\1\GINC-R08N40\FTS\RPBE1PBE\def2TZVP\C6H12N6\WURTHWE\15-Dec-2024\0\\#

pbe1pbe/def2tzvp opt=(ts,noeigentest,calcfC,maxstep=6) freq pop=nbo emp=gd3bj scrf=(solvent=dichloromethane)\\TS for five-membered ring from bisdimethylaminotetrazine\\0,1\

C,0.5672754266,0.0523315823,0.0442180346\N,0.2726145505,0.1419329238,1.4567275084\

C,1.3858269916,0.4494826935,2.3369840147\C,-0.8862739553,-0.2818814649,1.9193564151\

N,-1.2943715921,-0.3551602242,3.0779861797\N,-1.9150856101,-0.4927016753,4.1318600611\

N,-1.729540968,-1.7697225849,4.7756689866\N,-1.1360565521,-2.6328532615,4.1022980674\

C,-0.4307532875,-2.7683830431,3.0569147691\N,-0.6823907013,-3.5076410468,2.0093146418\

C,-2.0257141089,-3.9565684676,1.6805929146\C,0.3261467717,-3.7015328021,0.9901183069\

H,-1.9742187963,-4.9332207177,1.1974118452\H,-2.613368839,-4.0346649259,2.594015595\

H,-2.512288367,-3.2517154084,0.9997796069\H,1.0086365369,0.7850169204,3.3016579931\ H,2.0217343428,-0.4283770377,2.4888773616\H,1.9863699828,1.2454445653,1.8940424036\

H,0.4223200096,-4.7642078761,0.7546066152\H,0.0526852685,-3.1664667851,0.0751854817\

H,1.2770203799,-3.3222731498,1.3567672878\H,1.3096258977,-0.7291194215,-0.1509315289\

H,-0.3469710662,-0.1841373393,-0.4955166178\H,0.9646388766,1.0035310689,-0.3187369313\\ Version=ES64L-G16RevB.01\State=1-A\HF=-563.733841\RMSD=4.627e-09\RMSF=1.953e-06\ Dipole=1.6721215,-0.570688,-3.1099986\Quadrupole=0.8023194,6.1138373,-6.9161567,6.99907

16,3.0659064,1.3620098\PG=C01 [X(C6H12N6)]\\@

**35**

HF=-563.8316778 a.u., NIMAG=0

1\1\GINC-R03N22\FOpt\RPBE1PBE\def2TZVP\C6H12N6\WURTHWE\21-Mar-2025\0\\

# pbe1pbe/def2tzvp opt=(maxstep=6) freq pop=nbo emp=gd3bj scrf=(solven

t=dichloromethane)\\ \\five-membered ring from bisdimethylaminotetrazine \\0,1\

C,0.2037520621,2.4220433889,-2.4814931337\N,0.332838906,1.6277397743,-1.2693859436\

C,1.6945786891,1.1648309989,-1.0157224054\C,-0.7350996978,1.4504933608,-0.548761777\

N,-0.5143054885,0.5914744363,0.5669553763\N,-0.9539294306,1.0152214672,1.7762423074\

N,-0.6898579956,0.090305285,2.6107735541\N,-0.1625518063,-0.987610762,2.0105059546\

C,-0.0609348829,-0.6819799106,0.7283986684\N,0.4130746792,-1.4935181283,-0.2503840156\

C,-0.3318334432,-1.6062776837,-1.4928434568\C,0.9917216663,-2.7442310904,0.2004513805\

H,-1.1264610853,-2.3572693046,-1.4076083206\H,-0.7885600095,-0.6575496714,-1.7668399237\

H,0.3470989142,-1.9062033664,-2.2921804662\H,1.8206786664,0.9154622096,0.0337051174\

H,1.9154759996,0.2875808868,-1.6253343619\H,2.3817762654,1.9670380119,-1.2830145835\

H,0.2259711823,-3.4627689313,0.5161879411\H,1.5585108363,-3.1742533581,-0.6258719117\

H,1.6630989202,-2.5615805909,1.0372471831\H,0.5080184949,1.8230222109,-3.3424580828\

H,-0.8332949152,2.726389289,-2.5874695691\H,0.8528134727,3.297156478,-2.4163105313\\ Version=ES64L-G16RevB.01\State=1-A\HF=-563.8316778\RMSD=9.097e-09\RMSF=1.537e-05\Dipole=2.1598277,-0.523368,-3.6970868\Quadrupole=0.0879285,5.5764089,-5.6643374, 5.1790054,1.6969093,-2.3068602\PG=C01 [X(C6H12N6)]\\@

Table S8 Species 25-34**:** Total energies (E_tot_) and Gibbs free energies (G_298_) [a.u.] of compounds **25-34** and of the related transition states as given in the Gaussian archive entries. The relative energies [kcal/mol] include all involved species according to the respective reactions as given in the schemes in the manuscript.

| **Species** | **E_tot_ [a.u.]** | **E_rel_ [kcal/mol]** | **G_298_ [a.u.]** | **E_rel_ [kcal/mol]** |
| --- | --- | --- | --- | --- |
| **24** | -1202.58768 | 0.00 | -1.202.33676 | 0.00 |
| **TS-24/25** | -1202.55717 | 19.14 | -1.202.30248 | 21.51 |
| **25** | -1202.66061 | -45.77 | -757.63680 | -39.21 |
| **26** | -759.03056 | 0.00 | -757.62539 | 0.00 |
| **TS-26/27** | -758.99208 | 24.15 | -757.71951 | 24.77 |
| **27** | -759.05265 | -13.86 | -757.63680 | -10.73 |
| **28** | -757.80306 | 18.78 | -757.62539 | 18.74 |
| **TS-28/29** | -757.79306 | 25.06 | -757.71951 | 25.90 |
| **29** | -757.89038 | -36.01 | -757.63680 | -33.16 |
| **30** | -972.65189 | 0.00 | -972.40080 | 0.00 |
| **TS-30/31** | -972.65014 | 1.10 | -972.39674 | 2.55 |
| **31** | -972.75211 | -62.89 | -972.49296 | -57.83 |
| **32** | -1187.47071 | 0.00 | -1187.13461 | 0.00 |
| **TS-32/33** | -1187.46358 | 4.48 | -1187.12615 | 5.30 |
| **33** | -1187.62136 | -94.54 | -1187.13461 | -87.85 |

**24**

HF=-1202.5876751 a.u., NIMAG=0

1\1\GINC-R08N44\FOpt\RPBE1PBE\def2TZVP\C16H18N2O1S1\WURTHWE\12-May-202

1\0\\# pbe1pbe/def2tzvp opt=(maxstep=8) freq pop=nbo emp=gd3bj nosym scrf=(solvent= dichloromethane)\\open compound 25\\0,1\

C,-0.1041604332,-0.2890018622,0.2411603554\C,0.3972075559,-0.0966780636,1.5318798922\

C,1.6450534184,0.5202072261,1.678444045\C,2.3642844132,0.9249151255,0.5680258126\

C,1.8564444111,0.7347203962,-0.7115011859\C,0.618433339,0.1271673894,-0.865375052\

C,-0.4026599343,-0.5276806213,2.675336528\C,-1.7973163719,-0.9073197253,2.5949266171\

S,-2.2946924003,-2.280400568,3.3530999822\C,-3.9009952425,-2.4473455163,3.4270174219\

C,-5.1083765205,-1.6313868594,3.0068465434\C,-5.2820242479,-0.292348957,3.7163543731\

N,0.1667653722,-0.5051818405,3.850180477\N,0.6795850283,-0.5091066766,4.8590470387\

C,-4.6952916542,-3.6011666364,4.0275342692\C,-4.6146002039,-4.9305393784,3.2839113499\

C,-5.9284855512,-2.7688403076,3.6387218168\O,-7.1036412758,-2.940249865,3.7827284079\

C,-4.5505434449,-3.8027165129,5.531451802\C,-5.3449347388,-1.498332192,1.5048267287\

H,2.0559039226,0.6912385292,2.6673485842\H,3.3298525016,1.3988227226,0.703748742\

H,2.4214633185,1.0563189858,-1.5782079728\H,0.2111813562,-0.0323691236,-1.8574574287\

H,-1.0615832859,-0.779892159,0.1120731851\H,-2.4987400829,-0.2841320059,2.054567707\

H,-6.282958157,0.1001030108,3.5244392225\H,-5.1473336261,-0.3909193014,4.794667453\

H,-4.555345753,0.4337293924,3.3458634579\H,-6.3623023728,-1.1451706643,1.3215919925\

H,-4.6511723995,-0.7744145546,1.0718709924\H,-5.2093953918,-2.4507679794,0.9899284755\

H,-5.3920624373,-5.610184825,3.6402904775\H,-4.7348016184,-4.7968679554,2.2075419331\

H,-3.6419011809,-5.394630898,3.46403897\H,-5.3239223918,-4.4818435638,5.8972879427\

H,-3.5750523199,-4.2416928895,5.7543380683\H,-4.6315283075,-2.8576397741,6.0710583601\\ Version=ES64L-G16RevB.01\HF=-1202.5876751\RMSD=4.391e-09\RMSF=6.216e-06\Dipole= 0.2516751,0.010927,-0.6074538\Quadrupole=-10.7546878,8.2997674,2.4549204,-2.9626195,

3.8760615,-0.4193539\PG=C01 [X(C16H18N2O1S1)]\\@

**TS-24/25**

HF=-1202.5571703 a.u., NIMAG=1, -229.7314 cm^-1^

1\1\GINC-R08N09\FTS\RPBE1PBE\def2TZVP\C16H18N2O1S1\WURTHWE\17-May-2021

\0\\# pbe1pbe/def2tzvp opt=(ts,noeigentest,maxstep=8,calcfc) freq pop=nbo emp=gd3bj nosym scrf=(solvent=dichloromethane)\\TS for ring closure of 25 to give 26\\0,1\

C,-0.017003775,-0.0075998092,-0.0004710791\C,-0.0083629468,-0.0035649801,1.3884784239\

C,1.1899713035,0.0041911307,2.0815959297\C,2.4085505422,0.0221649319,1.3957962495\

C,2.3904129791,0.0274118725,-0.0036467453\C,1.1897288145,0.0040853278,-0.6903730028\

C,3.63822412,0.0272576332,2.1581518668\C,3.8999945168,0.3188830178,3.5054095549\

S,4.9709105965,-0.9225794946,3.9994879504\C,6.5367433655,-0.3829255211,3.7888185491\

C,7.2168529705,0.9697151501,4.0468043876\C,8.5097237233,0.1585353646,4.2081669834\

O,9.6526573418,0.4714737687,4.368842496\N,4.8339098292,-0.044185317,1.4519058516\

N,5.9774515395,-0.1301825138,1.441059709\C,7.8031747553,-1.1954236922,4.0671545785\

C,7.7996428669,-2.016755802,5.3527357067\C,8.3016194445,-2.0257407215,2.8899283928\

C,6.7931308482,1.5864369243,5.3836898445\C,7.2472568018,2.030633035,2.9589929282\

H,3.3258028719,0.0649728522,-0.5513219874\H,1.1939751325,0.0065167922,-1.7745257567\

H,-0.9555533458,-0.0191524041,-0.5419354744\H,-0.9436906651,-0.0164881905,1.9365958954\

H,1.2023843638,-0.0191618092,3.1655012002\H,3.7957621725,1.2608645321,4.0310160774\

H,7.9021216064,2.842444677,3.2820324101\H,7.6204630141,1.6392638993,2.0144291992\

H,6.2487898132,2.4405598898,2.7959507576\H,7.5054611451,2.3651382891,5.6643191289\

H,5.8044416925,2.0372816635,5.2812966274\H,6.7518710562,0.8476212681,6.1849302971\

H,8.8106004017,-2.3700811139,5.5648627087\H,7.4488643656,-1.4370218939,6.2076595989\

H,7.1484864724,-2.8857678834,5.2351847136\H,9.3008607532,-2.4074506985,3.1093481223\

H,7.6312308828,-2.8724774693,2.7284292578\H,8.3396145194,-1.4378767399,1.9734887464\\ Version=ES64L-G16RevB.01\HF=-1202.5571703\RMSD=5.500e-09\RMSF=1.825e-06\Dipole=-0.4793462,-0.0546317,0.4565609\Quadrupole=-16.9919735,4.2212094,12.770764,-5.0829922, 1.591908,-0.6916671\PG=C01 [X(C16H18N2O1S1)]\\@

**25**

HF= -1202.6606138 a.u., NIMAG=0

1\1\GINC-R09N16\FOpt\RPBE1PBE\def2TZVP\C16H18N2O1S1\WURTHWE\15-Feb-202

0\0\\# pbe1pbe/def2tzvp Opt=(maxstep=8,readfc) geom=check guess=read Pop=NBO Freq emp=gd3bj nosym scrf=(solvent=dichloromethane)\\Six-membered ring 26\\0,1\

N,-2.2308542831,-1.7656836403,2.2145857764\C,-1.7594510288,-2.7011593463,3.2436393109\

S,0.0253164193,-2.5856728294,3.2386761334\C,0.216719055,-2.9562793518,1.5859664335\

C,-0.8086553503,-2.806735599,0.7074596305\N,-1.8437772549,-1.9442875894,1.0494184301\

C,-2.4022721241,-2.4492085777,4.6500778581\C,-3.5542644719,-1.4527716016,4.6426305398\

C,-2.394664322,-4.1571316192,3.1842469872\C,-3.5565680764,-4.2989887985,2.2064046944\

C,-0.7567319602,-3.2586357711,-0.6852442877\C,-1.4779676892,-2.6052328026,-1.6868416966\

C,-1.4212514865,-3.0531274457,-2.9960387798\C,-0.6476068534,-4.1574223644,-3.3290993437\

C,0.0640034742,-4.8187958414,-2.3378285328\C,0.0033684498,-4.3789024319,-1.0255183397\

C,-2.8923110184,-3.8884713446,4.6005371354\O,-3.4465490007,-4.5853300782,5.4025251505\

C,-1.4611490274,-2.1663590307,5.8092392251\C,-1.4729492452,-5.3595756594,3.0993728146\

H,-2.0728003405,-1.7385379624,-1.4289784304\H,-1.982228757,-2.5329401789,-3.7640344297\

H,-0.6064188067,-4.5055967469,-4.3545321338\H,0.658305368,-5.6910425553,-2.5843551045\

H,0.5338189989,-4.9223844667,-0.2515059254\H,1.2139622147,-3.2309175252,1.2637377493\

H,-4.1085923588,-5.2098631446,2.4455478852\H,-4.2487549901,-3.4578099189,2.2510491515\

H,-3.1855884959,-4.38001445,1.1827004859\H,-2.0309153893,-6.2476259023,3.4037986944\

H,-1.1203876884,-5.5078109936,2.0776408671\H,-0.6072582698,-5.263554096,3.755400814\

H,-2.0235829156,-2.1982513865,6.7438765215\H,-0.6552596307,-2.8994190487,5.8812866622\

H,-1.0210763611,-1.1713267165,5.7105374472\H,-4.0842585445,-1.5235079648,5.5943071556\

H,-3.1714204515,-0.4375283786,4.5268027843\H,-4.2616988424,-.6364867768,3.834626449\\ Version=ES64L-G16RevB.01\HF=-1202.6606138\RMSD=5.862e-09\RMSF=1.475e-06\Dipole= 1.1444287,-0.382703,-0.6216292\Quadrupole=-3.5533183,4.4433261,-0.8900078,-10.9119578,10.

0066762,11.4132912\PG=C01 [X(C16H18N2O1S1)]\\@

**26**

HF=-759.0305594 a.u. NIMAG=0

1\1\GINC-R07N13\FOpt\RPBE1PBE\def2TZVP\C14H12N4\WURTHWE\23-May-2021\0\

\# pbe1pbe/def2tzvp opt=(maxstep=5,gdiis) freq pop=nbo emp=gd3bj scrf= (solvent= dichloromethane)\\open chain compound 27\\0,1\

C,-0.2491234669,0.0710817023,0.0934727462\C,-0.1306825315,0.0215212848,1.4936559431\

C,1.1488858653,0.1891983378,2.0496465705\C,2.2480433984,0.4011971059,1.230878152\

C,2.1127701446,0.4488915944,-0.1477758432\C,0.8527688487,0.2811847578,-0.7113110448\

C,-1.3317138671,-0.1846864879,2.277094214\N,-1.3645233593,-0.2946932145,3.6007640082\

N,-0.3926690725,-0.2817009679,4.4565653417\C,-2.6534601433,-0.2708289759,1.6320048999\

N,-3.1203668391,-1.4721213235,1.4369306011\N,-3.5307636345,-2.5102489551,1.2755398113\

C,-3.4098434553,0.8645926037,1.1162325139\C,-2.9227629586,2.1566568195,1.3348286874\

C,-3.6109605465,3.2589316124,0.8560225085\C,-4.7980279619,3.0999385166,0.1545517072\

C,-5.2888554837,1.8192759043,-0.0654857768\C,-4.6051414952,0.7117518007,0.4053892366\

H,3.2254057601,0.5279719597,1.6831491451\H,1.2654989274,0.1481117512,3.1215325327\

H,-1.2237744121,-0.0554833052,-0.3618639472\H,0.7287504793,0.3141682811,-1.7877246872\

H,2.977944186,0.6126307206,-0.7796212131\H,-3.2139663599,4.2517945319,1.034658976\

H,-1.9968587301,2.2912544903,1.8818335424\H,-5.0062582537,-0.2779604039,0.2165541924\

H,-6.2143006687,1.679108794,-0.61246639\H,-5.3352603899,3.9638107757,-0.2180218925\

H,-2.3175437475,-0.4037645805,3.9480028078\H,-0.8247228747,-0.3854372979,5.3708393142\\ Version=ES64L-G16RevB.01\State=1-A\HF=-759.0305594\RMSD=8.483e-09\RMSF=8.123e-06\ Dipole=-0.952791,0.3310438,0.3413108\Quadrupole=-0.3015643,-5.0841094,5.3856737, 0.53138,-3.8422509,-2.2342207\PG=C01 [X(C14H12N4)]\\@

**TS-26/27**

HF=-758.9920815 a.u. NIMAG=1, -241.3950 cm^-1^

1\1\GINC-R07N13\FTS\RPBE1PBE\def2TZVP\C14H12N4\WURTHWE\24-May-2021\0\\

# pbe1pbe/def2tzvp opt=(ts,noeigentest,calcfC,maxstep=6) freq pop=nbo

emp=gd3bj scrf=(solvent=dichloromethane)\\TS for ring closure of 27 to give 28\

C,-0.0002804169,0.0034461148,-0.0012208944\C,-0.0020323344,-0.0000569727,1.3991361751\

C,1.2257157916,-0.0000382291,2.0704725216\C,2.4153611459,-0.0002108311,1.3633242182\

C,2.4056529761,0.0107270686,-0.0254265025\C,1.1910861383,0.0158896746,-0.7023445477\

C,-1.2397455412,-0.0593874331,2.1446323059\N,-2.422525225,0.4495124022,1.6367871665\

N,-2.5237822599,1.7259329036,1.3775098847\C,-1.4433883479,-0.1138259162,3.5334159822\

N,-1.9265171242,1.136765747,3.898915153\N,-2.333132887,2.1507325326,3.5370664793\

C,-1.0363188708,-1.0473337809,4.5594019037\C,-1.0350204134,-0.713326303,5.9169643292\

C,-0.6458970262,-1.6412403286,6.8676943685\C,-0.2385781719,-2.9135336728,6.486408303\

C,-0.2329138706,-3.2513535615,5.138870828\C,-0.6344848361,-2.3331204701,4.1844335312\

H,1.174975926,0.0218683167,-1.7862758523\H,-0.9440993709,-0.0113301123,-0.5336091553\

H,1.2368894723,0.0098521759,3.1542415879\H,3.3574267159,0.0006806161,1.8995914384\

H,3.3378323067,0.0154120634,-0.5779513022\H,0.0790777105,-4.2423325309,4.8291296615\

H,-0.6467511078,-2.6044323869,3.1345919981\H,-1.3299518292,0.2844006426,6.2229265623\

H,-0.6521221657,-1.3655906955,7.9162111914\H,0.0709283756,-3.6352609784,7.2329530425\

H,-3.247956613,-0.01595893,2.012125311\H,-3.5037626401,1.9231540914,1.1742970166\\ Version=ES64L-G16RevB.01\State=1-A\HF=-758.9920815\RMSD=5.195e-09\RMSF=9.146e-06\Dipole=-0.6480935,-0.8997921,-0.2164108\Quadrupole=6.6039638,-10.1632248,3.559261,-0.9553209,3.0902531,0.2052949\PG=C01 [X(C14H12N4)]\\@

**27**

HF=-759.0526536 a.u., NIMAG=0

1\1\GINC-R01N31\FOpt\RPBE1PBE\def2TZVP\C14H12N4\WURTHWE\24-May-2021\0\

\# pbe1pbe/def2tzvp opt=(maxstep=6) freq pop=nbo emp=gd3bj scrf=(solvent=dichloromethane)\\ Six-membered ring product 28\\0,1\

C,-0.0320209091,-0.0724852165,-0.0162026816\C,-0.0143151685,-0.0078704272,1.3789579695\

C,1.2126628158,0.0498608255,2.0418288037\C,2.3985531051,0.0301050397,1.3243275422\

C,2.3717823508,-0.0448318867,-0.0603307729\C,1.1529050618,-0.0929404988,-0.7279220591\

C,-1.2570424725,0.0454374992,2.1493016117\C,-2.4786782694,-0.4831710178,1.8159517543\

N,-3.5834970914,0.287670205,2.1964919269\N,-3.5664285238,0.9648637478,3.2268514463\

N,-2.4094680479,0.6880450389,4.0236724579\N,-1.2411363939,0.8429770591,3.2650247847\

C,-2.7246815913,-1.5787993748,0.881628643\C,-3.8347607342,-1.573397443,0.033671061\

C,-4.0558612106,-2.6231028549,-0.8434350767\C,-3.1770938615,-3.6968676503,-0.8917774541\

C,-2.0782643442,-3.7174134385,-0.042260821\C,-1.8588447998,-2.6745418312,0.8412198109\

H,1.1285259655,-0.1391893136,-1.8103073939\H,-0.9810799359,-0.0962534265,-0.5369152509\

H,1.2279466433,0.0895880663,3.1244749697\H,3.3458593249,0.0667112618,1.8493442077\

H,3.2986854054,-0.0631769923,-0.6216426651\H,-1.3935702414,-4.5577646291,-0.0594192184\

H,-1.0114624915,-2.7067205409,1.5162335181\H,-4.5167403863,-0.732804479,0.0619627699\

H,-4.9193711592,-2.5993509581,-1.498685885\H,-3.3514867659,-4.515927614,-1.5797113602\

H,-0.9260963123,1.8019792681,3.1079745484\H,-2.3952185582,1.3686169928,4.7732385003\\ Version=ES64L-G16RevB.01\State=1-A\HF=-759.0526536\RMSD=7.688e-09\RMSF=8.441e-06\Dipole=1.9732569,0.6427423,-0.1413501\Quadrupole=-3.5669595,0.5475557,3.0194037, 3.5821082,4.9241028,7.0439832\PG=C01 [X(C14H12N4)]\\@

**28**

HF=-757.8030637 a.u., NIMAG=0

1\1\GINC-R01N14\FOpt\RPBE1PBE\def2TZVP\C14H10N4\WURTHWE\18-Feb-2025\0\

\# pbe1pbe/def2tzvp opt=(maxstep=8,maxcycle=500) freq pop=nbo emp=gd3bj scrf=(solvent=dichloromethane)\\open chain compound 29\\0,1\

N,-3.0723914554,2.6443897938,-0.618728835\N,-2.3791198904,2.5975181309,0.3892821539\

N,-3.7450711622,2.8168471814,-1.5027737068\C,-1.7568095384,1.3696050355,0.7214577832\

N,-0.5994462063,1.4566421513,1.2458915124\C,0.4183440785,1.4958415117,1.9230729786\

C,-2.4281866099,0.0802454906,0.5677836127\C,-3.8198420887,-0.0066411621,0.4785355221\

C,-4.4375479839,-1.2411530502,0.3587428414\C,-3.683173278,-2.4048749438,0.3296146366\

C,-2.2990745808,-2.3263809338,0.4249671975\C,-1.675054324,-1.0977068702,0.5392746625\

C,2.8426421772,1.3489713213,2.2677709379\C,1.7701364695,1.4063451047,1.3787982023\

C,2.016534229,1.379439581,0.0015795928\C,3.3125128678,1.2941698185,-0.4709915174\

C,4.3752234925,1.2306344391,0.4250492977\C,4.1411839561,1.2563455965,1.7926279722\

H,-4.4306497514,0.8858063145,0.5342338362\H,-5.5183183935,-1.2917362601,0.2970604013\

H,-4.1697907412,-3.3681641334,0.2338652506\H,-1.7005345466,-3.2296053159,0.4015096771\

H,-0.5944175988,-1.0423113117,0.5967332583\H,2.639543643,1.3762370622,3.3318500886\

H,1.1823094474,1.4278591788,-0.6897410037\H,3.5012184503,1.2755434982,-1.5380517478\

H,5.3906664685,1.1628141283,0.0518557178\H,4.9711138698,1.2081856426,2.4874076768\\ Version=ES64L-G16RevB.01\State=1-A\HF=-757.8030637\RMSD=9.125e-09\RMSF=2.711e-06\Dipole=0.9185228,-0.915783,-0.5667039\Quadrupole=11.5519617,-3.7956058,-7.7563559, 4.0778368,-2.2912011,0.0094699\PG=C01 [X(C14H10N4)]\\@

**TS-28/29**

HF=-757.7930562 a.u., NIMAG=1, -295.4569 cm^-1^

1\1\GINC-R09N44\FTS\RPBE1PBE\def2TZVP\C14H10N4\WURTHWE\19-Feb-2025\0\\

# pbe1pbe/def2tzvp opt=(ts,noeigentest,calcfC,maxstep=5) freq pop=nbo emp=gd3bj scrf=(solvent=dichloromethane)\\TS for ring closure of 29 to give 30\\0,1\

N,0.055694561,2.4852385095,0.2557940078\N,0.9101673851,1.8054441426,0.8796569756\

N,-0.8381925673,2.6889433169,-0.4255759539\C,1.0498474202,0.5881806865,0.1549944226\

N,-0.0187160897,0.1310788832,-0.4013574431\C,-1.1596120262,0.4474270401,-0.7813653151\

C,2.357883615,-0.0297060818,0.0407199564\C,3.4930622609,0.6181433512,0.5298523412\

C,4.7355444417,0.012331009,0.4344961492\C,4.8629871851,-1.2382947889,-0.1531103727\

C,3.7350221496,-1.8856999999,-0.6453700487\C,2.4910247941,-1.2910543486,-0.5477167482\

C,-3.6053051722,0.3787940933,-0.7989358266\C,-2.4011169526,-0.1485572046,-0.3303093396\

C,-2.4196360196,-1.2290119028,0.5603457211\C,-3.6245942572,-1.7679353742,0.9690565768\

C,-4.8197800008,-1.2279932897,0.5046295647\C,-4.8108925606,-0.1535663785,-0.374827792\

H,3.3929017575,1.600455478,0.9739134047\H,5.6110189636,0.5243565413,0.8166439991\

H,5.8363989062,-1.708355865,-0.2288822291\H,3.8271772779,-2.8632553646,-1.1042692972\

H,1.6094205296,-1.8016810199,-0.9177872299\H,-3.576290051,1.2134873943,-1.4891032444\

H,-1.4818044929,-1.6381531201,0.9182553738\H,-3.6388727366,-2.6067342018,1.6550145597\

H,-5.7633047984,-1.6504858621,0.8304897901\H,-5.7443765224,0.2629793567,-0.7335560024\\ Version=ES64L-G16RevB.01\State=1-A\HF=-757.7930562\RMSD=6.896e-09\RMSF=4.573e-06\Dipole=-0.6126941,-1.1380783,0.237647\Quadrupole=13.3739853,-4.0751166,-9.2988687, 1.4955233,-1.8211306,-0.5323469\PG=C01 [X(C14H10N4)]\\@

**29**

HF=-757.8903784 a.u., NIMAG=0

1\1\GINC-R01N27\FOpt\RPBE1PBE\def2TZVP\C14H10N4\WURTHWE\18-Feb-2025\0\

\# pbe1pbe/def2tzvp opt=(maxstep=8,maxcycle=500) freq pop=nbo emp=gd3bj scrf=(solvent=dichloromethane)\\Six-membered ring product 30 (1,2,3,5-tetrazine\\0,1\

C,-1.4853837889,-1.857872154,-1.2861269409\C,-2.1970819318,-0.6686440339,-1.1245013907\

C,-3.5710683854,-0.6480262552,-1.3680365854\C,-4.220655674,-1.8028195856,-1.7675265301\

C,-3.5076456176,-2.9840017867,-1.9269428504\C,-2.1397640011,-3.0093667447,-1.6856724909\

C,-1.4969812748,0.5485469004,-0.7006250713\N,-0.1923564898,0.4977831023,-0.4770473277\

N,-0.336008326,2.7797469416,0.0543386282\N,-1.6165099318,2.7586321775,-0.1824801571\

N,-2.2245361536,1.6699491001,-0.558370481\C,1.8016449146,1.6812169337,0.1726857534\

C,2.5678660115,0.5239931166,0.0291152994\C,3.9274239806,0.5560160001,0.2828438732\

C,4.5329538234,1.7411279056,0.6813253079\C,3.7747272165,2.8957858342,0.8257214308\

C,2.4143402773,2.8693873043,0.5732315914\H,-0.4200745603,-1.8650609083,-1.0951770734\

H,-4.1174959141,0.277060341,-1.2410006753\H,-5.2874429706,-1.7827755648,-1.9556331879\

H,-4.0189564729,-3.8869716132,-2.2399231376\H,-1.5831778463,-3.9305959483,-1.8099252916\

H,2.0847460578,-0.3932114159,-0.2818942338\H,4.5185473861,-0.3449564384,0.1700973693\

H,5.5982369387,1.7646639274,0.8797395323\H,4.2467287696,3.8201595453,1.1364767318\

H,1.8149931806,3.7634111544,0.6828139239\C,0.3606667824,1.6401991645,-0.0979820166\\ Version=ES64L-G16RevB.01\State=1-A\HF=-757.8903784\RMSD=5.735e-09\RMSF=2.236e-06\Dipole=1.0797848,-1.7140714,-0.2232889\Quadrupole=9.1910182,-2.0903456,-7.1006725,

10.0644888,5.7476454,3.5036603\PG=C01 [X(C14H10N4)]\\@

**30**

HF=-972.651894 a.u., NIMAG=0

1\1\GINC-R03N40\FOpt\RPBE1PBE\def2TZVP\C21H15N3\WURTHWE\22-Jan-2025\0\

\# pbe1pbe/def2tzvp opt=(maxstep=5) freq pop=nbo emp=gd3bj scrf=(solvent=dichloromethane)\\open chain compound 31\\0,1\

C,-0.0184932914,-0.026466841,0.0113049136\C,0.0028614294,0.0020581377,1.4086003061\

C,1.2407046677,0.012142269,2.0595449443\C,2.417804945,-0.0127356047,1.3320174511\

C,2.3888874504,-0.035364346,-0.0565589176\C,1.1640429052,-0.0402769361,-0.7094443325\

C,-1.253232731,0.017718322,2.1473270349\N,-1.2496587821,-0.2962984977,3.4102388624\

N,-1.30492948,-0.5980077875,4.4969014666\C,-2.5887868713,0.2553837308,1.5829098139\

C,-2.90928639,1.4530278981,0.8078192801\C,-4.0943981989,1.5295663975,0.0692525843\

C,-4.4045806599,2.6727931108,-0.6457370359\C,-3.5365647386,3.7579127362,-0.6475356291\

C,-2.3557367896,3.6882992588,0.0789210503\C,-2.0427300624,2.5489275791,0.8014658657\

N,-3.5113531028,-0.5961737068,1.8621215877\C,-4.3877348408,-1.3084014544,2.305646228\

C,-4.9259748767,-2.5161909481,1.6948784737\C,-6.0454595862,-3.1198866101,2.2651435889\

C,-6.5795314029,-4.2692520839,1.7048158591\C,-5.9891096907,-4.8285601283,0.580262394\

C,-4.8661225852,-4.238035238,0.009713488\C,-4.3374971216,-3.0855864439,0.5604120186\

H,1.1257199808,-0.0607646509,-1.792663596\H,3.3122106413,-0.0484145613,-0.6232000725\

H,3.3669559379,-0.0066808011,1.8556788529\H,1.284817866,0.0468206397,3.1426265814\

H,-0.9686288063,-0.0410541794,-0.5088428867\H,-1.6729650442,4.5301959134,0.0878775468\

H,-1.1243692231,2.5078373473,1.3744562461\H,-4.7699905516,0.6819898981,0.0521087931\

H,-5.3279983817,2.7151914591,-1.2119709443\H,-3.7785723521,4.6499457937,-1.2131214723\

H,-6.4874041969,-2.6740780875,3.1483410449\H,-7.4528272616,-4.7328000652,2.1478401821\

H,-6.4027356074,-5.7311141657,0.1452836021\H,-4.4073069037,-4.6796548431,-0.8672214788\

H,-3.4639618124,-2.616367249,0.1213110261\\

Version=ES64L-G16RevB.01\State=1-A\HF=-972.651894\RMSD=2.680e-09\RMSF=1.256e-06\Dipole=-0.197925,-0.5996825,-1.24386\Quadrupole=5.3723399,3.3563403,-8.7286802, 8.7291266,2.0700363,1.3705724\PG=C01 [X(C21H15N3)]\\@

**TS-30/31**

HF=-972.6501374 a.u., NIMAG=1, -84.2700 cm^-1^

1\1\GINC-R01N12\FTS\RPBE1PBE\def2TZVP\C21H15N3\WURTHWE\22-Jan-2025\0\\

# pbe1pbe/def2tzvp opt=(ts,noeigentest,calcfc,maxstep=12) freq pop=nbo emp=gd3bj scrf=(solvent=dichloromethane)\\TS for 1,2,4-triazine-ringopening\\0,1\

C,-0.0118978856,-0.0120615103,0.0095652233\C,0.0084402202,0.0058837217,1.4068107714\

C,1.2449657291,0.0151154185,2.0599198391\C,2.4232373028,-0.0035026056,1.3340779378\

C,2.3954898716,-0.0148305621,-0.0547939974\C,1.1713848751,-0.0168073228,-0.7098509273\

C,-1.2398022518,0.0051073177,2.1482771272\N,-1.2763713994,-0.3586282795,3.4088872575\

N,-1.6522379695,-0.7505538877,4.4064101132\C,-2.5950583842,0.2450453151,1.6710964049\

C,-3.0007182751,1.3952055087,0.8701888872\C,-4.2550231168,1.4212798415,0.2532928123\

C,-4.6476392868,2.5176027388,-0.4938781208\C,-3.7945234667,3.6035997847,-0.6485630305\

C,-2.5459153232,3.5837090444,-0.0419810345\C,-2.1501713653,2.4915910994,0.7123577312\

N,-3.4806058936,-0.5746250677,2.1507511665\C,-3.9393013245,-1.3214757519,2.9996465238\

C,-4.4743462213,-2.6595206012,2.8279973462\C,-5.0429616084,-3.30593696,3.9249252204\

C,-5.5454356631,-4.5900548389,3.7937521124\C,-5.4795744919,-5.2355880999,2.5666515811\

C,-4.9117245146,-4.5997421335,1.467290061\C,-4.4111236875,-3.3177074057,1.5943446383\

H,1.1353721678,-0.029201221,-1.7932758388\H,3.3195376413,-0.0208370957,-0.6204443846\

H,3.372088857,0.0008795212,1.8583907932\H,1.2810427225,0.0477070274,3.1435261165\

H,-0.9621902681,-0.0286228933,-0.5106489093\H,-1.8751552875,4.4280698252,-0.1521284856\

H,-1.179832898,2.4893112916,1.1939097466\H,-4.9164066967,0.5685476773,0.3577564407\

H,-5.6239100579,2.5226665343,-0.9650431374\H,-4.1011474659,4.4587092652,-1.2393190436\

H,-5.0797636404,-2.7875902055,4.8756503987\H,-5.9871056856,-5.0893291372,4.6477539523\

H,-5.8714535705,-6.240952541,2.4637983299\H,-4.8618560698,-5.1090075707,0.5119871773\

H,-3.9673912292,-2.8117982219,0.7443206198\\

Version=ES64L-G16RevB.01\State=1-A\HF=-972.6501374\RMSD=4.989e-09\RMSF=6.524e-07\Dipole=-0.3083425,-0.7910057,-0.9008024\Quadrupole=1.8933339,4.6184088,-6.5117

427,8.728518,0.6329738,-3.5989577\PG=C01 [X(C21H15N3)]\\@

**31**

HF=-972.7521112 a.u. NIMAG=0

1\1\GINC-R02N08\FOpt\RPBE1PBE\def2TZVP\C21H15N3\WURTHWE\21-Jan-2025\0\

\# pbe1pbe/def2tzvp opt=(maxstep=5) geom=check guess=read freq pop=nbo

emp=gd3bj scrf=(solvent=dichloromethane)\\Triphenyl-1,2,4-triazin 32\\0,1\

N,-1.4250054261,-1.8082273813,0.3655578953\C,-1.8778259174,-0.5728689064,0.1427375254\

N,-1.0917927401,0.4974771816,0.042942303\C,0.2145419339,0.3129001587,0.0865166054\

C,0.7134873366,-1.0100752256,0.1220686437\N,-0.1333441187,-2.0159717505,0.3153690479\

C,1.056474916,1.5206557822,0.129904436\C,0.6902880108,2.6342348621,-0.6236673033\

C,1.4530637358,3.7893892051,-0.5787663331\C,2.5744103215,3.8506117622,0.2374185861\

C,2.9312003829,2.7515386264,1.0076533087\C,2.1824794758,1.5885176944,0.9490989164\

C,-3.3347987455,-0.3845919414,0.044183495\C,-4.1930575078,-1.4843192361,0.0215262142\

C,-5.5616322195,-1.2984762743,-0.0747873978\C,-6.089061748,-0.0158615172,-0.1504294148\

C,-5.2396142552,1.0823311274,-0.1300516837\C,-3.8701879334,0.9008249327,-0.033793761\

C,2.1271183546,-1.3820876462,-0.0544197748\C,2.6921239256,-2.3689803224,0.7506480211\

C,4.0149716762,-2.7422244428,0.5750489396\C,4.7820789792,-2.1444331782,-0.4153357791\

C,4.2194865641,-1.172372573,-1.2320769771\C,2.901181812,-0.7895462638,-1.0511136381\

H,4.8097637504,-0.7111369664,-2.0151195402\H,5.8163156139,-2.4375421722,-0.5532586233\

H,4.4486752653,-3.5023339473,1.2143204232\H,2.0870027428,-2.833855712,1.5193558414\

H,2.4669375728,-0.0315574787,-1.6920966963\H,3.7971694428,2.8003010132,1.6570949916\

H,2.4655349051,0.7346554352,1.5521301681\H,-0.1919477405,2.5796869956,-1.2497423271\

H,1.1704439854,4.6454233537,-1.180122797\H,3.1686567811,4.7561087383,0.276588291\

H,-3.7750115954,-2.4808376994,0.077482078\H,-6.2207423668,-2.1585452731,-0.0935580219\

H,-7.1608076582,0.1273420073,-0.2258967732\H,-5.6467376723,2.0850800966,-0.1873771879\

H,-3.2009047291,1.7512472089,-0.0130692308\\

Version=ES64L-G16RevB.01\State=1-A\HF=-972.7521112\RMSD=3.025e-09\RMSF=4.510e-06\Dipole=0.9121276,1.3518534,-0.2530653\Quadrupole=12.7745771,-4.4663634,-8.3082137,-0.940145,2.5641626,-3.1676645\PG=C01 [X(C21H15N3)]\\@

**32**

HF=-1187.4707112 a.u. NIMAG=0

1\1\GINC-R02N44\FOpt\RPBE1PBE\def2TZVP\C28H20N2\WURTHWE\30-Jul-2021\0\ \# pbe1pbe/ def2tzvp opt freq pop=nbo emp=gd3bj scrf=(solvent=dichloromethane)\\Bis-nitrilylide 33\\0,1\

C,0.3391703734,-1.3274802401,-0.3846343589\C,-0.0386380508,-0.7086701649,0.7990780719\

C,0.9133545146,-0.0777393658,1.5834950155\C,2.2536233359,-0.084515191,1.2008101747\

C,2.6276187046,-0.7159714001,0.0096255233\C,1.672276608,-1.3268376869,-0.7814567367\

C,3.2311610259,0.6176874795,2.0190301957\N,4.4383930598,0.5458759929,1.9446667426\

C,5.7200290821,0.6599971869,1.932138467\C,6.2849335626,1.7093080308,1.0664582363\

N,5.6142110621,2.7993139159,0.9632491684\C,4.9465187436,3.812571337,1.001970334\

C,3.7638309661,4.0796916731,0.1834480313\C,3.2686004815,3.1304202653,-0.7163113322\

C,2.1280326117,3.3957063438,-1.4500229128\C,1.4747562663,4.614952119,-1.3000441885\

C,1.9633982862,5.5657937955,-0.415439852\C,3.1035024924,5.2977622633,0.3264522868\

H,1.4528209925,6.514513534,-0.3004783458\H,0.579932419,4.8215280767,-1.8758858884\

H,1.7417165269,2.6527112751,-2.1381998121\H,3.7829781036,2.1825693329,-0.8282498189\

H,3.4943516321,6.0248416825,1.0284921751\H,-1.0783099891,-0.7077603,1.1039641645\

H,0.6326523526,0.4289739315,2.4991642353\H,3.6703061771,-0.7153368835,-0.2887879382\

H,1.9631893554,-1.8085575391,-1.7077191002\H,-0.407649587,-1.8096128849,-1.0048296555\

C,6.5264226947,-0.1797233862,2.8189480403\C,7.4956671905,1.5208158711,0.2635598979\

C,7.8075182478,0.2185019225,3.2095409052\C,8.556767796,-0.5670397038,4.0695020273\

C,8.0433445682,-1.7601942056,4.5595675425\C,6.7678262066,-2.1609238492,4.1818492049\

C,6.0173238935,-1.3821896473,3.3183381635\H,8.2127395929,1.1541672717,2.8442979593\

H,9.5475512091,-0.2403267388,4.3639366651\H,8.6322894255,-2.3737421437,5.2310987785\

H,6.3572075136,-3.0913003026,4.5572991264\H,5.0286318121,-1.7088456568,3.0165392409\

C,7.9585520808,0.2394141319,-0.0450613075\C,9.0957172109,0.0692930282,-0.8174229756\

C,9.7910687146,1.1707964089,-1.297258794\C,9.3345064545,2.4488890369,-0.9994089233\

C,8.1993629328,2.6239699597,-0.2274236566\H,7.4202356199,-0.627381327,0.3182512431\

H,9.438663898,-0.9326949086,-1.0488522433\H,10.6811171057,1.0351626391,-1.9003083585\

H,9.8695358218,3.316464266,-1.3685527595\H,7.8514693259,3.6239333885,0.0055829736\\ Version=ES64L-G16RevB.01\State=1-A\HF=-1187.4707112\RMSD=5.628e-09\RMSF=2.057e-06\Dipole=-1.0609681,-0.882942,-1.3331697\Quadrupole=10.5636533,-1.6214616,-8.9421917,-0.9437475,6.2433724,-2.2466975\PG=C01 [X(C28H20N2)]\\@

**TS-32/33**

HF=-1187.4635766 a.u., NIMAG=1, -77.2171 cm^-1^

1\1\GINC-R02N25\FTS\RPBE1PBE\def2TZVP\C28H20N2\WURTHWE\13-Nov-2024\0\\

# pbe1pbe/def2tzvp opt=(ts,noeigentest,readfC,maxstep=12) geom=check guess=read freq pop=nbo emp=gd3bj scrf=(solvent=dichloromethane)\\Bis-nitrilylide: TS-search for ring closure\\0,1\

C,-0.004977667,0.1245294528,0.2704427748\C,-0.0760016336,0.0665449069,1.6617713902\

C,1.1031503023,-0.0656985752,2.3937560147\C,2.3265754225,-0.1531193655,1.7486294283\

C,2.3883278279,-0.1076602932,0.3625157429\C,1.2192946974,0.0352373979,-0.3725878683\

C,-1.3629049561,0.1979739553,2.3738583538\N,-1.4545469235,1.0566564868,3.3266164384\

C,-1.8484268755,1.6405587767,4.3246467013\C,-2.3237628125,3.0168078514,4.3628362091\

C,-2.0855937375,3.9061609414,3.3096133149\C,-2.5537234304,5.2054961848,3.3792449867\

C,-3.2751501805,5.6266681871,4.4914013354\C,-3.5174615957,4.7494582965,5.539535714\

C,-3.0308027303,3.453426824,5.482029994\C,-2.5650170132,-0.5852158607,2.0934547829\

C,-2.7802151911,-1.4127739124,0.8895864045\C,-1.8479971209,-2.3684838567,0.487355284\

C,-2.0901386269,-3.1580258295,-0.6254318813\C,-3.2660919813,-3.0089351763,-1.348163661\

C,-4.2046251253,-2.0683962469,-0.946003122\C,-3.9638212963,-1.2765441313,0.1655649077\

N,-3.4993570605,-0.5258254977,2.9749099922\C,-4.1864115527,-0.1424817469,3.90933577\

C,-4.4215726444,-0.8829610884,5.1413478991\C,-4.1292949489,-2.2469696646,5.2462586222\

C,-4.3637294469,-2.9176335495,6.4326466159\C,-4.8777212111,-2.2333243726,7.5291262442\

C,-5.1682226259,-0.8793418303,7.4335339459\C,-4.9547610319,-0.2097950155,6.239388835\

H,-4.0771809205,5.0799375068,6.4065300785\H,-3.6450902781,6.644326216,4.5409259688\

H,-2.364106183,5.8936701512,2.5635533485\H,-1.5281107675,3.5667451801,2.4437856057\

H,-3.1974229588,2.759565812,6.2975256989\H,-5.5712864735,-0.3483003108,8.2877680452\

H,-5.1898580949,0.8435314342,6.1420676461\H,-3.7225240301,-2.7703907128,4.3882284522\

H,-4.1402477013,-3.9754576231,6.5098158757\H,-5.0557716786,-2.7606272477,8.4592799611\

H,-0.9338692593,-2.4994050775,1.0529264562\H,-1.3582749654,-3.8993400977,-0.9249926818\

H,-3.4526240802,-3.6273694124,-2.2184026213\H,-5.1269344455,-1.947222429,-1.5024388992\

H,-4.6955449472,-0.5400888576,0.4785097663\H,-0.9110317427,0.2482255383,-0.30956253\

H,1.2602255712,0.0850362948,-1.4546011164\H,3.344406795,-0.1778348908,-0.143013193\

H,3.2344904326,-0.2614123771,2.3307989091\H,1.0542986063,-0.1036760463,3.4762954297\\ Version=ES64L-G16RevB.01\State=1-A\HF=-1187.4635766\RMSD=9.323e-09\RMSF=9.811e-07\Dipole=0.1228779,-0.1099537,-0.2196757\Quadrupole=-7.7695732,2.9282189,4.8413542,-4.0702824,-7.7660103,1.140952\PG=C01 [X(C28H20N2)]\\@

**33**

HF=-1187.6213646 a.u., NIMAG=0

1\1\GINC-R08N35\FOpt\RPBE1PBE\def2TZVP\C28H20N2\WURTHWE\14-Nov-2024\0\

\# pbe1pbe/def2tzvp opt=(readfC,maxstep=12) geom=check guess=read freq

pop=nbo emp=gd3bj scrf=(solvent=dichloromethane)\\1,4-Diazine 34\\0,1\

C,-3.2926439424,-1.4192334097,0.7721090328\C,-2.2893223076,-1.557935722,-0.1861756676\

C,-2.4056273736,-2.5600868944,-1.147826215\C,-3.5139086489,-3.3914265263,-1.1654258526\

C,-4.5124183115,-3.2421989748,-0.2123918914\C,-4.3943386775,-2.2583910701,0.7605085819\

C,-1.0918134997,-0.6973256797,-0.1645767503\C,-1.1057918311,0.6780502219,0.1475785201\

N,0.0369450847,1.2834398843,0.4437963101\C,1.1933466572,0.6470484972,0.3105602804\

C,1.2086397668,-0.6272763215,-0.2937733547\N,0.0652782894,-1.2831865946,-0.4439883161\

C,2.388665177,1.3337121838,0.8348104773\C,2.5098684384,2.7155413115,0.6989074706\

C,3.615982238,3.3769914769,1.2075485489\C,4.6072989327,2.6680121561,1.8725358171\

C,4.4841918799,1.2936561858,2.0285861779\C,3.384736954,0.6297473917,1.5104430748\

C,-2.3179915502,1.5181063155,0.1515638434\C,-3.3047096688,1.3623851766,-0.8212391225\

C,-4.420601399,2.1826350146,-0.8258865093\C,-4.569624697,3.1642251211,0.1450046711\

C,-3.5878248489,3.330391612,1.1124889206\C,-2.4653938119,2.5180704918,1.1112288273\

C,2.4230582529,-1.2934806197,-0 .8002087578\C,2.5657497922,-2.6730531465,-0.6622664578\

C,3.6903475946,-3.3155441203,-1.1544105385\C,4.6791536033,-2.5897477779,-1.8048345676\

C,4.5349831724,-1.217677453,-1.9629481654\C,3.4168562323,-0.5726179284,-1.4612135668\

H,5.2960394815,-0.6470933391,-2.4824460673\H,5.5579267003,-3.0924225687,-2.1919043938\

H,3.7957033429,-4.3871504868,-1.0299822138\H,1.7867423371,-3.2333605609,-0.159665639\

H,3.3086721083,0.4975830864,-1.5907296195\H,5.2471043435,0.7360112394,2.5592912208\

H,3.2928987504,-0.4421643227,1.6384156223\H,1.7289277421,3.2626021719,0.1848449153\

H,3.7049005808,4.4502601208,1.0846238082\H,5.4715794563,3.1855006323,2.2725008405\

H,-3.1926821835,0.6006093299,-1.583435146\H,-5.1760811241,2.0570045123,-1.5927754841\

H,-5.4466834591,3.8010254469,0.1444083923\H,-3.6969681492,4.0964396757,1.8715546461\

H,-1.6917946921,2.6460342945,1.8586059001\H,-3.2047809982,-0.655792331,1.5358087408\

H,-5.1629668106,-2.1457997391,1.5162767885\H,-5.3784125348,-3.8938526701,-0.2245625705\

H,-3.5988840052,-4.1590780743,-1.9259604087\H,-1.6191353837,-2.6747062193,-1.8838281528\\ Version=ES64L-G16RevB.01\State=1-A\HF=-1187.6213646\RMSD=4.831e-09\RMSF=5.095e-06\Dipole=0.0000754,-0.0000052,0.0000822\Quadrupole=10.9020763,-1.7617745,-9.1403018, 0.0860122,0.122664,2.8996924\PG=C01 [X(C28H20N2)]\\@

Table S9: CCSD(T)/cc-pvtz -Calculations of the diazomethyl radical and its dimer **1,2-bis(diazo)ethane 1a ^[10-15]^**

| **Species** | **E_tot_ [a.u.]** | **E_rel_ [kcal/mol]** | **G_298_ [a.u.]** | **E_rel_ [kcal/mol]** |
| --- | --- | --- | --- | --- |
| Diazomethane | -147.83512 |  | -147.84024 |  |
| 2 Diazomethane | -295.63316 | 0.00 | -295.68048 | 0.00 |
| Bis-diazoethane **1a** | -295.80963 | 19.14 | -295.79312 | -70.67 |

**Gaussian Archive Entries**

(Total energies (a.u.), number of imaginary frequencies)

**Diazomethyl radical**

CCSD(T)=-147.835124 a.u., NIMAG=0

1\1\GINC-R08N24\FOpt\UCCSD(T)-FC\CC-pVTZ\C1H1N2(2)\WURTHWE\06-Jan-2025 \1\\# ccsd(t)/cc-pvtz opt=(maxstep=5) freq pop=nbo scrf=(solvent=dichloromethane)\\

diazomethyl radical\\0,2\

N\N,1,nn2\C,2,cn3,1,cnn3\H,3,hc4,2,hcn4,1,dih4,0\\nn2=1.15094595\ cn3=1.29023233\ cnn3=170.02892669\hc4=1.0895961\hcn4=112.54379473\dih4=-180.34499868\\

Version=ES64L-G16RevB.01\State=2-A\HF=-147.2790144\MP2=-147.7750285\MP3=-147.7862475\MP4D=-147.7993601\MP4DQ=-147.7849144\PUHF=-147.3032942\PMP2-0=-147.7962075\PMP3-0=-147.801992\MP4SDQ=-147.7973104\CCSD=-147.8054879\CCSD(T)=-147.835124\S2=1.025613\S2-1=0.947582\S2A=0.800449\RMSD=4.481e-09\RMSF=1.261e-05\PG=C

01 [X(C1H1N2)]\\@

Sum of electronic and thermal Free Energies= -147.840238 a.u.

**1,2-bis(diazo)ethane** **1a**

CCSD(T) =-295.8096311 a.u. , NIMAG=0

1\1\GINC-R08N19\FOpt\RCCSD(T)-FC\CC-pVTZ\C2H2N4\WURTHWE\26-Mar-2025\1\

\# ccsd(t)/cc-pvtz opt=(maxstep=4,maxcyc=60,readfc) geom=check guess=read nosym freq scrf=(solvent=dichloromethane)\\1,2-Bis-diazoethane\\0,1\

H\C,1,ch2\C,2,cc3,1,cch3\H,3,hc4,2,hcc4,1,dih4,0\N,3,nc5,2,ncc5,1,dih5,0\ N,3,nc6,2,ncc6,1,dih6,0\ N,2,nc7,3,ncc7,5,dih7,0\N,2,nc8,3,ncc8,5,dih8,0\\ch2=1.07925141\cc3=1.46344776\ cch3=126.3155796\hc4=1.0792437\hcc4=126.33152558\dih4=86.00520781\nc5=1.31357605\ ncc5=118.87282702\dih5=-90.82195015\nc6=2.45414683\ ncc6=118.72595386\dih6=-90.87268536\ nc7=1.31354146\ ncc7=118.87573732\dih7=92.46123954\nc8=2.4540892\ncc8=118.73321169\ dih8=92.40657751\\

Version=ES64L-G16 RevB.01\HF=-294.6391235\MP2=-295.7527997\MP3=-295.7344432\MP4D=-295.7699371\MP4DQ=-295.7331382\MP4SDQ=-295.7514172\CCSD=-295.7459039\CCSD(T)

=-295.8096311\RMSD=5.893e-09\RMSF=2.083e-05\PG=C01 [X(C2H2N4)]\\@

Sum of electronic and thermal Free Energies= -295.793119 a.u.

# References

[1] Gaussian 16, Revision B.01, M. J. Frisch, G. W. Trucks, H. B. Schlegel, G. E. Scuseria, M. A. Robb, J. R. Cheeseman, G. Scalmani, V. Barone, G. A. Petersson, H. Nakatsuji, X. Li, M. Caricato, A. V. Marenich, J. Bloino, B. G. Janesko, R. Gomperts, B. Mennucci, H. P. Hratchian, J. V. Ortiz, A. F. Izmaylov, J. L. Sonnenberg, D. Williams-Young, F. Ding, F. Lipparini, F. Egidi, J. Goings, B. Peng, A. Petrone, T. Henderson, D. Ranasinghe, V. G. Zakrzewski, J. Gao, N. Rega, G. Zheng, W. Liang, M. Hada, M. Ehara, K. Toyota, R. Fukuda, J. Hasegawa, M. Ishida, T. Nakajima, Y. Honda, O. Kitao, H. Nakai, T. Vreven, K. Throssell, J. A. Montgomery, Jr., J. E. Peralta, F. Ogliaro, M. J. Bearpark, J. J. Heyd, E. N. Brothers, K. N. Kudin, V. N. Staroverov, T. A. Keith, R. Kobayashi, J. Normand, K. Raghavachari, A. P. Rendell, J. C. Burant, S. S. Iyengar, J. Tomasi, M. Cossi, J. M. Millam, M. Klene, C. Adamo, R. Cammi, J. W. Ochterski, R. L. Martin, K. Morokuma, O. Farkas, J. B. Foresman, and D. J. Fox, Gaussian, Inc., Wallingford CT, 2016.

[2] J. P. Perdew, K. Burke, M. Ernzerhof, *Phys. Rev. Lett.* **1996**, *77*, 3865–3868.

[3] J. P. Perdew, K. Burke, M. Ernzerhof, *Phys. Rev. Lett.* **1997**, *78,* 1396.

[4] C. Adamo V. Barone, *J. Chem. Phys.* **1999**, *110*, 6158–6169.

[5] M. Ernzerhof, G. E. Scuseria, *J. Chem. Phys.* **1999**, *110*, 5029–5036.

[6] R. Weigend, R. Ahlrichs, *Phys. Chem. Chem. Phys.* **2005**, *7*, 3297–3305.

[7] S. Grimme, S. Ehrlich, L. Goerigk, *J. Comput. Chem.* **2011**, *32*, 1456–1465.

[8] S. Grimme, A. Hansen, J. G. Brandenburg, C. Bannwarth, *Chem. Rev*. **2016**, *116*, 5105–5154.

[9] J. Tomasi, B. Mennucci, R. Cammi, *Chem. Rev.* **2005**, *105*, 2999–3093.

[10] R. J. Bartlett, G. D. Purvis III, *Int. J. Quantum Chem.* **1978**, *14*, 561–581.

[11] J. A. Pople, R. Krishnan, H. B. Schlegel, J. S. Binkley, *Int. J. Quantum Chem.* **1978**, *14*, 545–560.

[12] J. A. Pople, M. Head-Gordon, K. Raghavachari, *J. Chem. Phys.* **1987**, *87*, 5968–5975.

[13] G. D.Purvis III, R. J. Bartlett, *J. Chem. Phys.* **1982,** *76,* 1910–1918.

[14] G. E.Scuseria, C. L. Janssen, H. F. Schaefer III, *J. Chem. Phys.* **1988**, *89*, 7382–7387.

[15] G. E.Scuseria, H. F. Schaefer III, *J. Chem. Phys.* **1989***,* *90*, 3700–3703.
